# Telechelic sequence-defined oligoamides: their step-economical synthesis, depolymerization and use in polymer networks†

**DOI:** 10.1039/d3sc04820a

**Published:** 2024-01-15

**Authors:** Irene De Franceschi, Nezha Badi, Filip E. Du Prez

**Affiliations:** a Polymer Chemistry Research Group, Centre of Macromolecular Chemistry (CMaC), Department of Organic and Macromolecular Chemistry, Faculty of Sciences, Ghent University 9000 Ghent Belgium nezha.badi@ugent.be filip.duprez@ugent.be

## Abstract

The application of sequence-defined macromolecules in material science remains largely unexplored due to their challenging, low yielding and time-consuming synthesis. This work first describes a step-economical method for synthesizing unnatural sequence-defined oligoamides through fluorenylmethyloxycarbonyl chemistry. The use of a monodisperse soluble support enables homogeneous reactions at elevated temperature (up to 65 °C), leading to rapid coupling times (<10 min) and improved synthesis protocols. Moreover, a one-pot procedure for the two involved iterative steps is demonstrated *via* an intermediate quenching step, eliminating the need for in-between purification. The protocol is optimized using γ-aminobutyric acid (GABA) as initial amino acid, and the unique ability of the resulting oligomers to depolymerize, with the formation of cyclic γ-butyrolactame, is evidenced. Furthermore, in order to demonstrate the versatility of the present protocol, a library of 17 unnatural amino acid monomers is synthesized, starting from the readily available GABA-derivative 4-amino-2-hydroxybutanoic acid, and then used to create multifunctional tetramers. Notably, the obtained tetramers show higher thermal stability than a similar thiolactone-based sequence-defined macromolecule, which enables its exploration within a material context. To that end, a bidirectional growth approach is proposed as a greener alternative that reduces the number of synthetic steps to obtain telechelic sequence-defined oligoamides. The latter are finally used as macromers for the preparation of polymer networks. We expect this strategy to pave the way for the further exploration of sequence-defined macromolecules in material science.

## Abstract 2

Sequence-defined oligoamides are prepared using functionalized unnatural amino acids. Their depolymerization and thermal properties are reported. A bidirectional growth method is applied for the design of telechelic oligoamides to form networks.

## Graphical abstract

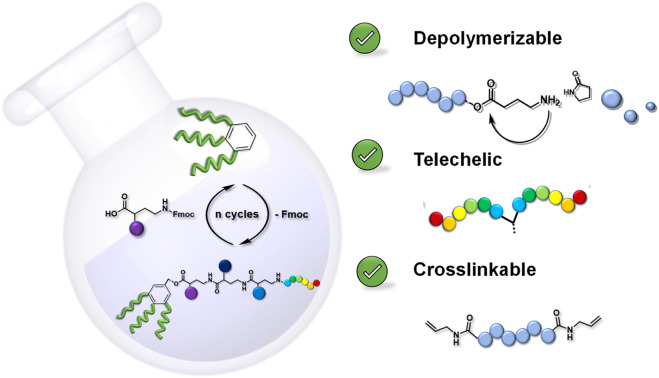

## Introduction

Originally, chemists embarked upon the exploration of sequence-defined polymers to mimic the extreme control and precision found in biomacromolecules (*i.e.*, proteins and oligonucleotides), and go towards a variety of uniform synthetic polymers, often completely stereo-controlled.^1–5^ Nowadays, these macromolecules, exhibiting by definition a discrete molecular weight, a uniform structure and defined architecture, could be prepared through two main methods, being solid-^6–10^ and liquid-phase synthesis.^11–13^ The first one makes use of an insoluble polymer matrix that can be functionalized with a cleavable linker from which the iterative attachment of monomers starts.^14^ Iterative protocols in solution have gained much interest because of their ability to facilitate increased reactions scales and easy monitoring of the reaction process.^11,15–20^ While an intrinsic drawback associated with liquid-phase synthesis remains the need for a labor-intensive workup after each synthetic step (*e.g.*, column chromatography), different methods have been described in literature to overcome this hurdle.^13,21–23^ In this context, it has been demonstrated that a hydrophobic uniform soluble support could be used for the scalable synthesis of peptides in multigram scales.^24^

This strategy has recently been adapted by our research group to scale-up the synthesis of oligo(ester urethane)s *via* a thiolactone chemistry-based iterative protocol, while facilitating the purification and monitoring of the intermediate reaction steps.^25^

A variety of chemical backbones that could be synthesized *via* iterative protocols have been explored in the last years.^26^ Among them, sequence-defined oligomers containing amide bonds are gaining major interest in many areas of research. For instance, Hartmann and co-workers synthesized sequence-defined polyamides on solid-phase in a fast way, using specific building blocks.^7,27^ Moreover, such investigation on sequence-defined oligoamides was not limited to pure amide linkages, but bonds of different chemical nature were also explored to introduce structural variability. For example, the same group developed sequence-defined oligoamides incorporating secondary or tertiary amines in the backbone *via* diamines.^28,29^ These poly(amidoamine)s received attention for their excellent biocompatibility together with the absence of inherent immunogenicity, and they have been explored in nanocarrier applications for binding studies. Other groups also reported the synthesis of different sequence-defined oligoamides such as the famous class of peptoids,^30–32^ oligo-l-glutamamides^33^ and oligo(ethylene amino) acids.^34^

When considering alternative monomers for the preparation of sequence-defined oligoamides, various unnatural amino acids, particularly β- and γ-amino acids, could be interesting candidates.^35–37^ The iterative coupling of these monomers allows for the synthesis of oligomers that can be considered as examples of peptidomimetic structures, specifically belonging to the class of β- and γ-peptides. The interest in such structures increased over the years because of their peculiar (self)assembling properties and proteolytic resistance as a result of the extra carbon atoms, compared to their analogue α-peptides.^36,38,39^ However, these sequence-defined oligoamides have usually been prepared in small scale (<100 mg) using a solid-phase approach.

Interestingly, γ-amino acids can provide a backbone for a sequence-defined analogue to nylon 4, which is typically obtained *via* ring-opening polymerization of 2-pyrrolidinone, a five-membered ring lactam.^40,41^ Nylon 4 is a particularly promising polyamide because it shows tenacity, elongation, elastic recovery and moisture similar to cotton and other commercially available polyamide fibers. Moreover, there is an increasing interest in cross-linked polyamides because of their high intermolecular force and insolubility in organic solvents.^42^ Despite the fact that these polymers exhibit promising mechanical and thermal properties, the synthesis of the corresponding sequence-defined polyamides and their use within a material application context have, to the best of our knowledge, never been reported. When prepared in larger scale, sequence-defined macromolecules could provide an important tool to perform a systematic investigation of the correlation between the molecular structure and topology and the macroscopic properties of bulk materials, as demonstrated by Alabi and co-workers.^19^ Therefore, scalability of the synthesis of sequence-defined macromolecules is being intensively investigated by different research groups including ours.^23,25,43^ Among the major hurdles to the large-scale synthesis of sequence-defined oligoamides, is the need for a large number of synthesis and purification steps, which greatly reduces the overall yield. In order to limit the number of synthetic steps and perform a faster and scalable synthesis, other approaches are generally applied, such as a bidirectional^44,45^ or an iterative exponential^46–48^ growth.

**Scheme 1:**
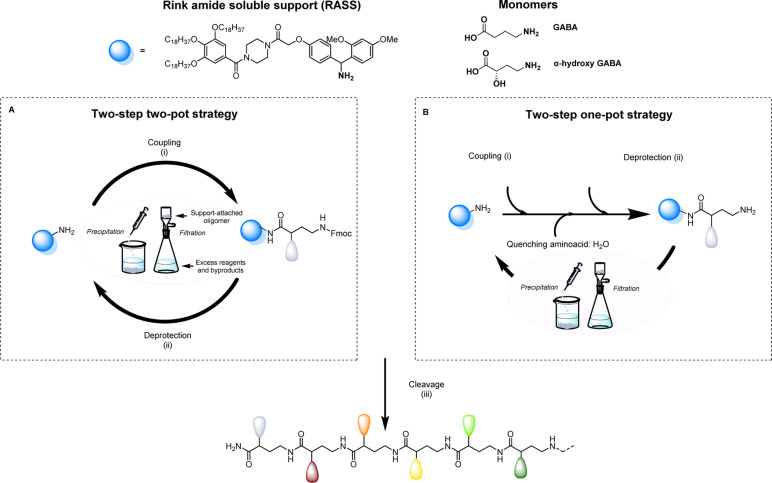
Protocols used for the synthesis of sequence-defined oligoamides, starting from a Rink amide-functionalized soluble support and using γ-aminobutyric acid (GABA) or α-hydroxy GABA derivatives. (A) Two-step two-pot strategy. (B) Two-step one-pot strategy. Conditions: (i) coupling with amino acid, carbodiimide, 4-dimethylaminopyridine in CH_2_Cl_2_ or CHCl_3_; (ii) deprotection with 5% 1,8-diazabicyclo[5.4.0]undec-7-ene; 1% piperazine in CH_2_Cl_2_ or CHCl_3_; (iii) cleavage of the sequence-defined oligoamide with trifluoroacetic acid and triisopropylsilane in CH_2_Cl_2_.

Aiming to facilitate the investigation of sequence-defined oligoamides (SDOs) within a material context, we herein report a scalable solution phase synthesis approach. Key to this approach is the use of a Rink amide-functionalized soluble support (RASS), which facilitates intermediate purification steps and the isolation of the final oligomers after cleavage (Scheme 1). This strategy is based on fluorenylmethyloxycarbonyl (Fmoc) chemistry, widely used for peptide synthesis, and each coupling step is optimized with γ-aminobutyric acid (GABA), a cheap non-proteinogenic amino acid that has been isolated from tomato, lactic acid bacteria and other sources and resembles the repeating unit forming nylon 4. The two-step iterative synthesis of SDOs will initially be described using a two-pot process (Scheme 1a), after which a series of optimization efforts enabled both steps to take place in a one-pot fashion without any form of intermediate purification (Scheme 1b).

In a next step, a unique step-economical, more sustainable bidirectional growth approach will be described, depicting the possibility to form telechelic structures for network formation. In this context, thermal stability of the obtained oligomers and networks will be reported.

In order to further show the versatility of the proposed strategy, a series of monomers bearing different functional groups or protecting moieties is prepared starting from (S)-4-amino-2-hydroxybutanoic acid (α-hydroxy GABA). As an additional feature, sequence-controlled depolymerization of the linear structures is also described in this study.

## Results and discussion

### Preparation of sequence-defined oligoamides

All sequence-defined oligoamides, starting from unnatural amino acids, have been prepared in solution using a uniform RASS-based soluble support. This support is a gallic acid derivative bearing C_18_ alkyl chains that allows an easy purification after each synthetic step *via* precipitation in polar protic solvents as described in our previous work for the scale-up synthesis of thiolactone-based sequence-defined macromolecules^25^ (Fig. S1† for ^1^H NMR characterization of RASS). This support was functionalized with a Rink amide linker that can be cleaved under relatively mild acidic conditions (Scheme 1).^49^ The choice of the linker is crucial, as it needs to be compatible with the applied Fmoc chemistry and thus acid cleavable. The optimization of the protocol was first performed using a two-step two-pot strategy (Scheme 1A). For the coupling step, the RASS was reacted with a small excess of GABA (later referred to as G) as the starting monomer. Only 1.2 eq. of the chosen GABA amino acid were required, and the coupling was performed using 1.2 eq. carbodiimide and 0.05 eq. of 4-dimethylaminopyridine (DMAP) as a catalyst.

The reaction was tested with two different carbodiimides (1-ethyl-3-(3-dimethylaminopropyl)carbodiimide hydrochloride (EDC-HCl) and *N*,*N*′-diisopropylcarbodiimide (DIC)) but both coupling agents showed a similar performance for the activation of the carboxylic acid in the used conditions. After attachment of the first monomer to the RASS, the purification was achieved by precipitation in methanol and an additional washing with the same solvent. In this way, the monomer attached to the support (Fmoc-GABA-RASS) was recovered after drying in the form of a white powder. Its structure and purity were confirmed by ^1^H NMR and matrix-assisted laser desorption/ionization-time-of-flight (MALDI-ToF) (Fig. S2†). In contrast, the excess of monomer, coupling agent and potential side products such as urea derivatives, all soluble in methanol, were discarded in the filtrate. Subsequently, the Fmoc deprotection step was investigated in CHCl_3_ with two different bases. First, the standard solution used in solid-phase synthesis, meaning 20% piperidine in DMF, was tested. However, the removal of piperidine was not complete after simple precipitation in methanol.

Since remaining traces of piperidine could lead to unwanted deprotection of the amino acid introduced at the next step, thereby causing multiple undesired couplings, an alternative deprotection mixture was tested. The latter contained 1% 1,8-diazabicyclo[5.4.0]undec-7-ene (DBU) and 5% piperazine to consume the dibenzofulvene adduct. In this case, both bases could be removed successfully *via* precipitation in methanol and the amino-terminated GABA-RASS was collected in the form of a white powder purity confirmed *via* MALDI-ToF and ^1^H-NMR (Fig. S3†). During the preparation of sequenced-defined macromolecules, the overall yield is often a factor that limits their further use in a broad scope of applications. Moreover, the reduction of purification steps is resulting in less solvent waste and therefore a more sustainable design process. For this reason, the possibility to perform the coupling and deprotection step in a one-pot fashion without intermediate purification, was investigated (Scheme 1B). For that purpose, the coupling of GABA to the RASS was again performed using the same conditions as described above and monitored *via* online infrared (IR) spectroscopy by checking the formation of the amide and the simultaneous disappearance of DIC (Fig. S4†). Then, a ‘quenching step’ consisting in the introduction of 5 eq. of water (compared to the amino acid content) was directly performed in the reaction medium to deactivate the adduct between the amino acid and the carbodiimide, in order to avoid further couplings. This quenching could again be followed *via* IR spectroscopy by checking the gradual disappearance of DIC. Subsequently, piperazine (5 wt%) is added in the reaction mixture to further consume the amino acid, followed by DBU (1 wt%) to finally deprotect the coupled amino acid. An aliquot of the reaction mixture was taken 5 min after the addition of DBU and analyzed *via* MALDI-ToF (data not shown). Interestingly, this analysis already indicated complete deprotection of the Fmoc-moiety, together with the formation of the targeted structure without side products resulting from multiple couplings.

At the end of the deprotection, the GABA-RASS product was then purified *via* precipitation in methanol to remove all the reagents and dried.

Cleavage from the support was then successfully achieved in an acidic solution containing TFA and triisopropylsilane (TIPS) as carbocation scavenger (*i.e.*, CH_2_Cl_2_ : TFA : TIPS in a ratio 50 : 47.5 : 2.5). After reacting the RASS-GABA in this acidic solution for 2 h, complete cleavage of the RASS was confirmed by analyzing an aliquot of the crude mixture by MALDI-ToF (see Fig. S5†). In order to recover the cleaved monomer, a precipitation in methanol was first performed. In this case, the filtrate containing the monomer was collected, and the solvent was concentrated before performing a precipitation in diethyl ether to obtain the pure monomer in the form of a white powder (the complete procedure is given in the ESI-Section A†).

**Fig. 1:**
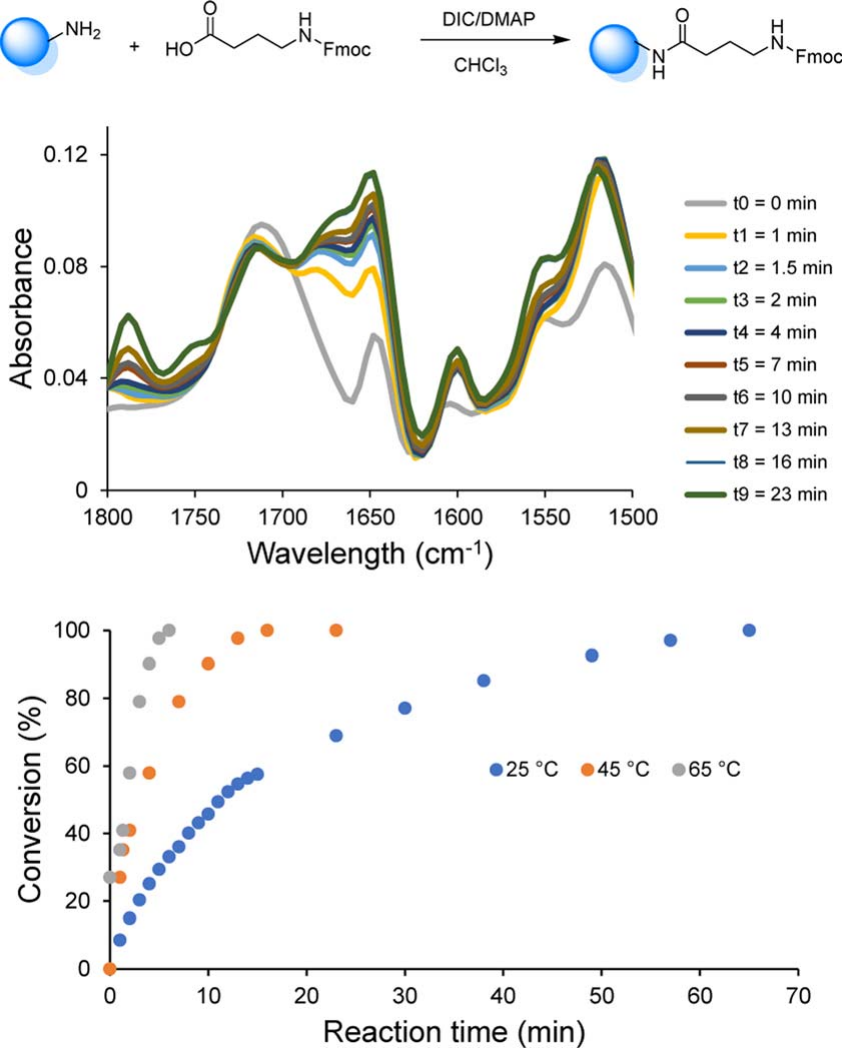
Coupling step of Fmoc-GABA on the RASS in CHCl_3_ followed by online-IR at 45 °C (top) and resulting conversion measured by following the amide bond formation at 1648 cm^−1^ in a temperature window between 25 and 65 °C (bottom).

Since the use of RASS enables to perform the reaction in solution at higher temperatures, the influence of temperature (25, 45 and 65 °C) on the coupling rate was subsequently investigated *via* online-IR spectroscopy in order to determine the minimum time required to reach full conversion (Fig. 1 and S4†).

All the reactants were dissolved in CHCl_3_ and the reactions were initiated upon the addition of DIC. The coupling kinetics were investigated by following the formation of the amide bond (peak at 1648 cm^−1^). When the chosen amide peak was no longer increasing, a sample from the reaction mixture was collected for MALDI-ToF analysis to confirm the full conversion. This investigation demonstrated the possibility to significantly increase the rate of the coupling step. Indeed, near-quantitative conversion was only achieved after 70 min at room temperature, while complete conversion was observed within 30 min at 45 °C and within less than 10 min at 65 °C (*i.e.*, under reflux). Moreover, all results showed that the deprotection step was already complete after 5 min at room temperature and an increase of temperature was therefore not further considered for this step.

### Comparison of the RASS-based protocol with solid-phase synthesis

In order to prove the effectiveness of the protocol in solution, two trimers of GABA (*i.e.*, GGG) were prepared using either solid-phase (SP) synthesis (see ESI† for the synthesis protocol and Fig. S6† for the liquid chromatography-mass spectrometry (LC-MS) analysis), or in solution with the two-step one-pot protocol (see Fig. S7† for GGG prepared in solution). The soluble RASS-support with a loading capacity of 0.77 mmol g^−1^ was therefore compared to a PS resin having a similar loading capacity (0.74 mmol g^−1^) (Table 1). Although a relatively comparable reaction time was observed for both approaches, solid-phase synthesis remains the fastest method because of the faster workup (*i.e.*, *via* simple washing and filtration) compared to solution-phase, since the latter requires precipitation and drying steps. However, the synthesis on solid-phase requires stronger coupling agents and higher excess of reactants.

**Table 1:** Comparison of the protocol using RASS or a solid-phase (SP)-based approach

Method	Loading (mmol g^−1^)	Solvent	Coupling^a^ (min)	Deprotection (min)	Amino acid/coupling agents eq.	Purification	Scale
RASS	0.77	CH_2_Cl_2_/CHCl_3_	10–70	<5	1.2–2	Precipitation	g
SP	0.74	DMF	30	8 (×2)	3–5	Washing	mg

a Coupling time indicated with hexafluorophosphate azabenzotriazole tetramethyluronium : amino acid : di-isopropylethylamine (5 : 5 : 10). More than 1 h was required with the DIC and DMAP mixture used for RASS.

Indeed, when DIC and DMAP were tested on solid-phase, non-complete couplings were observed after 90 min, whereas a stronger coupling agent, typically used in peptide synthesis showed complete coupling after 30 min. It is important to mention that the use of DIC/DMAP might cause undesired racemization of amino acids as a drawback. Therefore, coupling agents used in solid-phase synthesis, such as hexafluorophosphate azabenzotriazole tetramethyl uronium (HATU) and others, may be required when enantiomeric purity would be targeted.

However, the use of RASS has demonstrated clear advantages, as it allows the use of a much lower excess of reagents, while still showing relatively similar coupling times at room temperature. Moreover, faster coupling can be obtained by increasing the temperature, as long as the amino acids used are stable in those conditions.

### Depolymerization of sequence-defined oligoamides *via* ring-closing

The oligoamide backbone, obtained *via* the use of the unnatural amino acids GABA, provides the same backbone as the one of nylon 4. Therefore, we envisaged that it could be used for a controlled depolymerization process. Indeed, nylon 4 possesses a low ceiling temperature compared to more stable nylons 6, 6,6 or 10.^50^ The latter have been intensively used for industrial products because of their higher stability in applications that necessitate high temperature, but their degradation and depolymerization remains challenging.^51–54^

On the other hand, nylon 4 has been reported to be fully reverted to the starting monomer when it reaches its melting temperature (260–265 °C).^41^ Cyclization proceeds *via* an intramolecular, irreversible addition–elimination of the terminal amino group onto the neighboring amide moiety. Therefore, we expected the ring closing depolymerization of the sequence-defined oligoamides to also occur. Indeed, 5-membered rings are forming at low entropic cost due to the low amount of bond distortion.^55^

Moreover, the sequence composition of the backbone could be used as a tool to tune the degradation, *e.g.*, by using different monomers along the backbone.

**Fig. 2:**
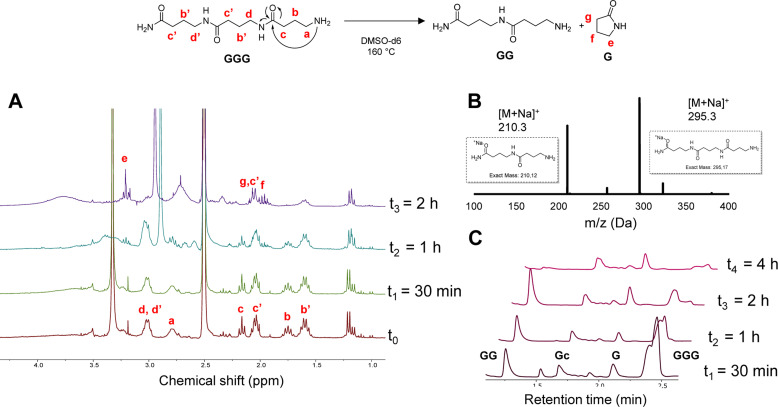
Ring-closing depolymerization of GGG; (A) ^1^H-NMR in DMSO-d_6_ recorded at different time intervals; (B) MALDI-ToF analysis at t_2_; (C) LC-MS at different time intervals.

In order to investigate the depolymerization mechanism, the trimer composed of only GABA (GGG), synthesized *via* RASS (Fig. S7†) and composed of an amide and amine end group, was dissolved in DMSO-d_6_ and heated to 160 °C. The depolymerization was monitored without adding any acid or base catalyst, in contrast to common methods currently used for polycaprolactams (Fig. 2). The depolymerization kinetics were investigated at 160 °C after 0, 30 min, 1 h and 2 h in DMSO-d_6_. From ^1^H NMR analysis, it can be noticed that the signals at 1.7 and 2.2 ppm, attributed to the –CH_2_ protons in β and γ position relative to the amine, get consumed as a function of time, ascribed to the gradual loss of the terminal GABA monomer, either *via* ring-closing or hydrolysis.

In order to confirm the mechanism, a MALDI-ToF analysis of the crude mixture was performed after 1 h (t_2_). The result showed the presence of both dimer and trimer structures in the mixture, indicating a slow depolymerization. Moreover, the new peaks appearing in the ^1^H-NMR spectra at 2.1 and 3.2 ppm can be attributed to the –CH_2_ protons of the cyclic pyrrolidinone structure, confirming the possibility of ring-closing depolymerization rather than hydrolysis. LC-MS analysis was performed on the same sample to further confirm the depolymerization.

These LC-MS analyses (after 30 min, 1 h, 2 h and 4 h) indicated that the GGG peak at 2.4 min ([M + H]^+^ = 273.2) was consumed over time, while 1-mer G ([M + H]^+^ = 103.08), cyclized GABA (Gc) ([M + H]^+^ = 86.2) and dimer GG ([M + H]^+^ = 189.2) started forming (see Fig. S8† for MS correspondence of the LC peaks). Interestingly, both GGG and GG were almost fully consumed after 4 h, whereas the Gc peak at 1.7 and G peak at 2.1 min remained. These results seem to show that the primary amine group plays a major role in the backbiting process. In other words, changing this end-group might influence or inhibit the depolymerization, as also reported in the literature for nylon 4.^56^

The precise control of the chemical composition of nylon 4, for example by alternating it with compounds used for nylon 6, can lead to slower degradation kinetics while still keeping good mechanical properties.^56,57^

Moreover, in sequence-defined oligomers, more rigid and conjugated monomeric structures can be introduced to tune or interrupt the process of depolymerization. For example, aromatic rings, which limit the rotational freedom of the chain and therefore block the ring-closure, can be added as monomers in these sequence-defined oligoamides to inhibit the process of depolymerization.

**Fig. 3:**
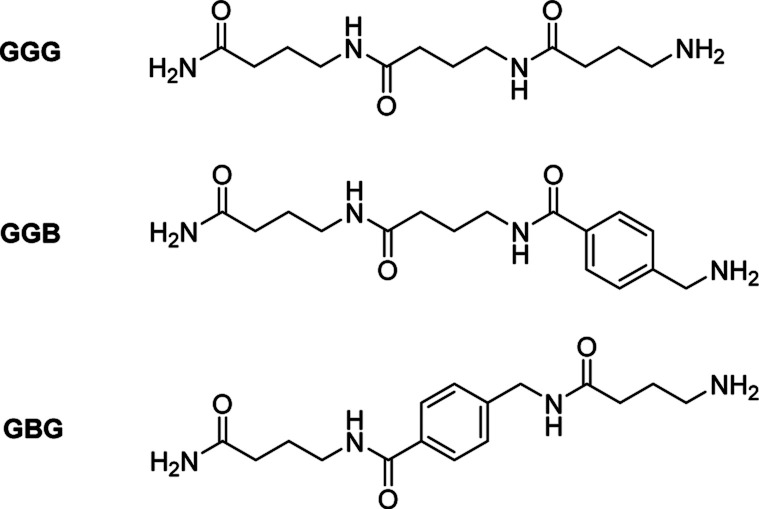
Structure of trimers GGG, GGB and GBG.

In order to prove this, an aromatic molecule (*i.e.*, 4-aminomethyl benzoic acid (B)) was introduced as a comonomer of GABA serving as an effective “blocker” at two different positions in the resulting trimers (*i.e.*, in the middle (GBG) and at the end (GGB) of the chain, Fig. 3, S9†).

The goal was to prove the effect of the position of the aromatic molecule on the depolymerization process: while the GBG-trimer was expected to undergo only one ring-closing step, thus stopping the degradation at the level of the GB dimer, the GGB should not depolymerize under these conditions. After NMR-analysis of the two trimers GBG and GGB, it could indeed be demonstrated that the GBG trimer only degrades to the dimer stage, whereas the GGB trimer fails to undergo ring-closing depolymerization, even after 2 h (Fig. S10 and S11†).

### Synthesis of a library of monomers bearing different functional groups

The use of GABA as a monomer does not allow for the introduction of functional moieties in the side chain of the sequence-defined oligoamides, for example to introduce cross-linkable moieties (*vide infra*). Therefore, a GABA derivative has been utilized, *i.e.*, 4-amino-2-hydroxybutanoic acid (α-hydroxy GABA), which is industrially synthesized as a precursor for some antibiotics.^58^ This unnatural amino acid, resembling GABA but with an additional hydroxyl moiety in the alpha-position, can be further functionalized to create a library of monomers. Besides the fact that α-hydroxy GABA is an interesting precursor as it contains three different reactive moieties (alcohol, carboxylic acid and amine) that could be functionalized in an orthogonal way, it could also be used to introduce chirality in the sequence thanks to the presence of a chiral center (not further investigated).

**Scheme 2:**
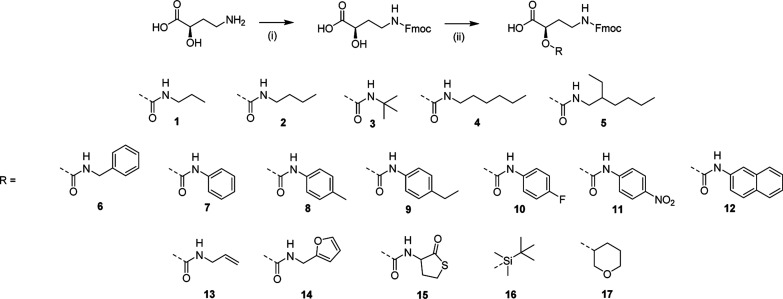
Reaction scheme for the synthesis of a library of monomers, all derived from α-hydroxy GABA. (i) Fmoc-Cl, Na_2_CO_3_ water/dioxane; (ii) alcohol–isocyanate reaction with Zr(acac)_4_ as catalyst (for compound 1 to 15) or reaction with *tert*-butyldimethylsilyl chloride (TBDMS-Cl) and 3,4-dihydropyran (DHP) to form protected compound 16 and 17.

All monomers were prepared using two straightforward synthetic steps, and an overview of the library can be found in Scheme 2.

The amine group of α-hydroxy GABA is first protected using fluorenylmethyloxycarbonyl chloride (Fmoc-Cl) in the presence of sodium carbonate (Na_2_CO_3_) as a base (see Scheme S1† and Fig. S12†).

Afterwards, the free alcohol can be either functionalized with isocyanates (compound 1 to 15) or protected with two different protecting groups (*i.e.*, *tert*-butyldimethysilyl for compound 16 and dihydropyran (DHP) for 17).

The urethane bond formation occurs orthogonally by means of a specific zirconium catalyst (zirconium(iv) acetylacetonate, Zr(acac)_4_), that coordinates and activates the alcohol moiety of the amino acid to react with the isocyanate, limiting reaction of the isocyanate with water and formation of side products.^59^

The ease of the two reaction steps as well as the purification allowed for the synthesis of a wide library of monomers, in high yields (around 90%). Different commercially available isocyanates were used, which resulted in the formation of monomers bearing either hydrophobic moieties (compound 1–5 that contain propyl, butyl, *tert*-butyl, hexyl and ethylhexyl groups), aromatic groups (compound 6–9, containing benzyl, phenyl, *p*-tolyl, 4-ethylphenyl groups), UV-active groups (compound 10–12, bearing 4-fluorophenyl, naphthyl and nitrophenyl groups), or reactive allyl (13) and furfuryl (14) and thiolactone (15) moieties, potentially interesting for the use of the derived oligoamides as cross-linkers or chain extenders.

Furthermore, protecting groups that can be deprotected under orthogonal conditions (acidic or basic) have been introduced (compounds 16 and 17). The complete synthesis procedure and full characterization of this monomer library can be found in the ESI (Schemes S2–S18 and Fig. S13–S29†). Each monomer was tested on the soluble support and their coupling proved to be efficient.

To further demonstrate the robustness of the protocol, a sequence-defined octamer was prepared with the here presented protocol, without further optimization. The aim was to show the possibility to create longer structures with a variety of monomers from the synthesized library (see Fig. S30†). Longer sequence-defined oligomers could also be synthesized if needed, although optimization might be required.

### Investigation of thermal properties

**Fig. 4:**
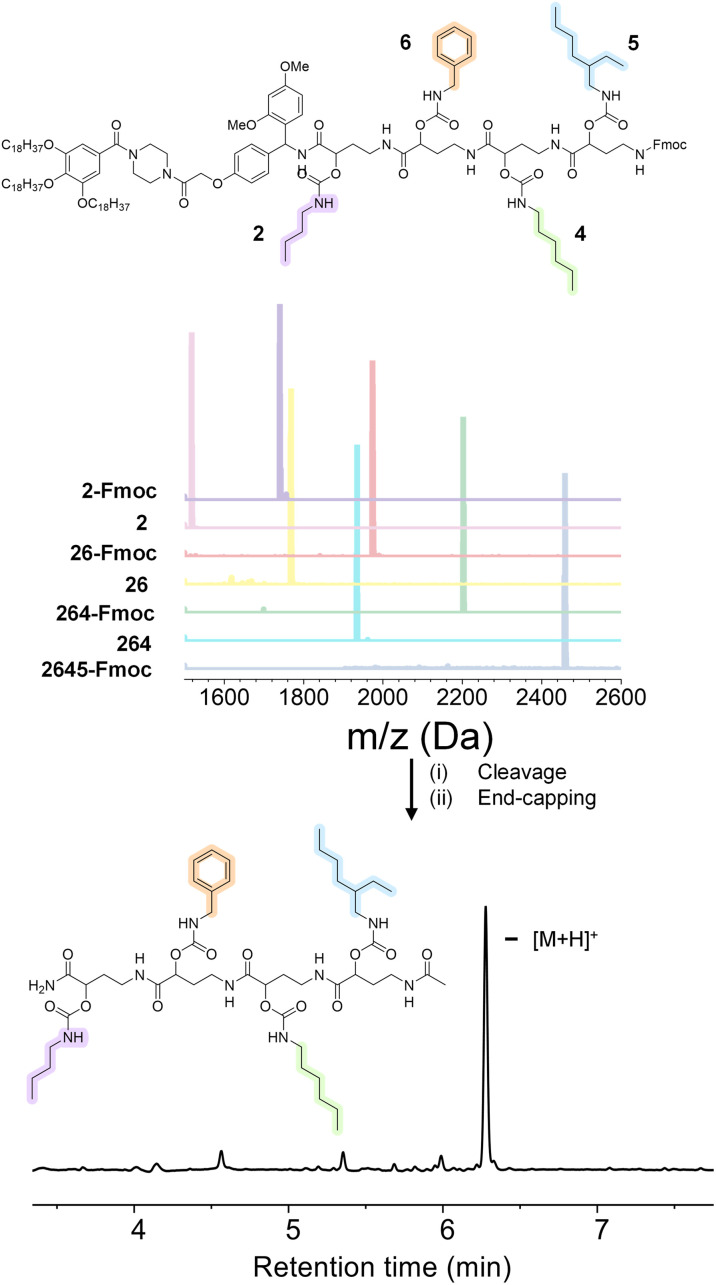
(Top) structure of tetramer A2645 on the RASS and MALDI-ToF evolution of the iterative growth; (bottom) structure of final oligoamide A2645 obtained after cleavage and confirmed by the chromatogram obtained with LC (system peaks at lower retention time).

**Fig. 5:**
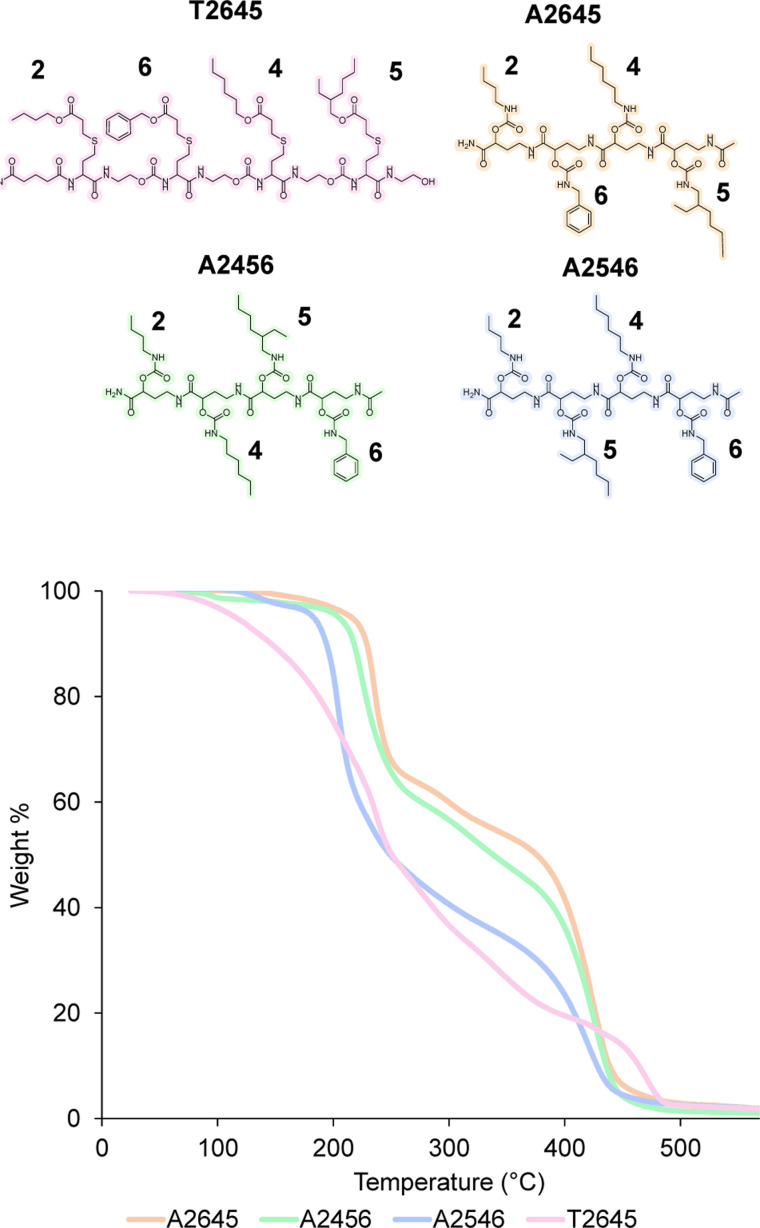
(Top) structure of the tetramers T2645, A2645, A2456 and A2546 compared in this work; (bottom) TGA comparison of the two tetramers at 10 °C min^−1^ heating from 25 °C to 600 °C under air.

In order to prove the robustness of the chemical backbone, a sequence-defined tetramer was synthesized using four of the monomers depicted in Scheme 2 (*i.e.*, monomers 2, 6, 4 and 5 in this arbitrary order). Tetramer A2645 is thus a sequence-defined oligoamide containing a butyl (2), benzyl (6), hexyl (4) and ethyl hexyl (5) side-chain. After the final synthetic step, the N-terminated tetramer was cleaved from the support and the free amine was end-capped with acetic acid to avoid any unwanted side reaction. The iterative growth was followed *via* MALDI-ToF and the structure of the final tetramer was confirmed by LC (Fig. 4). For reasons of comparison of the thermal resistance with another chemical backbone that was previously synthesized *via* thiolactone chemistry, a tetramer analogue T2645 (*i.e.*, bearing similar side-chains) was prepared on a functionalized RASS (see Fig. 5 for complete structure and ESI† for the synthesis protocol and characterization Fig. S31†).^60,61^

The synthesis of the thiolactone oligomer resulted in a lower overall yield (65%, 8 synthetic steps and 8 workups) compared to the oligoamide synthesis (77%, 9 synthetic steps and 5 workups), demonstrating again the advantage of the one-pot two-step strategy of the oligoamide (not possible in the case of the thiolactone protocol). TGA measurements, conducted for both oligomers (Fig. 5), also showed that the thiolactone-based tetramer has a significant lower thermal stability, with an onset degradation around 140 °C *versus* 190 °C for the oligoamide analogue.

The thiolactone degradation profile seems to happen in a multiple-step process as a result of different chemical bonds in the structure: thioether and ester degrade first, followed by urethane and amide bonds. On the other hand, oligoamide-urethane oligomers show a first drop at around 190 °C that can be attributed to the urethane bond, whereas the second drop is ascribed to the more stable amide bond in the backbone.

Moreover, in order to further show the versatility of the protocol and at the same time investigate potential sequence-related thermal stability, two other oligoamide tetramers have been synthesized with the same monomers but in a different order, *i.e.*, A2456 and A2546 (see Fig. S32 and S33† for their characterization). These two oligomers follow a similar degradation pattern (see Fig. 5), but they show slight differences in their onset degradation temperature, which suggests a correlation between the positioning of the monomers in the sequence and the thermal stability of the resulting oligomers. However, a more detailed investigation are would be necessary to confirm and explain this tendency since those structures contain similar covalent bonds.

### Bidirectional growth on uniform soluble support

As mentioned before, the one-pot two-step protocol for the sequence-defined oligoamides is already a substantial improvement in comparison to typical synthetic strategies for such oligoamides. Additionally, we decided to further adapt it towards a bidirectional growth approach, which was used herein to create symmetric and telechelic sequence-defined oligoamides, introducing two reactive moieties in different positions.

**Fig. 6:**
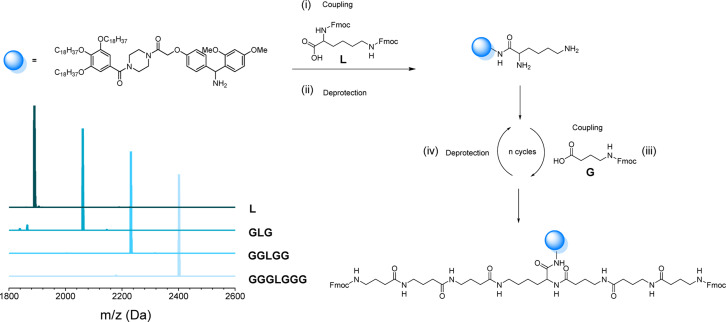
(i) Synthesis of lysine-functionalized RASS, leading to a bisfunctional soluble-support for bidirectional growth synthesis; (ii) coupling and deprotection step with GABA to obtain the symmetrical oligomer GGGLGGG attached on the support; (iii) MALDI-ToF evolution of all coupling steps.

Such structures could be used as potential cross-linker for further investigation in material applications. Additionally, this strategy results in a faster growth of the macromolecule and a higher overall yield can be obtained by halving the number of workups to reach the same molecular weight. In fact, if compared to the synthesis described in the previous paragraph, the tetramer A2645 (Fig. 4) required 9 synthetic steps and 5 workups (overall yield of 77%), whereas with the bidirectional growth protocol, a heptamer (Fig. 6) could be obtained with the same number of synthetic steps and workups, with similar overall yield (80%). When comparing longer sequence-defined macromolecules, increased overall yields are even expected.

**Fig. 7:**
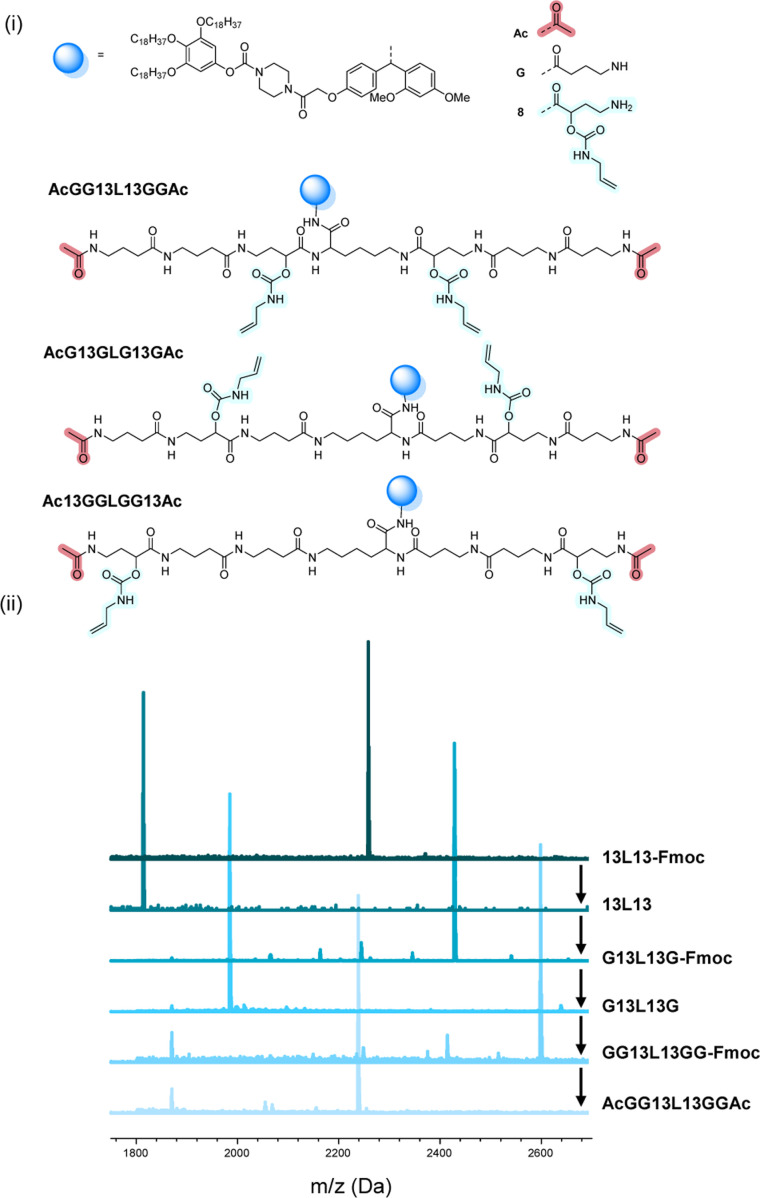
(i) Complete structure of the three heptamers AcGG13L13GGAc, AcG13GLG13GAc and Ac13GGLGG13Ac synthesized on RASS. (ii) Example of the MALDI-ToF evolution for the first heptamer.

The scale at which the experiments were conducted has been ranging from 1 g (oligomers on support) to 100 mg (cleaved oligomers), but the protocol could be upscaled to multigram synthesis as already demonstrated.^25^ In order to perform the bidirectional growth synthesis, a suitable bifunctional molecule has to be introduced at the start of the synthesis. For that reason, the natural amino acid lysine, which contains two primary amino-groups, was selected and successfully introduced as a first monomer, as evidenced *via* MALDI-ToF (Fig. 6 and S34†). As a proof of concept, a sequence-defined heptamer (GGGLGGG) was synthesized using only GABA during the sequential growth (Fig. 6). To further show the versatility of this approach, allylic moieties have been introduced in the telechelic sequence-defined oligomers, respectively as a side-chain or as an end-group functionality. For the introduction of the allyl moiety as a side-chain, the allyl-GABA monomer (13, Scheme 2) has been used. Three different acetic acid (Ac) end-capped sequence-defined octamers were targeted with varying positioning of 8 in the sequence, namely Ac13GGLGG13Ac, AcG13GLG13GAc and AcGG13L13GGAc (structures given in Fig. 7(i)). The evolution of the sequential growth was followed by MALDI-ToF (Fig. 7(i)) for AcGG13L13GGAc and the cleavage proved to be successful (Fig. S35–S37† for two other oligomers). Those oligoamides possess urethane bonds that connect the allyl moiety to the side-chain.

**Fig. 8:**
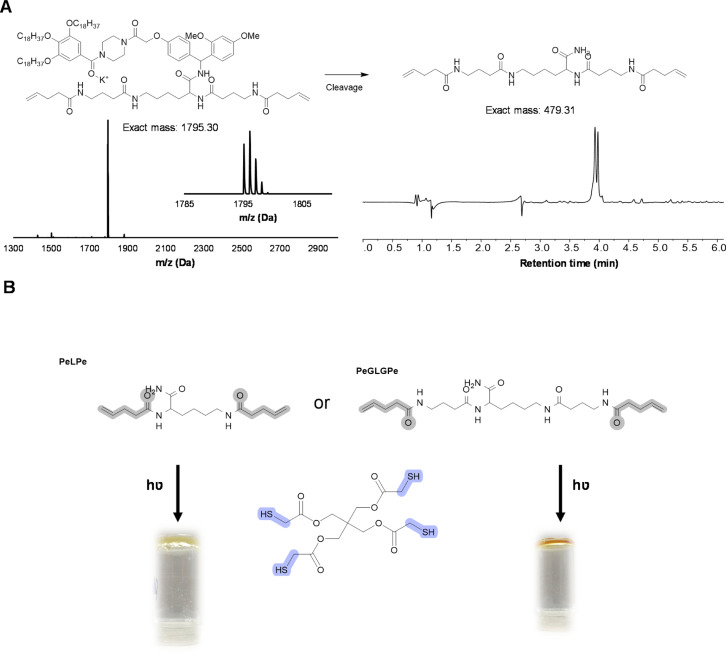
(A) PeGLGPe pentamer on RASS analyzed with MALDI-ToF and cleaved product analyzed with LC-MS (system peaks in the background); (B) network formation with the tetrathiol (2 eq.), oligoamide PeLPe or PeGLGPe (1 eq.), DMPA (0.1 eq.), 1 h, 365 cm^−1^ and vial inversion test.

As an even more stable backbone might be desirable for material applications, an alternative approach has also been investigated. For this, 4-pentenoic acid (Pe) is used to end-cap the macromolecule after cleavage, which introduces the reactive double bonds at the chain ends (Fig. 8). In order to investigate this approach, a trimer (PeLPe) and pentamer (PeGLGPe) have been synthesized (see Fig. 8 and S38, S39†). After obtaining these two diallyl terminated oligoamides, the possibility to cross-link them using a radical thiol–ene reaction was tested. For this, both molecules were reacted separately with a stoichiometric amount of a commercially available tetrathiol, pentaerythritol tetrakis(2-mercaptoacetate), as cross-linker under photoirradiation. Since both telechelic oligoamides are solids, they were first dissolved in methanol, followed by the addition of the tetrathiol and DMPA, prior to irradiation. After 1 h of curing, gel formation could be observed in both cases, indicating network formation. The curing of the material was further verified *via* FT-IR (Fig. S40 and S41†), showing the disappearance of the weak S–H stretch present in the tetrathiol. Moreover, the C=C stretching peak of the allyl group of the oligomers at 1660 cm^−1^ decreased significantly in the resulting network. TGA-analysis, conducted on both the cross-linker PeLPe and its derived network, showed a significant increase of the onset degradation temperature for the networks (from 135 to 250 °C; Fig. S42†). This effect is ascribed to the end-capping of the terminal amines, which inhibits the previously described process of depolymerization. A similar result has been also obtained with the crosslinker PeGLGPe (Fig. S43†). Thermal properties of the two resulting networks were also investigated *via* differential scanning calorimetry. As expected from the increased flexibility, the network based on the longer oligoamide PeGLGPe exhibits a lower glass transition temperature (*T*_g_) with a *T*_g_-value of 10 °C compared to the one with the short PeLPe (*T*_g_ = 21 °C) (Fig. S44†). As a result of this proof-of-concept material study, we envision that this oligoamide platform will offer a straightforward and universal solution for the synthesis of a whole range of sequence-defined molecules to be implemented in polymer networks for exploring in-depth structure–property relations in upcoming studies. In fact, the protocol offers the possibility to introduce different unnatural amino acids in a scalable way, varying the length of the backbone, spacing between amide bonds or adding rigidity in the structure. In this context, it should also be emphasized that the radical thiol–ene reaction only represents a primary example of different chemistries that could be used for network formation, since the versatility of both monomers and protocol allows for the introduction of a plethora of different reactive groups.

## Conclusions

A uniform soluble support has been used for the synthesis of sequence-defined oligoamides by means of Fmoc-chemistry, an approach that is commonly performed on a solid support. This method offered several advantages, including faster coupling kinetics and the ability to perform coupling and deprotection in a two-step, one-pot protocol. Moreover, the obtained amide backbone was shown to be more thermally stable than a similar amide backbone obtained *via* a thiolactone-based protocol, thus showing its potential for use in bulk material science. Additionally, the applied protocol allowed the choice of the building blocks constituting the backbone, giving the possibility to tune the ring-closing depolymerization into 5-membered cyclic structures for eventual recycling of 2-pyrrolidinone. A library of monomers could be synthesized starting from readily available building blocks, showing the possibility to introduce varying functionalities in the side-chains according to the desired properties and application. The lysine attached soluble support also allowed for a bidirectional growth of the sequence, which halved the reaction steps and provided a symmetrical synthesis approach for telechelic sequence-defined oligoamides. Different telechelic discrete structures (trimers, pentamers and heptamers) have been synthesized to show the possibility to vary the backbone and the positioning of a reactive allylic moiety, and to demonstrate the cross-linking ability of two oligomers *via* a UV-initiated thiol–ene process.

## Data availability

The authors declare that all supporting data are available in the ESI† and from the corresponding author upon request.

## Author contributions

This manuscript has been prepared through the contribution of all authors.

## Conflicts of interest

There is no conflict to declare.

## Supplementary Material

SC-015-D3SC04820A-s001
