# Supplementary material for: Telechelic sequence-defined oligoamides: their step-economical synthesis, depolymerization and use in polymer networks
Source: Chem Sci. 2024 Jan 15;15(8):2805–16. doi: 10.1039/d3sc04820a (PMC10882489; doi:10.1039/d3sc04820a)
Supplement: SC-015-D3SC04820A-s001 [file SC-015-D3SC04820A-s001.pdf]

Electronic Supplementary Material (ESI) for Chemical Science.
This journal is © The Royal Society of Chemistry 2024

Supporting information

Telechelic Sequence-Defined Oligoamides: their Step-economical Synthesis, Depolymerization and Use in Polymer Networks

Irene De Franceschi, Nezha Badi and Filip E. Du Prez**

Polymer Chemistry Research group, Centre of Macromolecular Chemistry (CMaC), Department of Organic and Macromolecular Chemistry, Faculty of Sciences, Ghent University, Krijgslaan 281 S4-bis, 9000 Ghent, Belgium

**Corresponding authors: Nezha.Badi@Ugent.be; Filip.Duprez@Ugent.be*

Table of content

Materials 3
Instrumentation 3
Liquid chromatography-mass spectrometry (LC-MS) 3
Nuclear magnetic resonance (NMR) spectroscopy 3
Matrix-assisted laser desorption/ionization – Time of flight (MALDI-ToF) 3
Thermogravimetric analysis (TGA) 4
Differential scanning calorimetry (DSC) 4
Online ATR FT-IR 4
Synthetic procedures 4
A. Synthesis or Rink amide soluble support (RASS) and optimization of the protocol 4
Two-step two-pot strategy for sequence-defined oligomers 5
Two-step one-pot strategy for sequence-defined oligomers 5
Coupling step on RASS with Fmoc-GABA 6
Fmoc deprotection of RASS-functionalized GABA 7
B. Solid-phase synthesis of the trimer GGG 9
GGG-Fmoc trimer prepared via solid-phase synthesis 10
C. Ring-closing depolymerization of trimers GGG, GBG and GGB 11
Synthesis of GGG, GBG and GGB trimers on RASS and cleavage 11
Depolymerization of trimer GGB and GBG 14
D. Synthesis of the different monomers 15
Fmoc protection of (*S*)-(−)-4-amino-2-hydroxybutyric acid 15
Synthesis of (1): 4-Fmoc(amino)-2-((propylcarbamoyl)oxy)butanoic acid 17
Synthesis of (2): 4-Fmoc(amino)-2-((butylcarbamoyl)oxy)butanoic acid 19
Synthesis of (3): 4-Fmoc(amino)-2-((tertbutylcarbamoyl)oxy)butanoic acid 21
Synthesis of (4): 4-Fmoc(amino)-2-((hexylcarbamoyl)oxy)butanoic acid 23
Synthesis of (5): 4-Fmoc(amino)-2-((ethylhexylcarbamoyl)oxy)butanoic acid 25

Synthesis of (6): 4-Fmoc(amino)-2-((benzylcarbamoyl)oxy)butanoic acid 27
Synthesis of (7): 4-Fmoc(amino)-2-((phenylcarbamoyl)oxy)butanoic acid 29
Synthesis of (8): 4-Fmoc(amino)-2-(*p*-tolylcarbamoyloxy)butanoic acid 31
Synthesis of (9): 4-Fmoc(amino)-2-(4-ethylphenylcarbamoyloxy)butanoic acid 33
Synthesis of (10): 4-Fmoc(amino)-2-(4-fluorophenylcarbamoyloxy)butanoic acid 35
Synthesis of (11): 4-Fmoc(amino)-2-(4-nitrobenzoyloxy)butanoic acid 37
Synthesis of (12): 4-Fmoc(amino)-2-((napthoyl)oxy)butanoic acid 39
Synthesis of (13): 4-Fmoc(amino)-2-((allylcarbamoyl)oxy)butanoic acid 41
Synthesis of (14): (R)-4-Fmoc(amino)-2-((furfurylcarbamoyl)oxy)butanoic acid 43
Synthesis of (15): 4-Fmoc(amino)-2-((thiolactonecarbamoyl)oxy)butanoic acid 45
Synthesis of (16): 4-Fmoc(amino)-2-TBDMS(hydroxy)butanoic acid 47
Synthesis of (17): 4-Fmoc(amino)-2-THP(hydroxy)butanoic acid 49
E. Synthesis of amide sequence-defined octamer on RASS 51
F. Synthesis of thiolactone-based sequence-defined tetramer on RASS 52
Tla-Rink amide soluble support 52
Synthesis of sequence-defined thiolactone tetramer 53
G. Synthesis of sequence-defined tetramers A2456 and A2546 55
H. Synthesis of bidirectional RASS 57
I. Synthesis of telechelic sequence-defined oligoamide-urethane heptamers 58
J. Synthesis of telechelic sequence-defined oligoamides for network formation 60
K. General procedure for network formation 62
L. Characterization of the networks 62

Materials

All chemicals were used as supplied, unless otherwise stated. Deionized water was used in the procedures. Deuterated methanol (MeOD), DMSO-d6 and $CDCl_3$ were purchased from Eurisotop. Magnesium sulfate (dried ≥ 99%), sodium carbonate (99%) and potassium carbonate (99%) were purchased from Carl Roth. Hydrochloric acid (36 wt%) and acetic acid (96%) were purchased from Chem Lab NV. Trifluoroacetic acid (peptide grade), Fmoc-protected Rink-amide resin (100-200 mesh, 1 % DVB, 0.78 mmol g^{-1}) and Fmoc-Lys(Fmoc)-OH (≥ 98 %) were purchased from Iris Biotech GmbH. (S)-4-Amino-2-hydroxybutyric acid and hexafluorophosphate azabenzotriazole tetramethyl uronium (HATU) were purchased from FluoroChem Ltd. 4-(Aminomethyl)benzoic acid (97%) was purchased from Carbosynth and Fmoc-Rink amide linker (97%) was purchased from Aapptec. N,N'-Diisopropylcarbodiimide (DIC), 1-ethyl-3-(3-dimethylaminopropyl)-carbodiimide (EDC) and 1-(tert-butoxycarbonyl)piperazine (98%) were purchased from abcr GmbH. Chloroform (HPLC grade, 99%), methanol (HPLC grade, 99.5%), tetrahydrofuran (HPLC grade, 99.9%) and triethylamine (99%) were purchased from Acros Organics. Diethyl ether (HPLC grade, 99%) and N,N-dimethylformamide (HPLC grade, 99.7%) were purchased from Fischer Scientific. Butyl isocyanate (98%), hexyl isocyanate (97%), 2-ethylhexyl isocyanate (98%), phenyl isocyanate (≥98%), benzyl isocyanate (99%), 1-naphthyl isocyanate (98%), 4-nitrophenyl isocyanate (97%), allyl isocyanate (98%), furfuryl isocyanate (97%), p-tolyl isocyanate (97%), 4-fluorophenyl isocyanate (98 %), 4-ethyl phenyl isocyanate (98 %), propyl isocyanate (95 %), tertbutyl (97 %), were all purchased from Sigma Aldrich. Tert-butyldimethylsilyl chloride (TBDMSCl), 3,4-dihydropyran (DHP), zirconium (IV) acetylacetonate (97 %), 1-bromo octadecane (97%), 9-fluorenylmethyl chloroformate (Fmoc-Cl, 97%), triisopropylsilane (TIPS, 98%), piperidine (99%), 4-pentenoic acid (97%) and 4-dimethylaminopyridine (99%) were also purchased from Sigma Aldrich. Butyl acrylate (99%), phenyl acrylate (98%), hexyl acrylate (96 %), 2-ethylhexyl acrylate (98%), ethanolamine (99%), methyl gallate (98%), 1,8-Diazabicyclo[5.4.0]undec-7-ene (98%), piperazine anhydrous (98%) and pentaerythritol tetrakis(2-mercaptoacetate) (95%) were purchased from TCI Europe.

Instrumentation

Liquid chromatography-mass spectrometry (LC-MS)

Liquid chromatography mass spectrometry (LC-MS). LC-MS spectra were recorded on an Agilent technologies 1100 series LC/MSD system equipped with a diode array detector and single quad MS detector (VL) with an electrospray source (ESI-MS) for classic reversed phase LC-MS and MS analysis. All results were recorded in positive mode unless otherwise stated. Analytic reversed phase HPLC (high-performance liquid chromatography) was performed with a Phenomenex C18 (2) column (5 μ, 250 × 4.6 mm) using a solvent gradient (0 → 100 % acetonitrile in H_2O in 10 min) and the eluting compounds were detected via UV-detection (λ = 214 nm).

Nuclear magnetic resonance (NMR) spectroscopy

^1H spectra were recorded on a Bruker Avance 300 (300 MHz) and a Bruker Avance 400 (400 MHz). $CDCl_3$, DMSO-d_6 or MeOD were used as solvents. Chemical shifts are presented in parts per million (δ) and calibrated to the characteristic residual solvent signals.

Matrix-assisted laser desorption/ionization – Time of flight (MALDI-ToF)

The MALDI-ToF measurements were conducted on an Applied Biosystems Sciex 4800+ MALDI TOF/TOF analyzer, controlled by 4000 Series Explorer software (Applied Biosystems, Germany). Stock solutions of the matrix, dithranol (45 mg/mL) or trans-2-[3-(4-tert-butylphenyl)-2-methyl-2-propenylidene]malonitrile (DCTB, 45 mg/mL) and sodium trifluoroacetate (NaTFA, 15 mg/mL) were solubilized in tetrahydrofuran.

Oligomeric samples were also prepared in tetrahydrofuran (15 mg/mL). To spot the MALDI plate, 45 µL of the matrix stock solution was mixed with 15 µL of the stock solutions of each, the salt and the sample. The spots were dried and then the plate was loaded into the instrument. The measurements were performed in reflective positive ion mode.

Thermogravimetric analysis (TGA)

Thermogravimetric analyses (TGA) were performed with a Mettler Toledo TGA/ SDTA851e instrument under nitrogen atmosphere at a heating rate of 10 $K.min^{-1}$ from 25 °C to 800 °C in dynamic mode.

Differential scanning calorimetry (DSC)

Differential scanning calorimetry (DSC) analyses were performed with a Mettler Toledo instrument 1/700 under nitrogen atmosphere at a heating and cooling rate of 10 $K.min^{-1}$. Measurements were performed from -50 to 150 °C.

Online ATR FT-IR

Time-resolved online ATR FT-IR spectra were recorded on a React-IR 4000 Instrument (Mettler Toledo AutoChem ReactIR) equipped with a silicon ATR probe (SiComp, optical range 4400–650 cm^{-1}). For online monitoring, the silicon probe was introduced into a one neck flask containing the reaction mixture and spectra were recorded every minute. The solvent spectrum was recorded at the reaction temperature and subtracted to enhance the signal of the reaction species.

Synthetic procedures

A. Synthesis or Rink amide soluble support (RASS) and optimization of the protocol

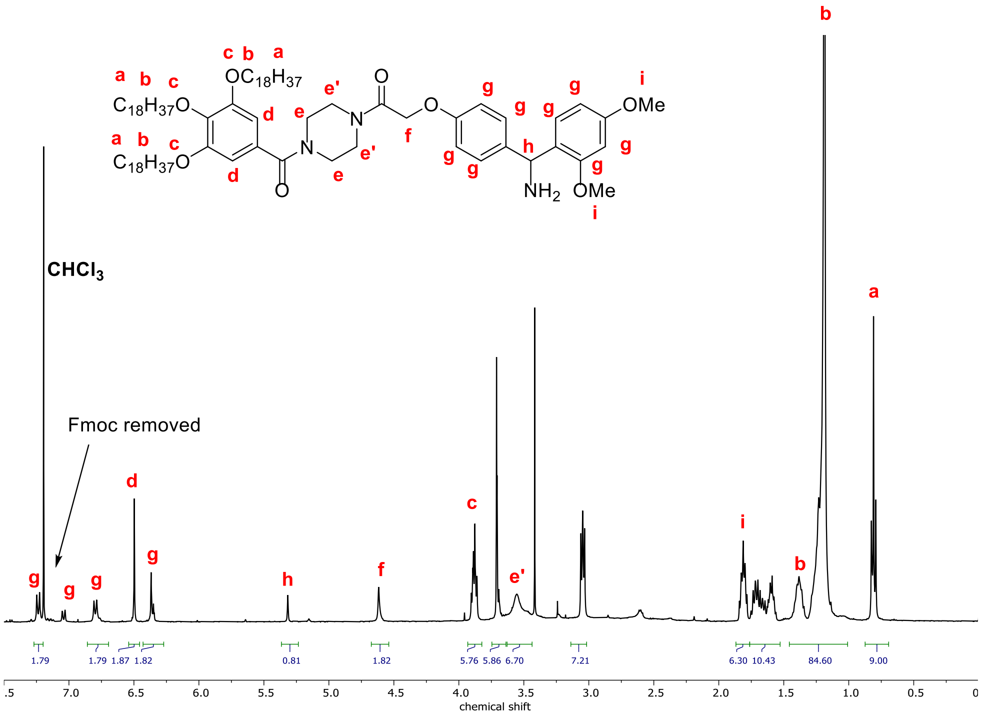

Figure S1: Structure and ^{1}H-NMR (400 MHz in $CDCl_3$) of Rink amide soluble support.

For the complete synthesis of the Rink amide soluble support, the reader is referred to the supporting information of the paper at the link **https://doi.org/10.1039/D2PY00883A**.[1]

Two-step two-pot strategy for sequence-defined oligomers

General procedure for the coupling of amino acids

RASS is dissolved in $CHCl_3$ (1 mg/mL) at room temperature under vigorous stirring. Then 0.05 eq of DMAP and 1.2 eq. of the selected amino acid are added, followed by a dropwise addition of 1.2 eq. of DIC dissolved in $CHCl_3$. The reaction is monitored via MALDI-ToF. When the peak of the starting compound is totally consumed (coupling time 10-70 mins), the reaction is concentrated and precipitated in cold methanol. The white powder is recovered via filtration on Millipore filters, washed with additional cold methanol and dried in a vacuum oven at 40 °C.

General procedure for the Fmoc-deprotection of amino acids

The functionalized RASS-amino acid-Fmoc is dissolved in $CHCl_3$ (1 mg/mL) at room temperature under vigorous stirring. Then 5 wt% of piperazine is added to the mixture and the system is left stirring at room temperature for 5 min. Afterwards 1 wt% of DBU is added and the reaction mixture is left stirring for additional 5-10 min. The reaction is monitored via MALDI-ToF. When the Fmoc deprotected is complete, the reaction medium is concentrated and precipitated in cold methanol. The white powder is recovered via filtration on Millipore filters, washed with additional cold methanol and dried in a vacuum oven at 40 °C.

Two-step one-pot strategy for sequence-defined oligomers

General procedure for one-pot coupling and deprotection of amino acids

RASS is dissolved in $CHCl_3$ (1 mg/mL) at room temperature under vigorous stirring. Then 0.05 eq. of DMAP and 1.2 eq. of amino acid are added, followed by a dropwise addition of 1.2 eq. of DIC dissolved in $CHCl_3$. The reaction is monitored via MALDI-ToF. When the peak of the starting compound is totally consumed (coupling time 10-70 min), 5 eq. of water is added under stirring to quench the DIC. After 10 min, 5 w% of piperazine is added in the reaction mixture, followed by 1 wt% of DBU. The deprotection is monitored again via MALDI-ToF (deprotection time: 5-10 min). When the Fmoc deprotection is complete, the reaction is concentrated and precipitated in cold methanol. The white powder is recovered via filtration on Millipore filters, washed with additional cold methanol and dried in a vacuum oven at 40 °C.

General procedure for the bidirectional growth synthesis of sequence-defined oligomers

RASS-lysine is dissolved in $CHCl_3$ (1 mg/mL) at room temperature under vigorous stirring. Then 0.05 eq of DMAP and 2.5 eq. of amino acid are added, followed by a dropwise addition of 2.5 eq. of DIC dissolved in $CHCl_3$. The reaction is monitored via MALDI-ToF. When the peak of the starting compound is totally consumed (coupling time 10-70 min), 5 eq. of water is added under stirring to quench the DIC. After 10 min, 5 w% of piperazine is added in the reaction mixture, followed by 1 wt% of DBU. The deprotection is monitored again via MALDI-ToF (deprotection time 5-10 min). Once the Fmoc deprotection is complete, the reaction is concentrated and precipitated in cold methanol. The white powder is recovered via filtration on Millipore filters, washed with additional cold methanol and dried in a vacuum oven at 40 °C.

General procedure for cleavage of the sequence-defined oligomer from RASS

RASS-sequence-defined oligomer is dissolved in CH_2Cl_2 (1 mg/mL), then 2.5 wt% of TIPS and 47.5 wt% of TFA are added to the solution. After 2 h, the solution is concentrated and precipitated in cold methanol. The liquid is separated from the solid, and the solvent evaporated until complete dryness. Afterwards, ice-cold diethyl ether is added. The solution is centrifuged, and the cleaved product is collected in the form of a white precipitate, which is subsequently dried in a vacuum oven at 40 °C.

Coupling step on RASS with Fmoc-GABA

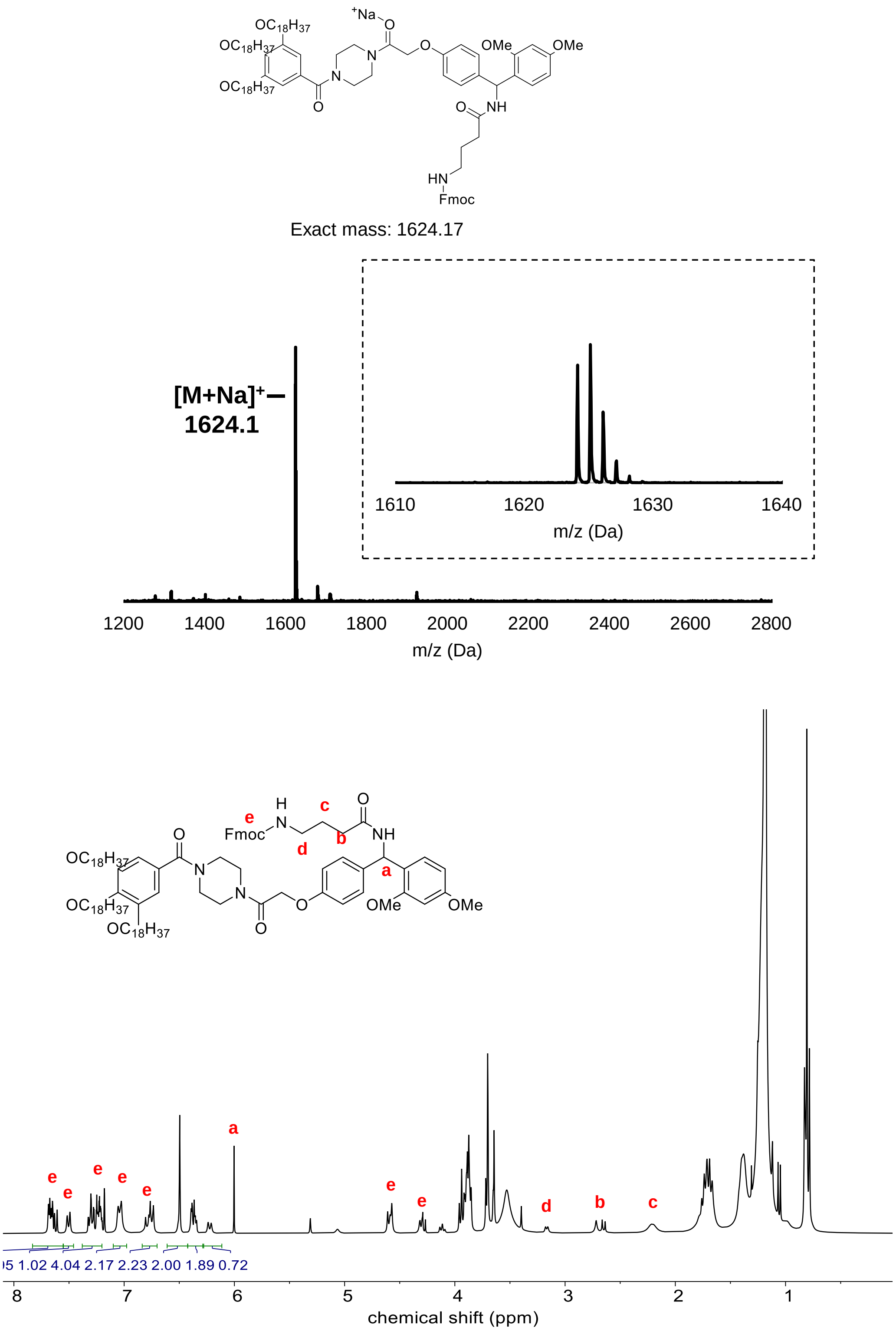

Figure S2: Structure, MALDI-ToF and ^{1}H-NMR spectra of the Rink amide soluble support functionalized with Fmoc-GABA.

Fmoc deprotection of RASS-functionalized GABA

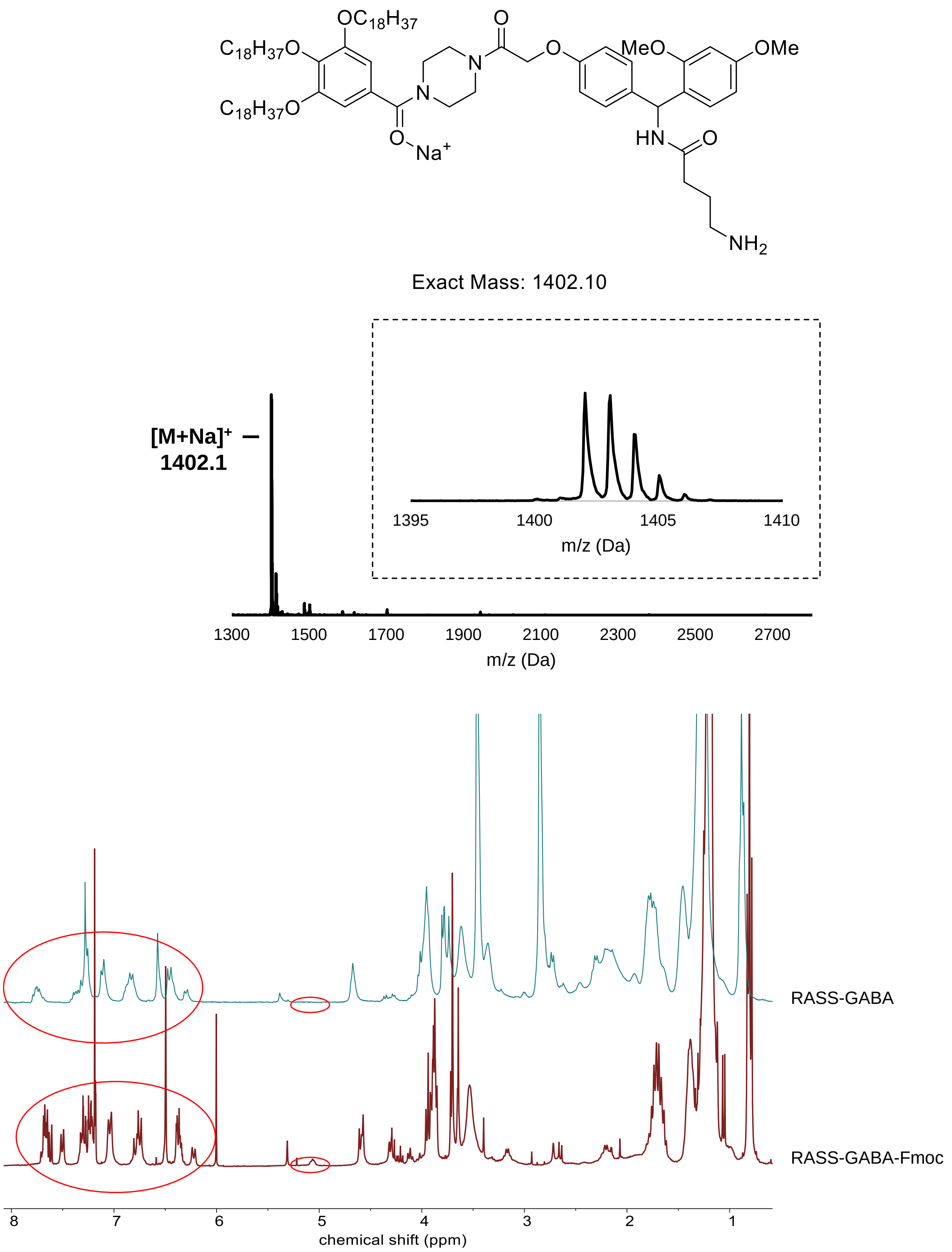

Figure S3: (Top) Structure and MALDI-ToF analysis of the obtained Rink amide soluble support functionalized with GABA after Fmoc deprotection. ^{1}H-NMR in $CDCl_3$ comparison of the compound before and after deprotection.

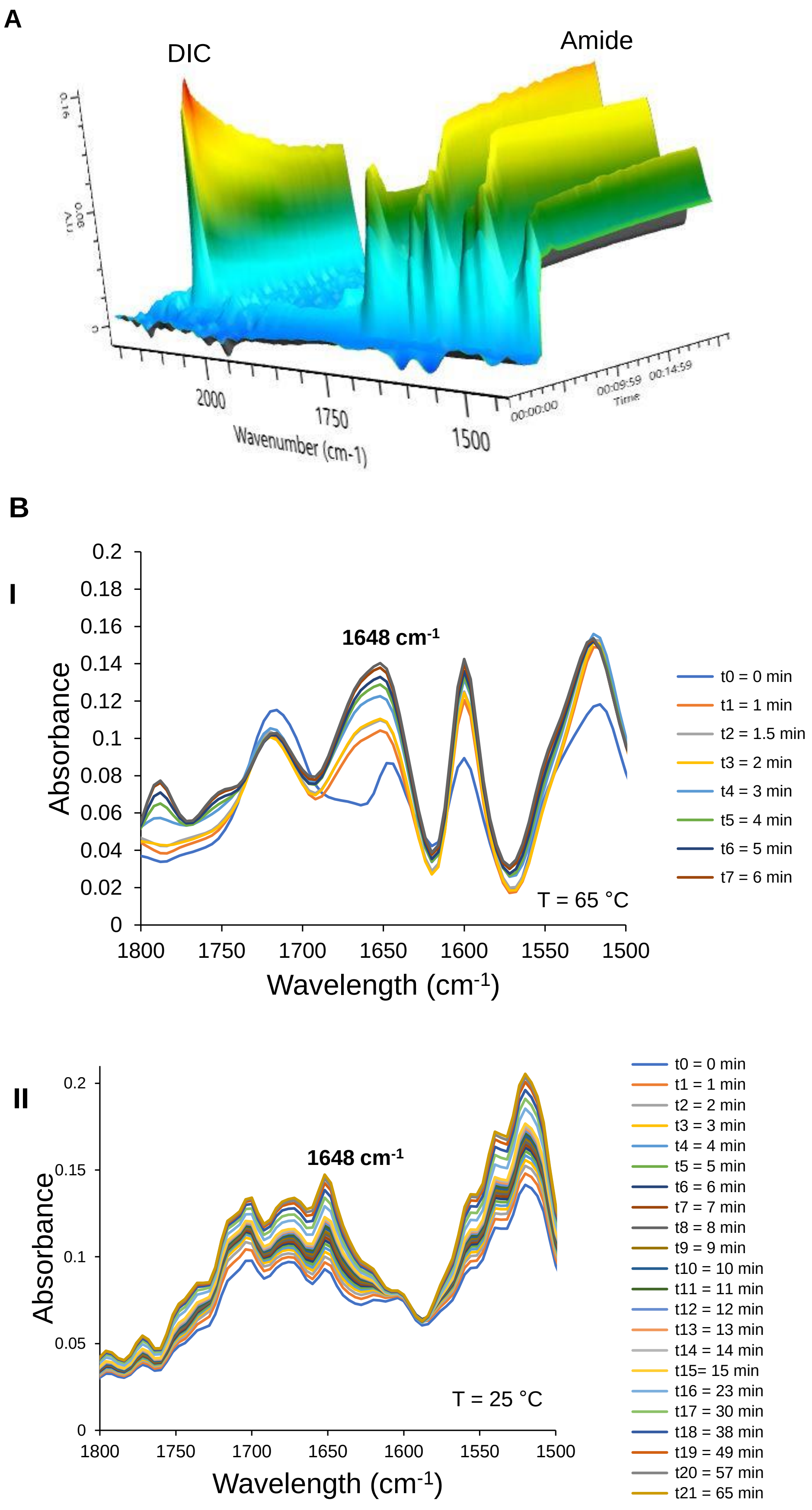

Figure S4: A) Online IR for following the consumption of DIC (2000 cm^{-1}) and formation of the amide bond (1648 cm^{-1}). B) IR evolution showing amide bond formation at 65 °C (I) and 25 °C (II).

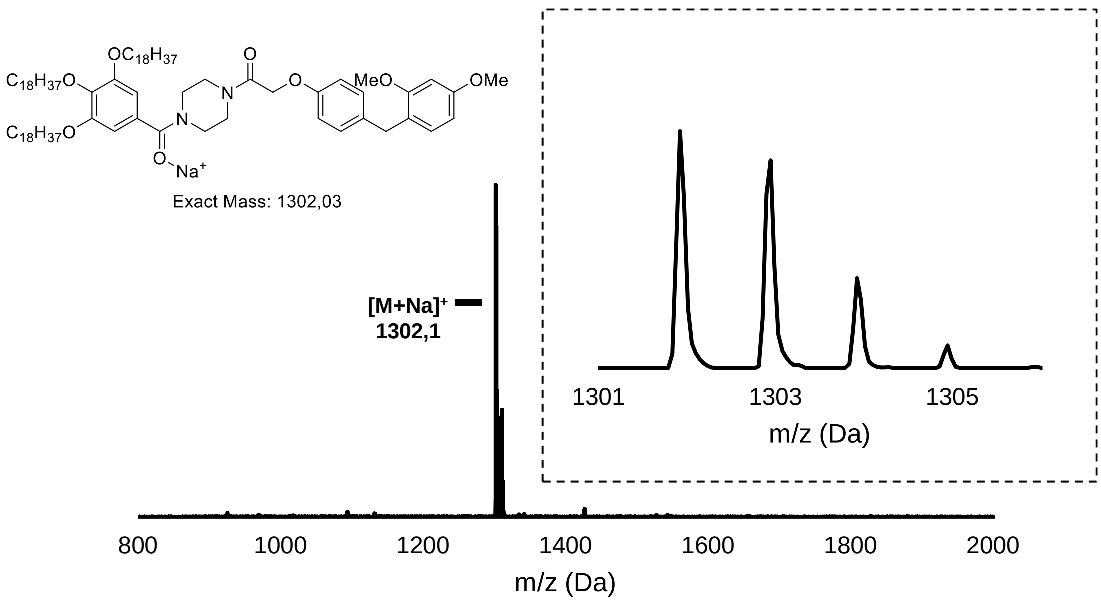

Figure S5: Structure of the RASS after cleavage and corresponding MALDI-ToF spectrum.

B. Solid-phase synthesis of the trimer GGG

Deprotection of the resin

A commercially available Fmoc-protected Rink-amide resin (300 mg, loading 0.78 mmol/g) was swollen in DMF (3 mL) for 10 min. Then the solvent was removed, and the resin washed with DMF (3x5 mL). After the addition of the piperidine solution (20 vol% in DMF, 3 mL), the reaction mixture was shaken vigorously for 1 h. The reaction solution was removed, and the resin was washed with DMF (3x5 mL) and $CHCl_3$ (3x5 mL). After the successful removal of the Fmoc group, the resin was swollen in $CHCl_3$ (3 mL) under inert atmosphere for 10 min.

General procedure for coupling of the amino acid

The solid support was swollen for 5 min in dry DMF (0.5 mL per 100 mg of resin). A solution of HATU (0.3 mmol, 4 eq.), GABA (0.3 mmol, 4 eq.) and DIPEA (0.37 mmol, 5 eq.) is prepared in 0.5 mL (total 1 mL) of DMF and added to the swollen support. The reaction mixture was shaken for 30 min. The solution phase was filtered off and washed with DMF (4 times), $CHCl_3$ and methanol.

General procedure for Fmoc deprotection

The solid support was swollen for 10 min in dry DMF (1 mL per 100 mg of resin). Subsequently it was filtered and 1 mL of a solution of 20 % piperidine in DMF was added and the reaction mixture was shaken for 8 min. The solution phase was filtered off and the procedure was repeated a second time to ensure a complete deprotection. Then, the solid phase was washed again with DMF (4 times), methanol (4 times), $CHCl_3$, diethyl ether and dried.

General procedure for the cleavage from resin

The solid support was swollen in a mixture of 10 % TFA, 2 % TIPS in CH_2Cl_2 (1 mL per 100 mg of resin). After 30 min, the liquid was recollected, and the resin was washed with additional 1 mL of CH_2Cl_2 to recover the cleaved sequence-defined oligomer. The liquid part was recovered after filtration, evaporated, and freeze dried to remove completely the TFA.

GGG-Fmoc trimer prepared via solid-phase synthesis

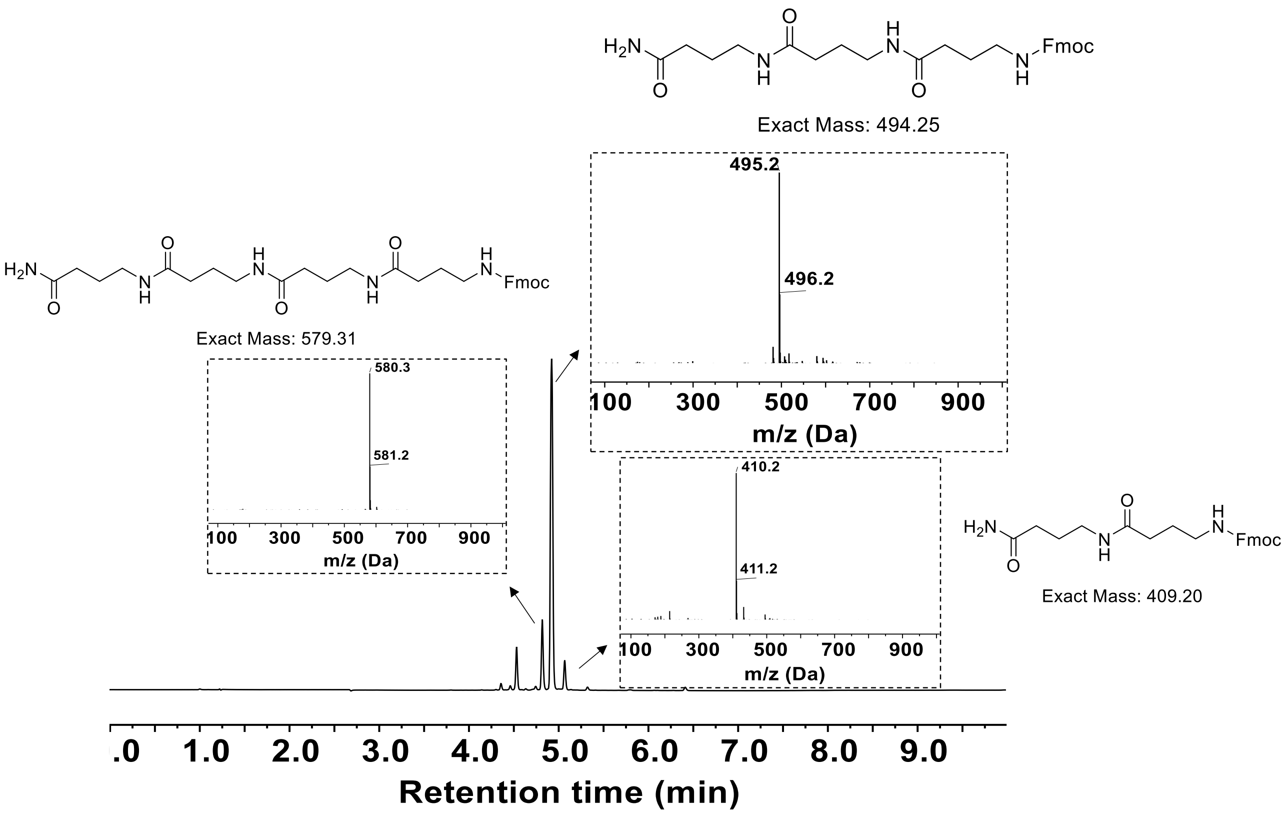

Figure S6: Final LC-MS of trimer GGG-Fmoc prepared using solid-phase synthesis. Note: the impurities identified (dimers and tetramers) are coming from uncomplete couplings and washings.

C. Ring-closing depolymerization of trimers GGG, GBG and GGB

Synthesis of GGG, GBG and GGB trimers on RASS and cleavage

The trimers are synthesized on a RASS after 6 synthetic steps (3 coupling and 3 deprotection), followed by final cleavage from the RASS.

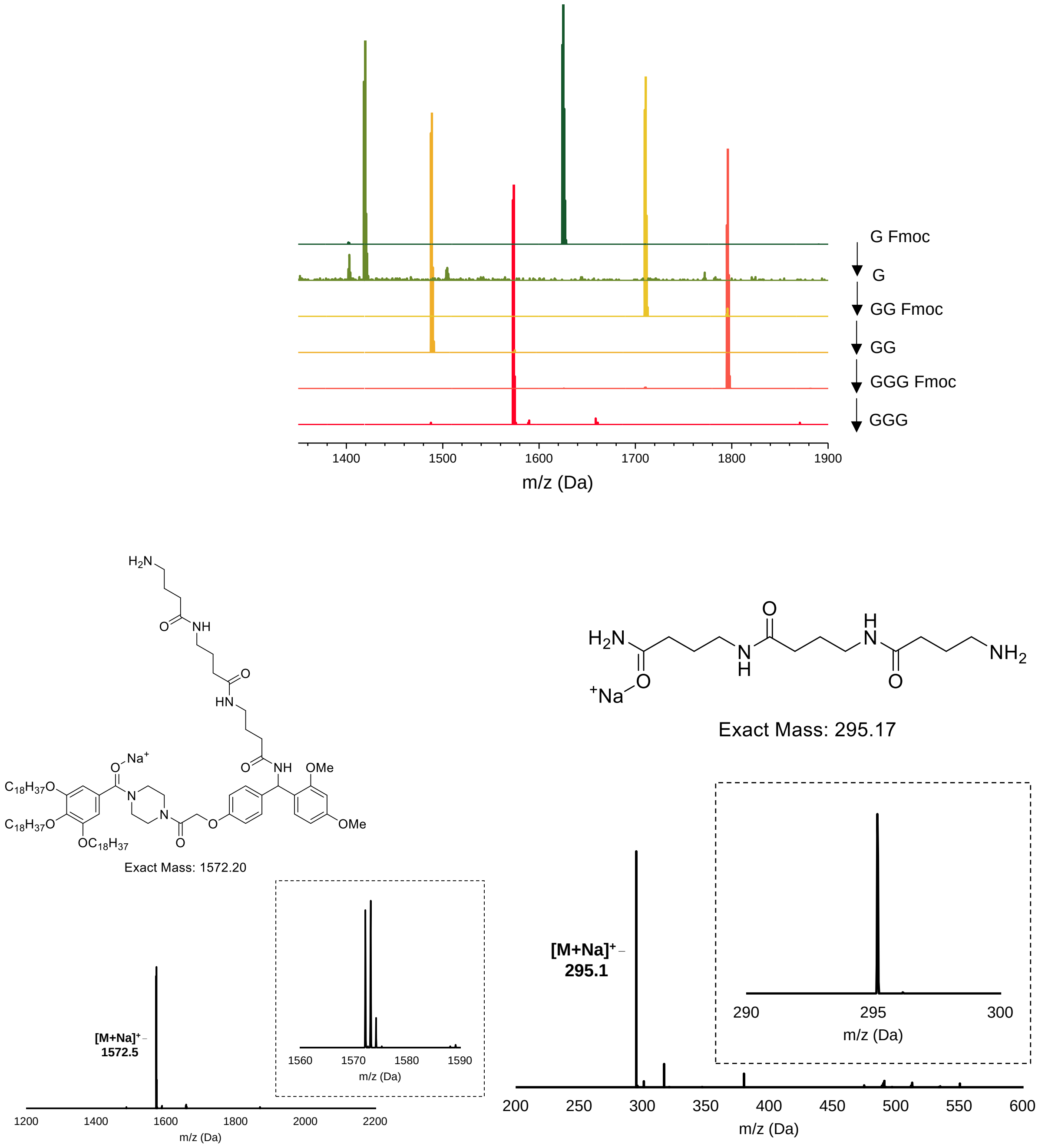

Figure S7: MALDI-ToF evolution of the synthesis of trimer GGG synthesized from the RASS and MALDI-ToF of the cleaved trimer.

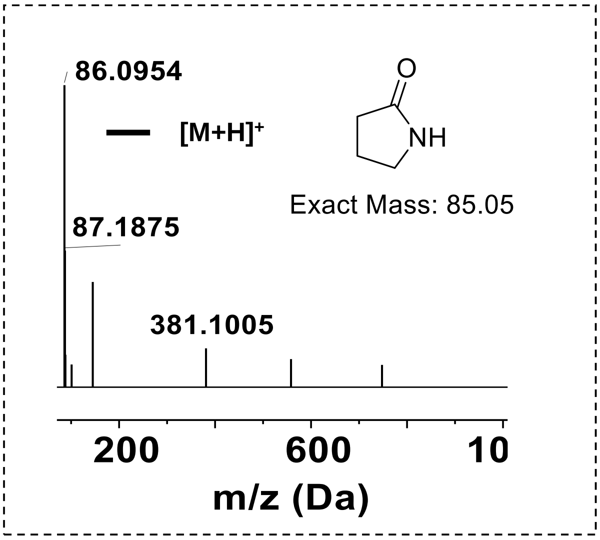

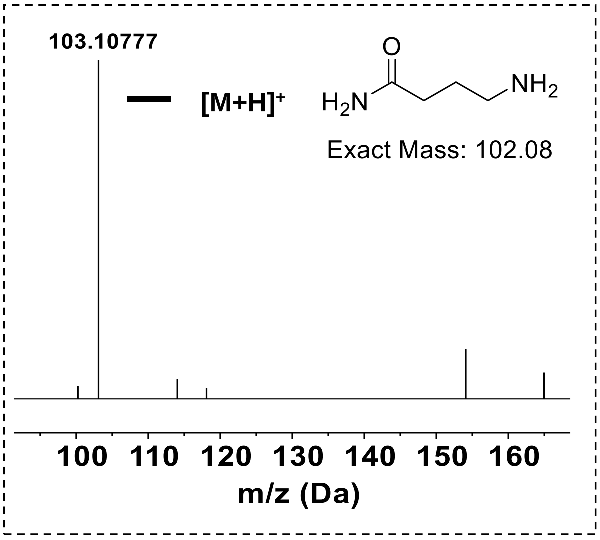

Figure S8: MS spectra from LC-MS measurement of GGG trimer following depolymerization; A) peak at 1.7 min (Gc); B) peak at 2.2 min (G).

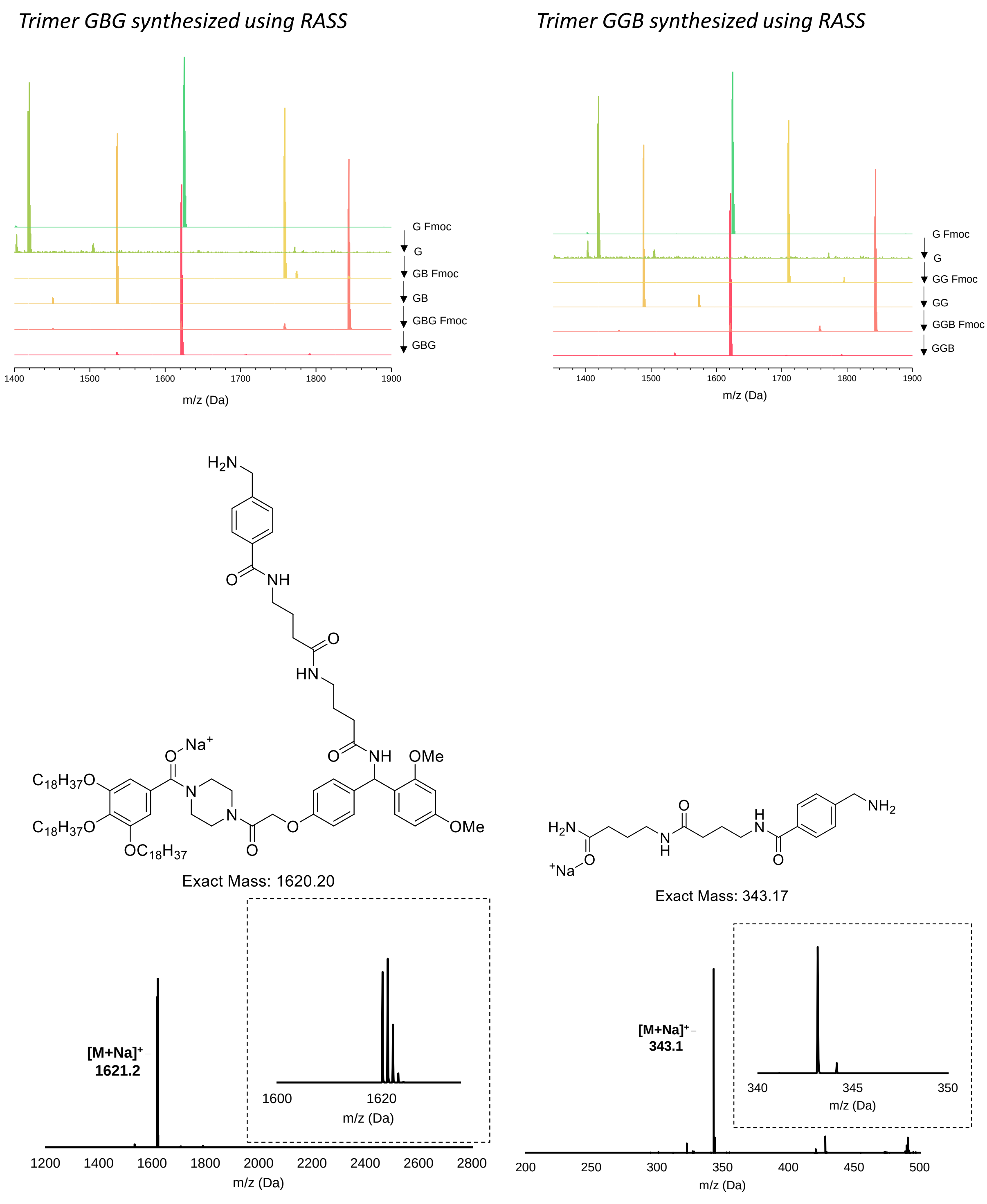

Figure S9: (Top) MALDI-ToF following the synthesis of the trimers GBG and GGB using RASS and (Bottom) MALDI-ToF of the cleaved oligomers.

Depolymerization of trimer GGB and GBG

The trimers (GGB or GBG) are placed in a vial under continuous stirring and dissolved in DMSO-d_6. They are left stirring at 160 °C and samples are collected for ^{1}H-NMR at t_0, t_1 = 30 min, t_2 = 1 h and t_3 = 2 h.

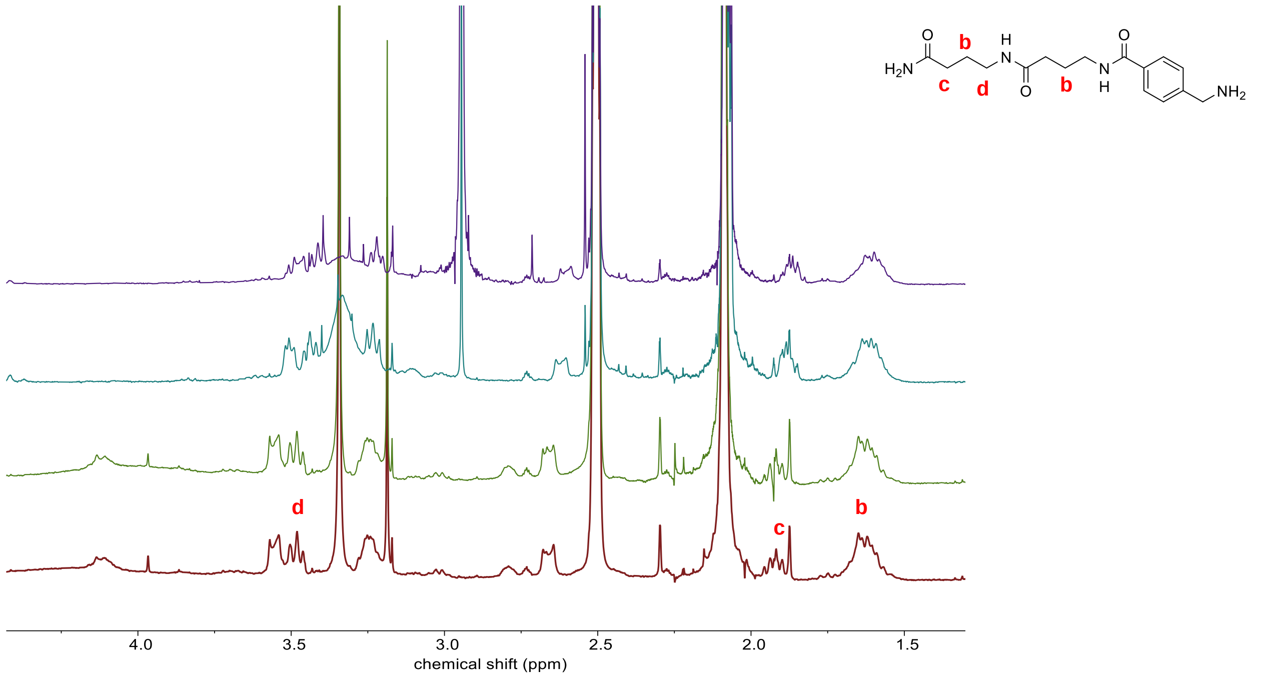

Figure S10: ^{1}H-NMR (400 MHz) of trimer GGB in DMSO-d_6 after stirring at 160 °C for t_0, t_1, t_2 and t_3.

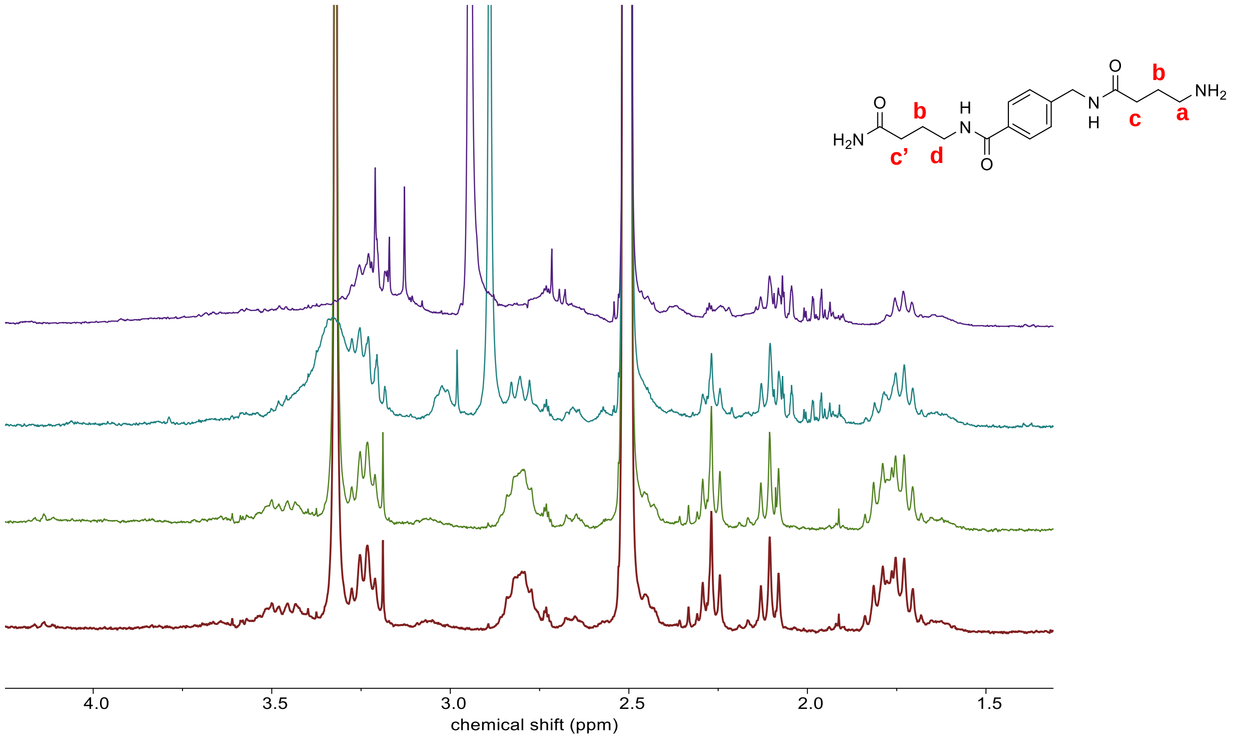

Figure S11: ^{1}H-NMR 400 MHz of trimer GGB in DMSO-d_6 after stirring at 160 °C for t_0, t_1, t_2 and t_3.

D. Synthesis of the different monomers

Fmoc protection of (*S*)-(−)-4-amino-2-hydroxybutyric acid

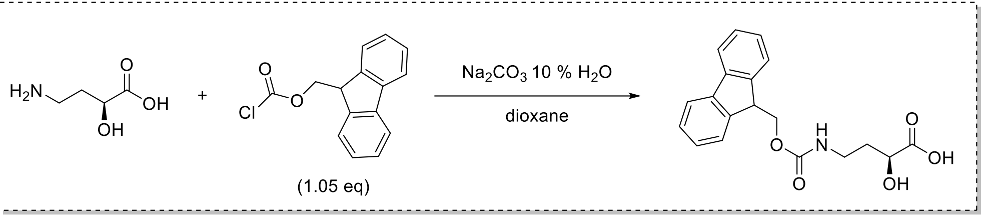

Scheme S1: Fmoc protection of 4-amino-2-hydroxybutanoic acid.

The synthesis was adapted from a literature procedure. 4-Amino-2-hydroxybutanoic acid (7 g, 58.76 mmol, 1 eq.) was dissolved in a solution of dioxane (70 mL) and 10 % Na_2CO_3 (175 mL). The reaction was cooled down to 0°C and a solution of Fmoc-Cl (15.96 g, 61.70 mmol, 1.05 eq) in dioxane (90 mL) was added dropwise in the reaction flask. The mixture was stirred in an ice bath for 1 hour and at room temperature for two hours. The reaction solution was poured into water (500 mL), extracted with diethyl ether (200 mL x2). The aqueous layer was cooled in an ice bath and acidified with 1.0 N HCl to pH 2 until a precipitate was formed, then extracted with EtOAc (200 mL x 3). The collected organic layers were dried over $MgSO_4$ and evaporated. The product was obtained in the form of a white powder. Yield: 18.2 g white powder, 53.32 mmol, 90.73 % yield. MW: 941.61 g/mol.

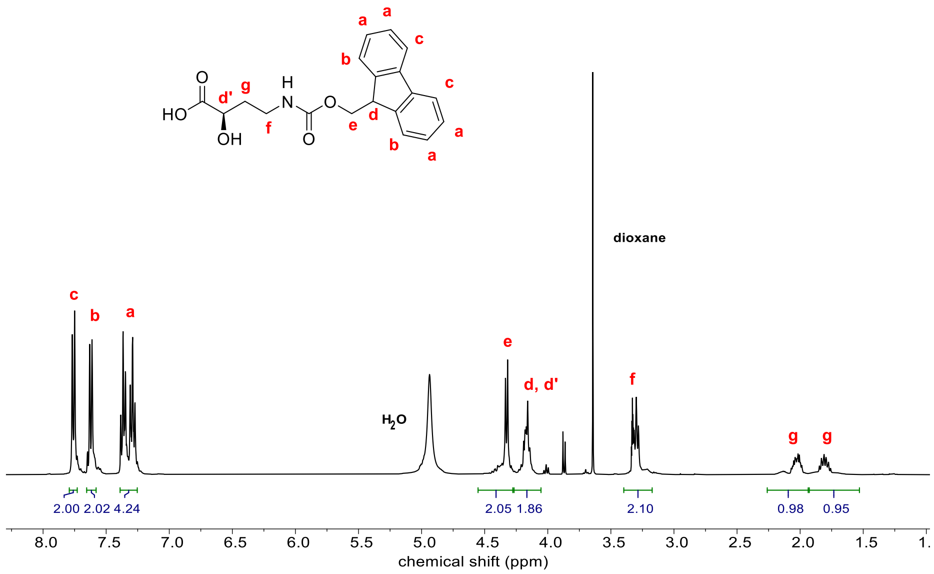

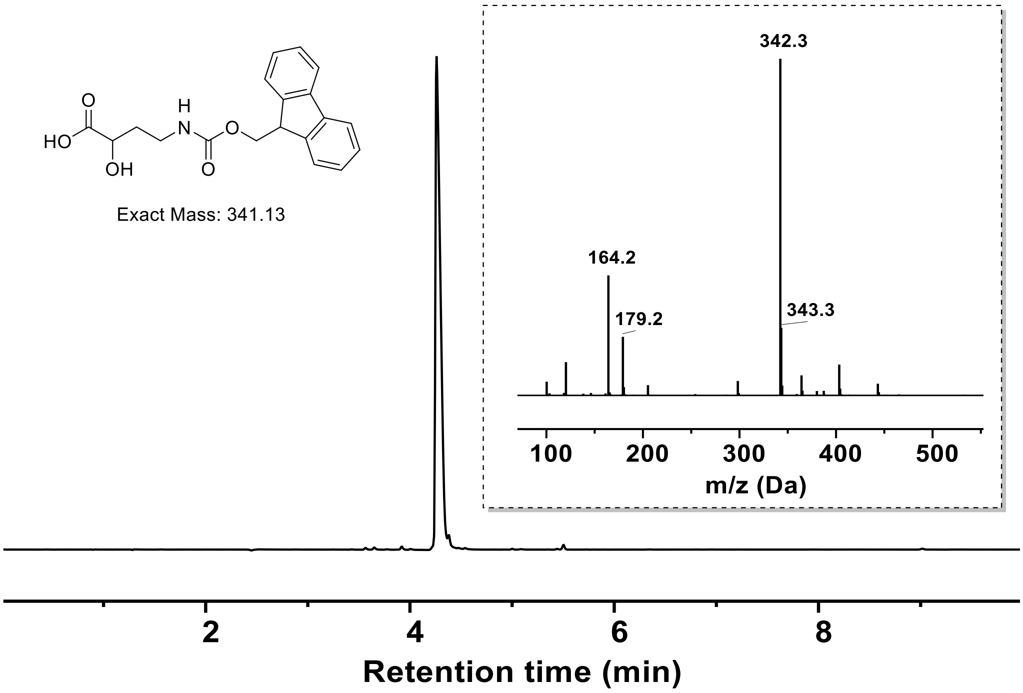

Figure S12: Structure of 4-(Fmoc)amino-2-hydroxybutyric acid, ^{1}H-NMR 400 MHz in MeOD and LC-MS chromatogram at 214 nm. MS spectra in the dashed line are obtained from integration of the peak shown in LC-MS. The signals on the side are from the fragmentation of the structure during the measurement (LC-MS in positive mode with ESI); main peak for the exact mass of the product is shown.

Synthesis of (1): 4-Fmoc(amino)-2-((propylcarbamoyl)oxy)butanoic acid

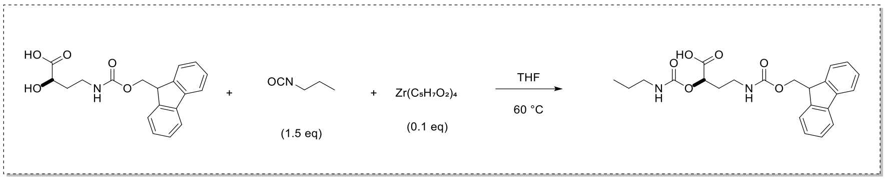

Scheme S2: Synthesis of propylcarbamoyl amino acid.

4-Fmoc(amino)-2-hydroxybutanoic acid (500 mg, 1.46 mmol, 1 eq.) and ($Zr(acac)_4$) (10.6 mg, 0.15 mmol, 0.1 eq.) were dissolved in 10 mL of anhydrous THF, then 1-propyl isocyanate (0.187 mL, 2.20 mmol, 1.5 eq) was added and the mixture was stirred for three hours at 60 °C under inert atmosphere (Argon). The reaction solution was evaporated, dissolved in dichloromethane (50 mL), and extracted with 1N HCl (20 mL x2) and brine (20 mL x2). The organic layers were collected and dried over $MgSO_4$ and evaporated. The product is obtained in the form of a powder, yield: 74 %.

HRMS: Peak mass: 427.18607; Theoretical mass: 427.18636

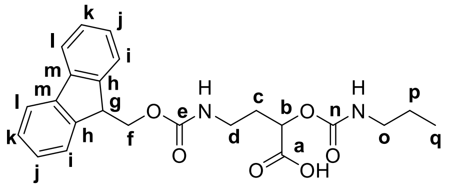

^{13}C NMR (MeOD, 125 MHz): δC 173.2 (a) 157.4 (e), 156.9 (n), 143.9 (h), 141.2 (m), 127.2 (i), 126.7 (j), 124.6 (k), 119.6 (l), 69.8 (b), 66.6 (f), 40.3 (g), 36.7 (c), 31.3 (o), 19.6 (q), 12.7 (r).

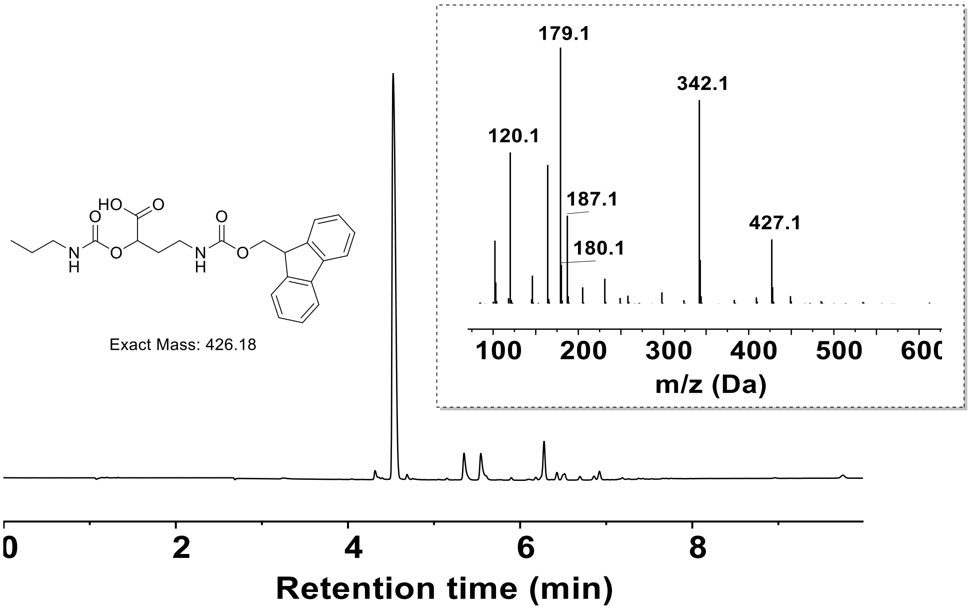

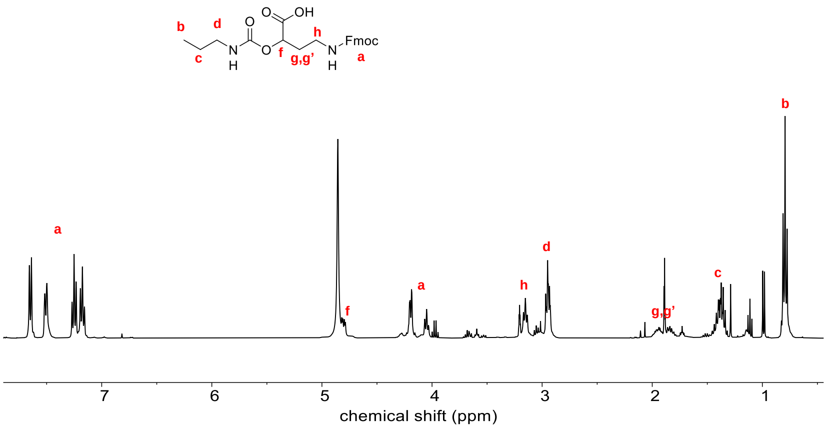

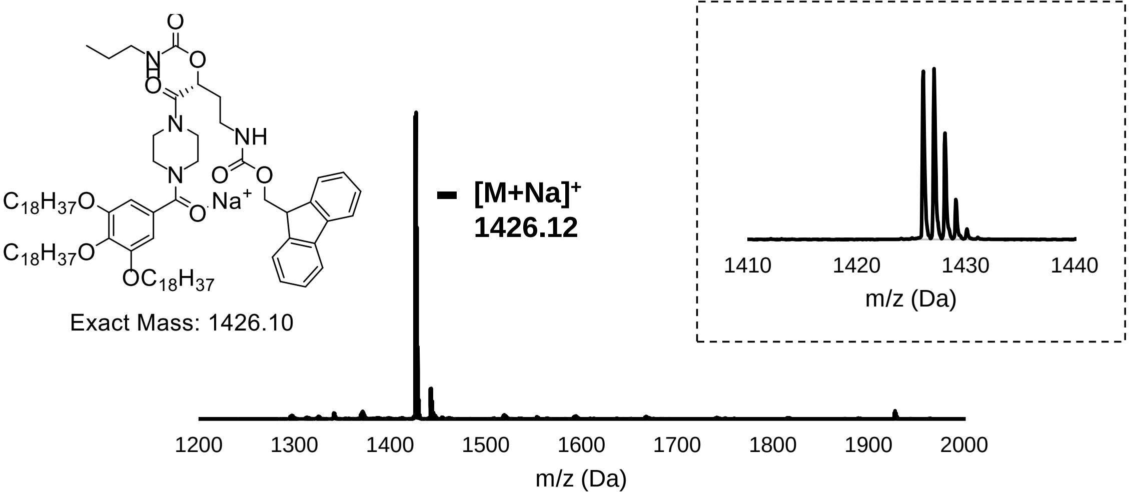

Figure S13: Structure of propylcarbamoyl-functionalized amino acid and LC-MS chromatogram at 214 nm; ¹H NMR 400 MHz, MeOD of propylcarbamoyl-functionalized amino acid; coupling test of monomer on non-cleavable support and MALDI-ToF of the structure. MS spectra in the dashed line are obtained from integration of the peak shown in LC-MS; main peaks are product and fragmentation.

Synthesis of (2): 4-Fmoc(amino)-2-((butylcarbamoyl)oxy)butanoic acid

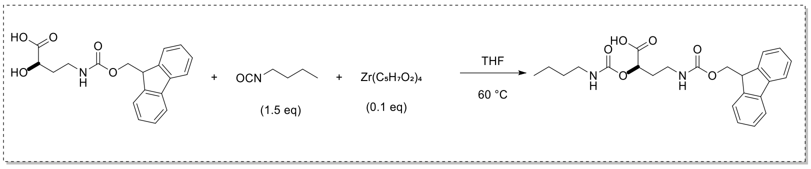

Scheme S3: Synthesis of butylcarbamoyl amino acid.

4-Fmoc(amino)-2-hydroxybutanoic acid (2 g, 5.76 mmol, 1 eq.) and ($Zr(acac)_4$) (42 mg, 0.58 mmol, 0.1 eq.) were dissolved in 20 mL of anhydrous THF, then 1-butyl isocyanate (1.13 mL, 8.79 mmol, 1.5 eq.) was added and the mixture was stirred for three hours at 60 °C under inert atmosphere (Argon). The reaction solution was evaporated, dissolved in dichloromethane (50 mL), and extracted with 1N HCl (20 mL x2) and brine (20 mL x2). The organic layers were collected and dried over $MgSO_4$ and evaporated. The product is obtained in the form of a powder, yield: 90 %.

HRMS: Peak mass: 439.18741; Theoretical mass: 439.18636

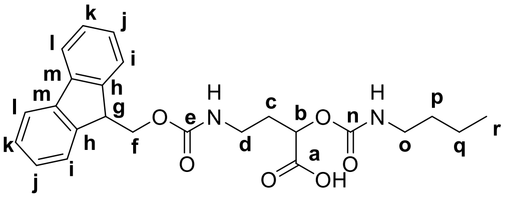

^{13}C NMR (MeOD, 125 MHz): δC 173.2 (a) 157.4 (e), 156.9 (n), 143.9 (h), 141.2 (m), 127.2 (i), 126.7 (j), 124.6 (k), 119.6 (l), 69.8 (b), 66.4 (f), 40.3 (g), 36.7 (o), 32.3 (p,c), 31.1 (d), 19.6 (q), 12.7 (r).

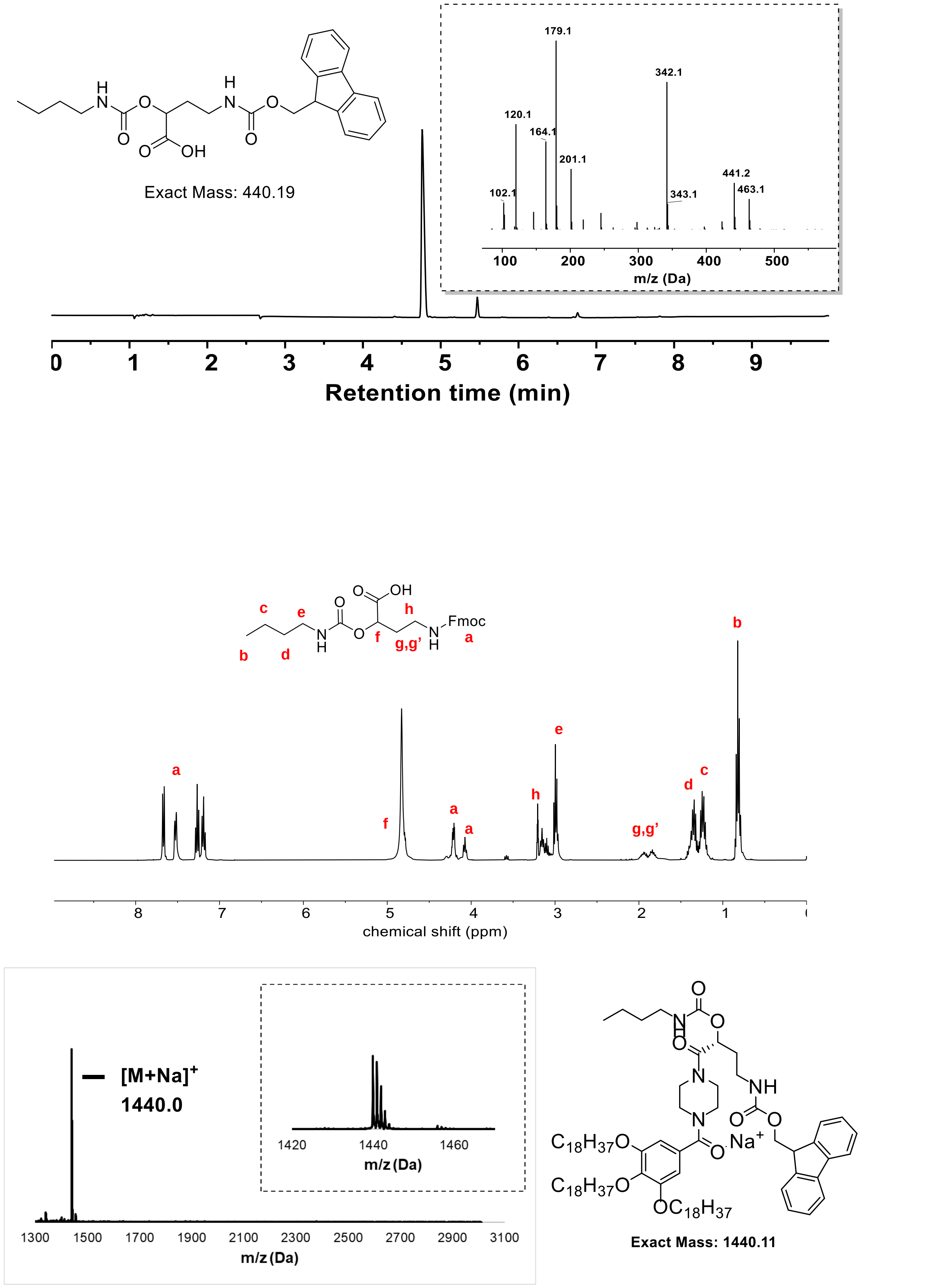

Figure S14: Structure of butylcarbamoyl amino acid and LC-MS chromatogram at 214 nm; ^{1}H NMR 400 MHz, MeOD of butylcarbamoyl amino acid; coupling test of monomer on non-cleavable support and MALDI-ToF of the structure. MS spectra in the dashed line are obtained from integration of the peak shown in LC-MS; main peaks are product and fragmentation.

Synthesis of (3): 4-Fmoc(amino)-2-((tertbutylcarbamoyl)oxy)butanoic acid

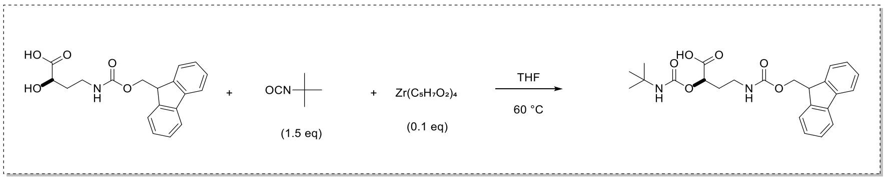

Scheme S4: Synthesis of tertbutylcarbamoyl amino acid.

4-Fmoc(amino)-2-hydroxybutanoic acid (500 mg g, 1.46 mmol, 1 eq.) and (Zr(acac)$_4$) (10.56 mg, 0.146 mmol, 0.1 eq.) were dissolved in 20 mL of anhydrous THF, then tertbutyl isocyanate (0.220 mL, 2.20 mmol, 1.5 eq) was added and the mixture was stirred for three hours at 60 °C under inert atmosphere (Argon). The reaction solution was evaporated, dissolved in dichloromethane (50 mL), and extracted with 1N HCl (20 mL x2) and brine (20 mL x2). The organic layers were collected and dried over $MgSO_4$ and evaporated. The product is obtained in the form of a powder, yield: 60 %.

HRMS: Peak Mass: 441.20160; Theoretical Mass: 441.20201

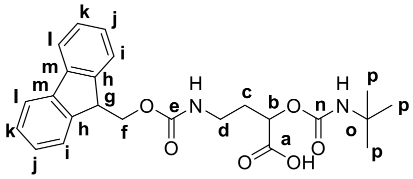

^{13}C NMR (MeOD, 125 MHz): δC 173.2 (a) 157.4 (e), 156.9 (n), 143.9 (h), 141.2 (m), 127.2 (i), 126.7 (j), 124.6 (k), 119.6 (l), 69.8 (b), 66.4 (f), 56.6 (o), 40.3 (g), 36.7 (o).

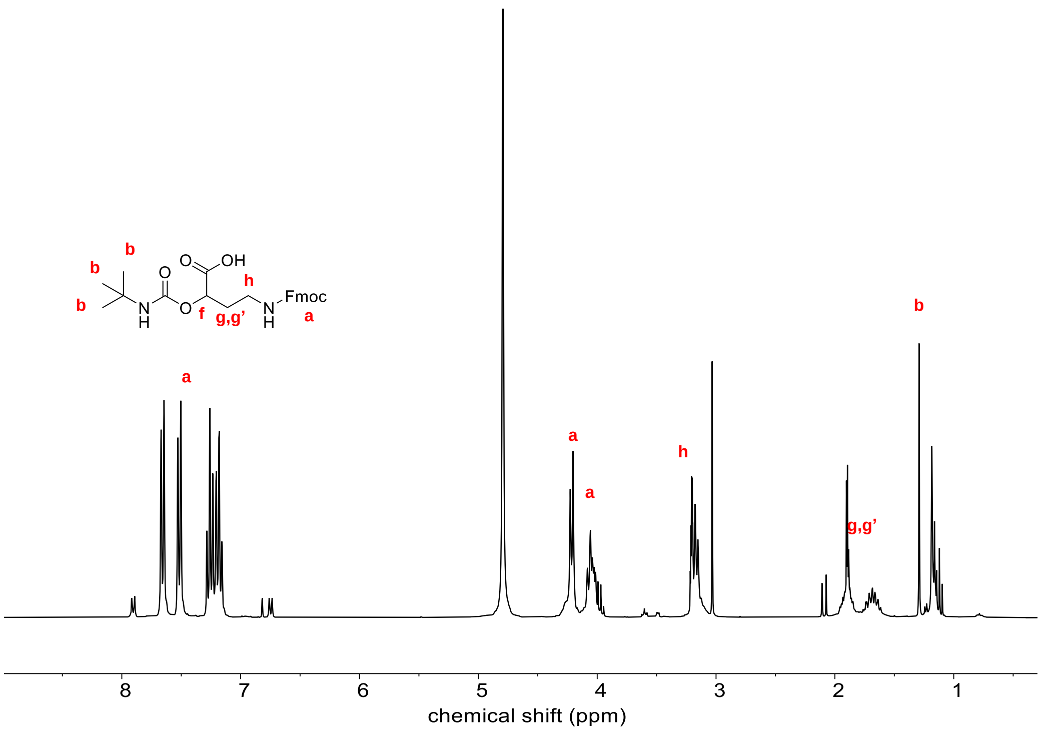

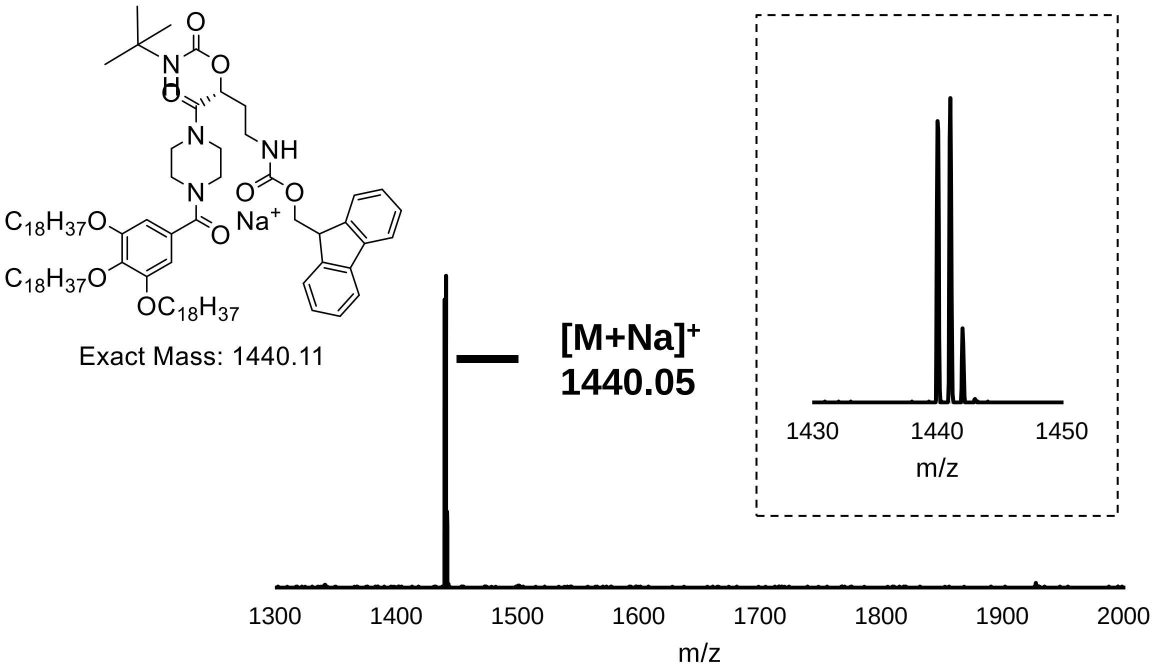

Figure S15: ^{1}H NMR 400 MHz, MeOD of tertbutylcarbamoyl-functionalized amino acid; coupling test of monomer on non-cleavable support and MALDI-ToF of the structure.

Synthesis of (4): 4-Fmoc(amino)-2-((hexylcarbamoyl)oxy)butanoic acid

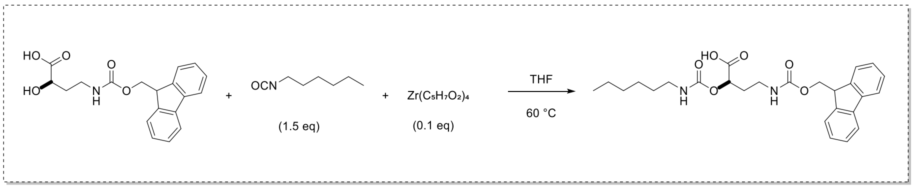

Scheme S5: Synthesis of hexylcarbamoyl amino acid.

4-Fmoc(amino)-2-hydroxybutanoic acid (2 g, 5.76 mmol, 1 eq.) and ($Zr(acac)_4$) (42 mg, 0.58 mmol, 0.1 eq.) were dissolved in 20 mL of anhydrous THF, then 1-hexyl isocyanate (1.28 mL, 8.79 mmol, 1.5 eq.) was added and the mixture was stirred for three hours at 60 °C under inert atmosphere (Argon). The reaction solution was evaporated, dissolved in dichloromethane (50 mL), and extracted with 1N HCl (20 mL x2) and brine (20 mL x2). The organic layers were collected and dried over $MgSO_4$ and evaporated. The product is obtained in the form of a powder, yield: 93 %.

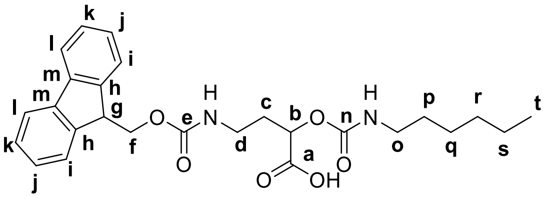

^{13}C NMR (MeOD, 125 MHz): δC 173.2 (a) 157.4 (e), 156.9 (n), 143.9 (h), 141.2 (m), 127.2 (i), 126.7 (j), 124.6 (k), 119.6 (l), 69.8 (b), 66.4 (f), 40.3 (g), (o), 32.3 (c), 31.1 (d), 30.3 (o), 28.7 (p) 22.5 (q,r) 19.68 (s), 13.1(t).

HRMS: Peak Mass: 467.21857; Theoretical Mass: 467.21766

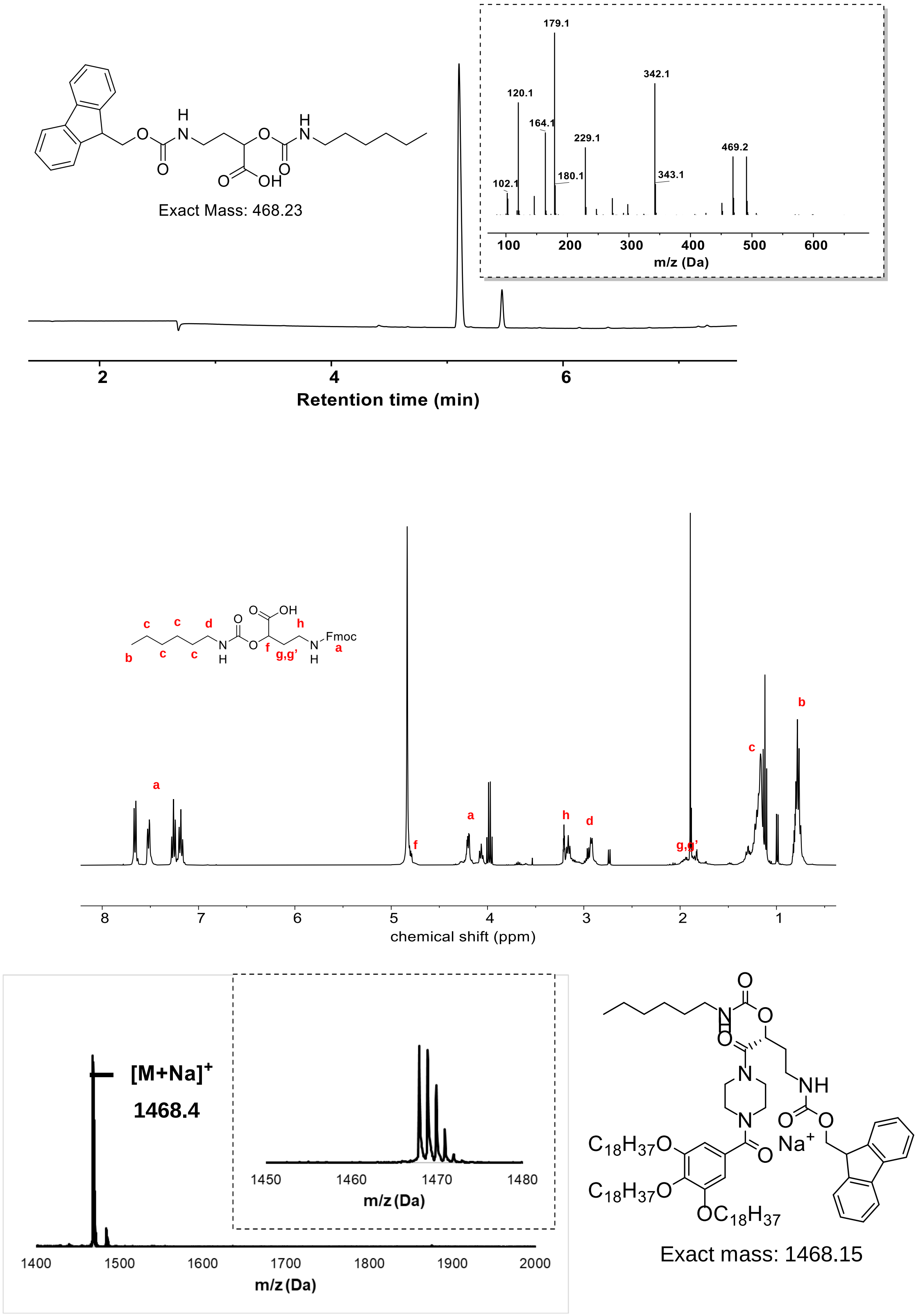

Figure S16: Structure of hexylcarbamoyl-functionalized amino acid and LC-MS chromatogram at 214 nm; ^{1}H NMR 400 MHz, MeOD of hexylcarbamoyl-functionalized amino acid; coupling test of monomer on non-cleavable support and MALDI-ToF. MS spectra in the dashed line are obtained from integration of the peak shown in LC-MS; main peaks are product and fragmentation.

Synthesis of (5): 4-Fmoc(amino)-2-((ethylhexylcarbamoyl)oxy)butanoic acid

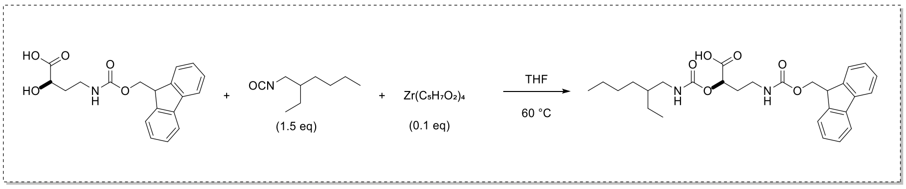

Scheme S6: Synthesis of 2-ethylhexylcarbamoyl-functionalized amino acid.

4-Fmoc(amino)-2-hydroxybutanoic acid (2 g, 5.76 mmol, 1 eq.) and (Zr(acac)$_4$) (42 mg, 0.58 mmol, 0.1 eq.) were dissolved in 20 mL of anhydrous THF, then 2-ethylhexyl isocyanate (1.52 mL, 8.79 mmol, 1.5 eq.) was added and the mixture was stirred for three hours at 60 °C under inert atmosphere (Argon). The reaction solution was evaporated, dissolved in dichloromethane (50 mL), and extracted with 1N HCl (20 mL x2) and brine (20 mL x2). The organic layers were collected and dried over $MgSO_4$ and evaporated. The product is obtained in the form of a powder, yield: 92 %.

HRMS: Peak Mass: 495.24991; Theoretical mass: 495.25006

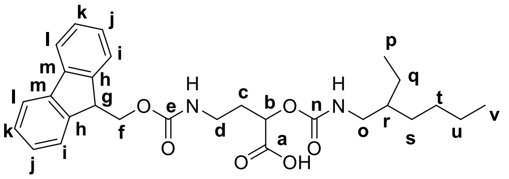

^{13}C NMR (MeOD, 125 MHz): δC 173.2 (a) 157.4 (e), 156.9 (n), 143.9 (h), 141.2 (m), 127.2 (i), 126.7 (j), 124.6 (k), 119.6 (l), 69.8 (b), 66.4 (f), 43.3 (g), 43.8 (o), 39.7 (r), 32.3 (c), 31.2 (t), 29.6 (s), 25.3 (q), 23.7 (u), 13.9 (v), 11.4 (p).

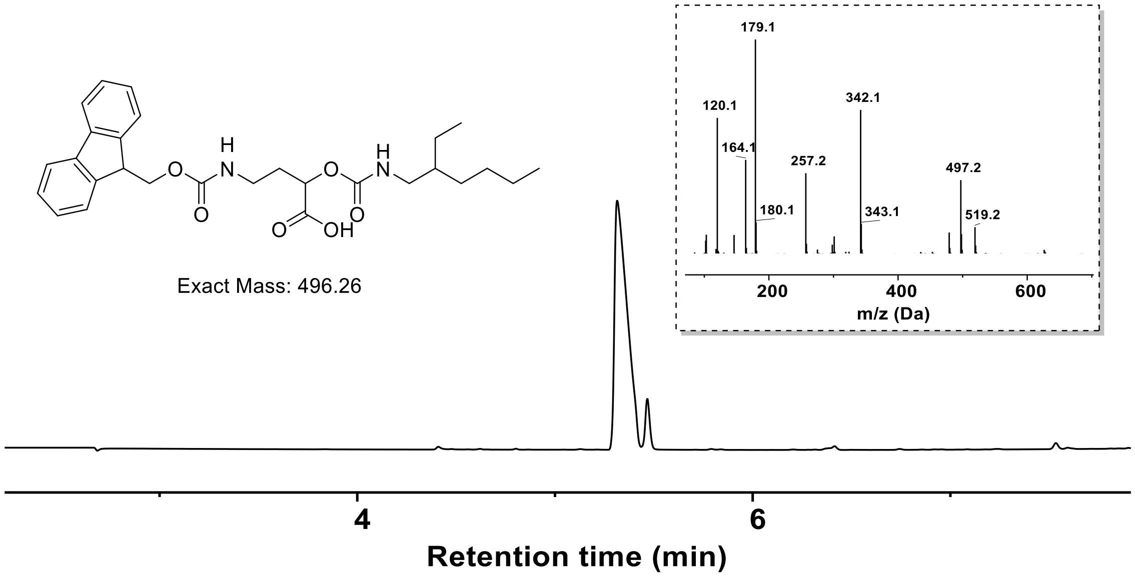

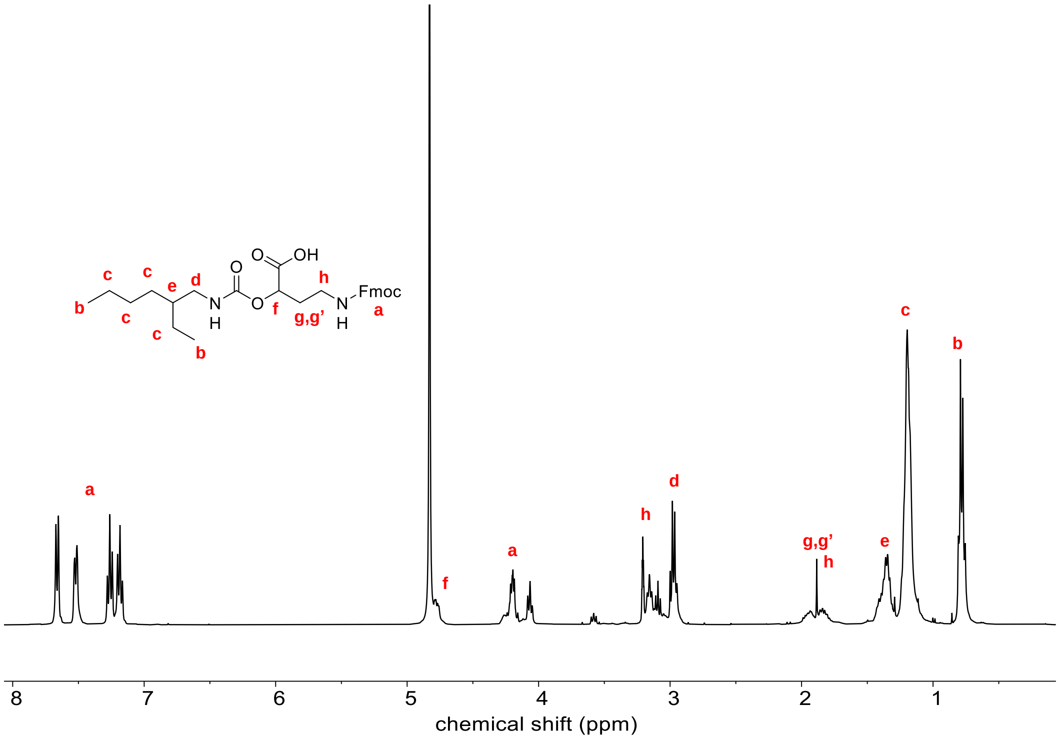

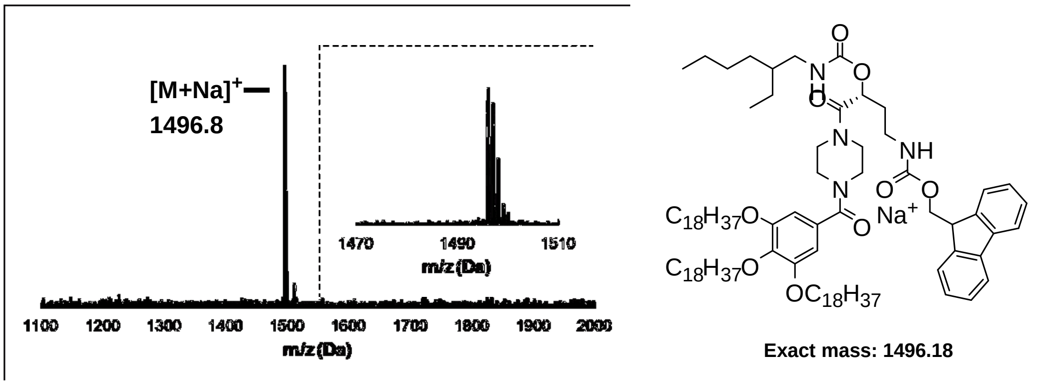

Figure S17: Structure of 2-ethylhexylcarbamoyl amino acid and LC-MS chromatogram at 214 nm; ^{1}H NMR 400 MHz, MeOD of 2-ethylhexylcarbamoyl amino acid; coupling test of monomer on non-cleavable support and MALDI-ToF of the structure. MS spectra in the dashed line are obtained from integration of the peak shown in LC-MS; main peaks are product and fragmentation.

Synthesis of (6): 4-Fmoc(amino)-2-((benzylcarbamoyl)oxy)butanoic acid

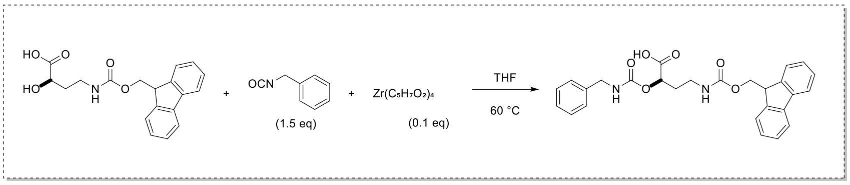

Scheme S7: Synthesis of benzyl-functionalized amino acid.

4-Fmoc(amino)-2-hydroxybutanoic acid (2 g, 5.78 mmol, 1 eq.) and (Zr(acac)$_4$) (42 mg, 0.58 mmol, 0.1 eq.) were dissolved in 20 mL of anhydrous THF, then benzyl isocyanate (1.09 mL, 8.79 mmol, 1.5 eq.) was added and the mixture was stirred for three hours at 60 °C under inert atmosphere (Argon). The reaction solution was evaporated, dissolved in dichloromethane (50 mL), and extracted with 1N HCl (20 mL x2) and brine (20 mL x2). The organic layers were collected and dried over $MgSO_4$ and evaporated. The product is obtained in the form of a powder, yield: 83 %.

HRMS: Peak Mass: 473.17191; Theoretical Mass: 473.17071

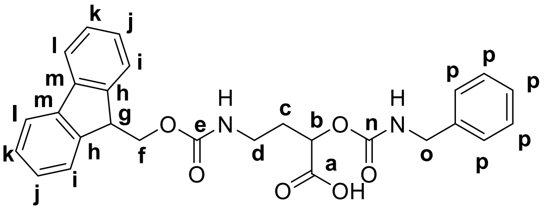

^{13}C NMR (MeOD, 125 MHz): δC 173.2 (a) 157.4 (e), 156.9 (n), 143.9 (h), 141.2 (m), 127.2 (i), 126.7 (j), 128.2-126.4 (p), 124.6 (k), 119.6 (l), 69.8 (b), 66.4 (f), 41.9 (o) 40.3 (g), 36.7 (o), 32.3 (c), 31.1 (d).

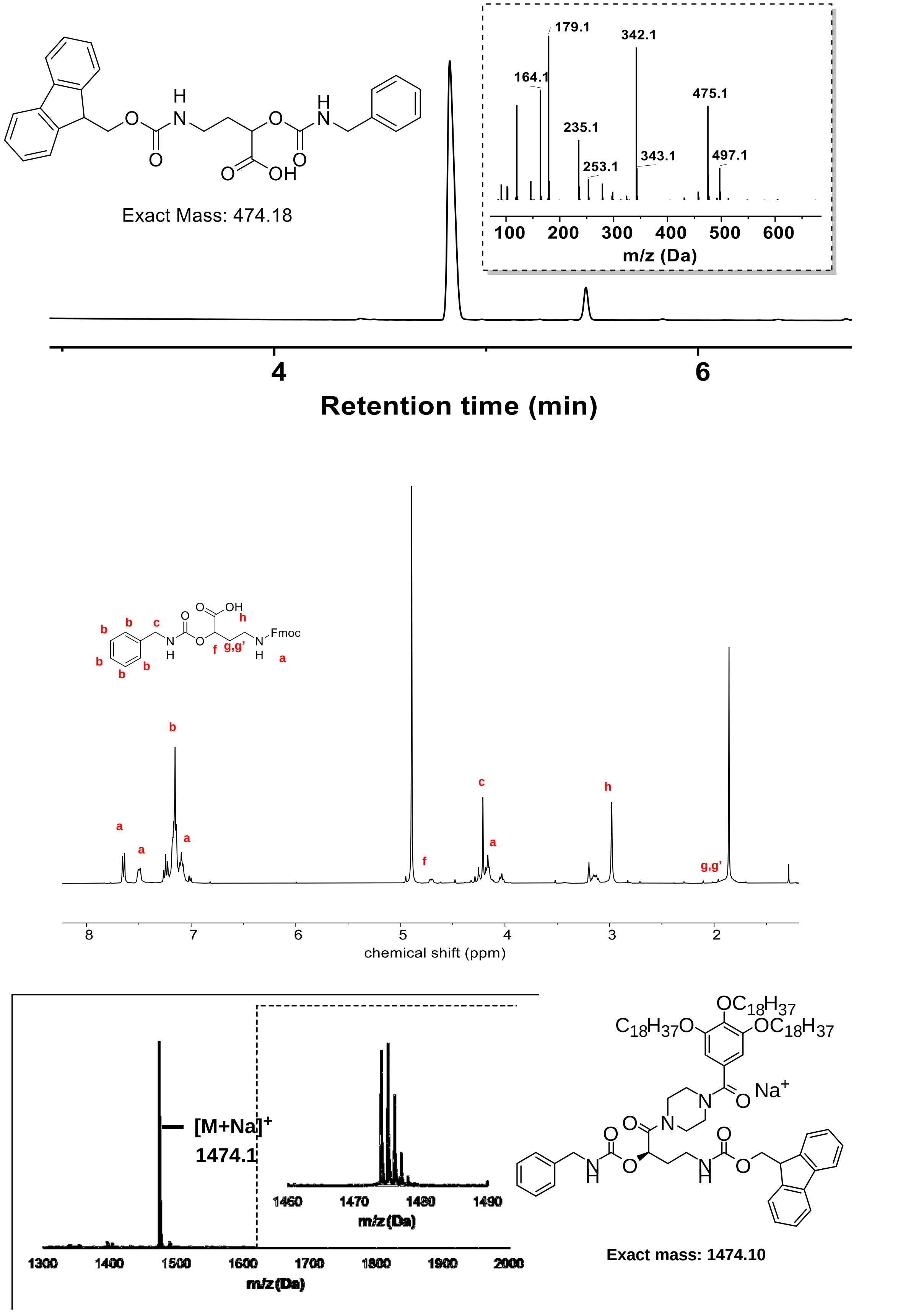

Figure S18: Structure of benzylcarbamoyl-functionalized amino acid and LC-MS chromatogram at 214 nm; ^{1}H NMR 400 MHz, MeOD of benzylcarbamoyl-functionalized amino acid; coupling test of monomer on non-cleavable support and MALDI-ToF of the structure. MS spectra in the dashed line are obtained from integration of the peak shown in LC-MS; main peaks are product and fragmentation.

Synthesis of (7): 4-Fmoc(amino)-2-((phenylcarbamoyl)oxy)butanoic acid

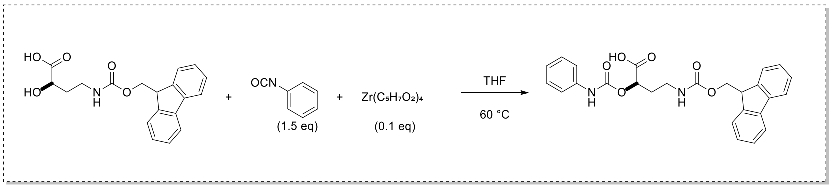

Scheme S8: Synthesis of phenylcarbamoyl amino acid.

4-Fmoc(amino)-2-hydroxybutanoic acid (500 mg, 1.46 mmol, 1 eq.) and (Zr(acac)$_4$) (10.56 mg, 0.146 mmol, 0.1 eq.) were dissolved in 10 mL of anhydrous THF, then phenyl isocyanate (0.262 mL, 2.20 mmol, 1.5 eq) was added and the mixture was stirred for three hours at 60 °C under inert atmosphere (Argon). The reaction solution was evaporated, dissolved in dichloromethane (50 mL), and extracted with 1N HCl (20 mL x2) and brine (20 mL x2). The organic layers were collected and dried over $MgSO_4$ and evaporated. The product is obtained in the form of a powder, yield: 94 %.

HRMS: Peak Mass: 459.15634; Theoretical Mass: 459.15640.

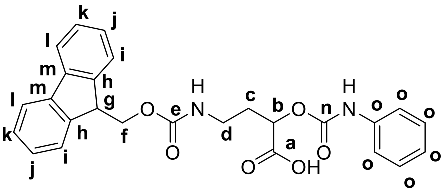

^{13}C NMR (MeOD, 125 MHz): δC 173.2 (a) 157.4 (e), 156.9 (n), 143.9 (h), 141.2 (m), 127.2 (i), 126.7 (j), 124.6 (k), 128.6-121.7 (o), 119.6 (l), 69.8 (b), 66.4 (f), 40.3 (g), 32.3 (c), 31.1 (d).

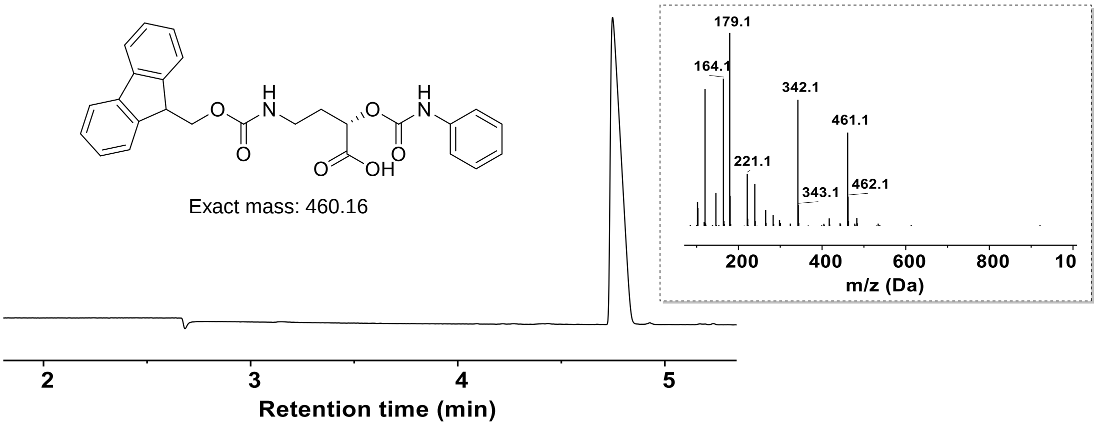

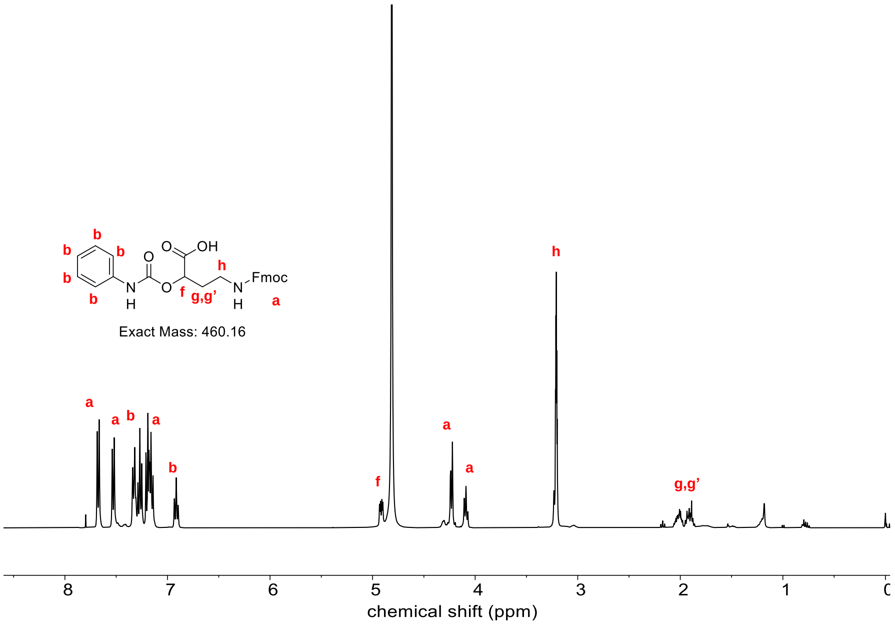

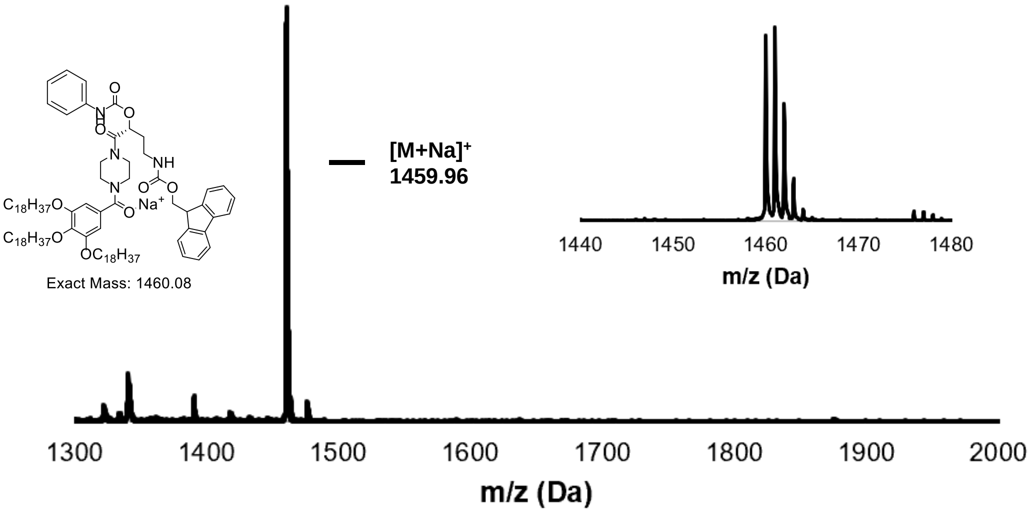

Figure S19: Structure of phenylcarbamoyl-modified amino acid and LC-MS chromatogram at 214 nm; ¹H NMR 400 MHz, MeOD of phenylcarbamoyl-modified amino acid; coupling test of monomer on non-cleavable support and MALDI-ToF of the structure. MS spectra in the dashed line are obtained from integration of the peak shown in LC-MS; main peaks are product and fragmentation.

Synthesis of (8): 4-Fmoc(amino)-2-(*p*-tolylcarbamoyloxy)butanoic acid

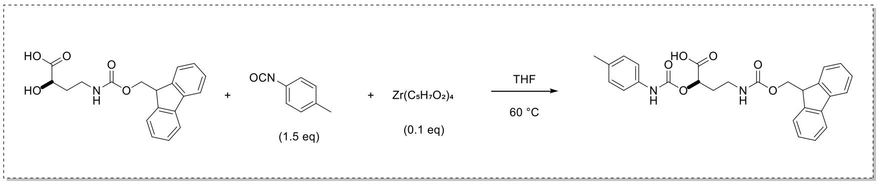

Scheme S9: Synthesis of p-tolylcarbamoyl-functionalized amino acid.

4-Fmoc(amino)-2-hydroxybutanoic acid (500 mg, 1.46 mmol, 1 eq.) and (Zr(acac)$_4$) (10.56 mg, 0.146 mmol, 0.1 eq.) were dissolved in 10 mL of anhydrous THF, then *p*-tolyl isocyanate (0.262 mL, 2.20 mmol, 1.5 eq) was added and the mixture was stirred for three hours at 60 °C under inert atmosphere (Argon). The reaction solution was evaporated, dissolved in dichloromethane (50 mL), and extracted with 1N HCl (20 mL x2) and brine (20 mL x2). The organic layers were collected and dried over $MgSO_4$ and evaporated. The product is obtained in the form of a powder, yield: 92 %.

HRMS: Peak Mass: 475.18587; Theoretical Mass: 475.18636

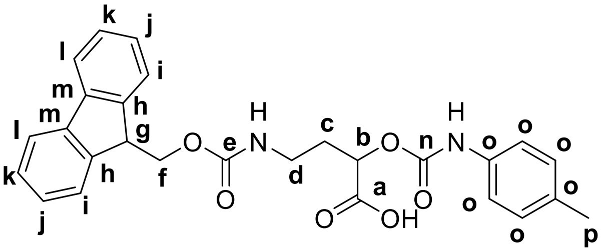

^{13}C NMR (MeOD, 125 MHz): δC 173.2 (a) 157.4 (e), 156.9 (n), 143.9 (h), 141.2 (m), 127.2 (i), 126.7 (j), 124.6 (k), 128.4-122.2 (o), 119.6 (l), 69.8 (b), 66.4 (f), 40.3 (g), 36.7 (o), 32.3 (c), 31.1 (d), 21.3 (p).

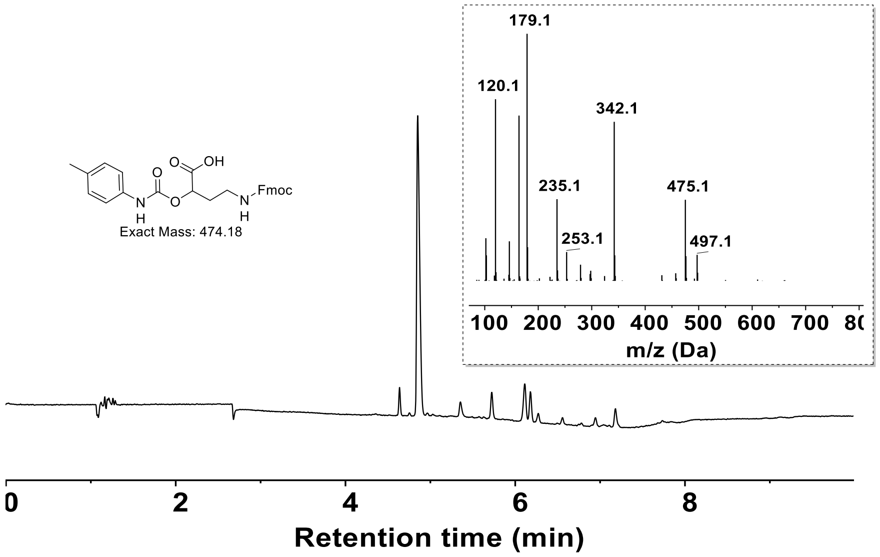

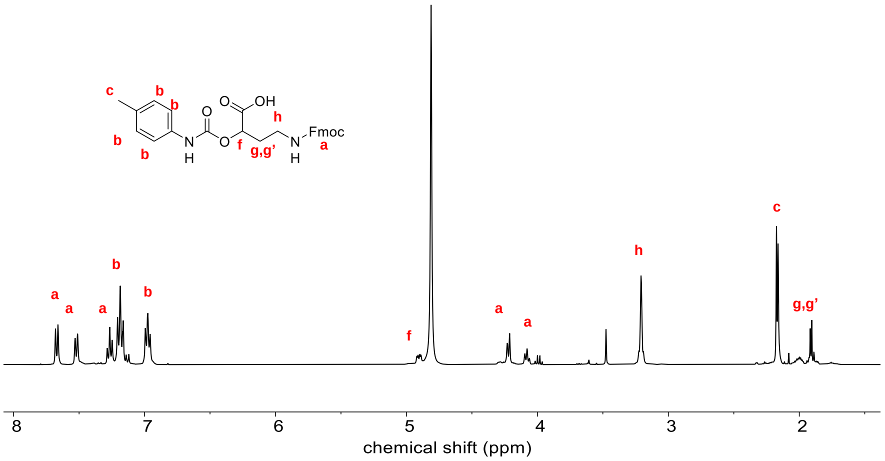

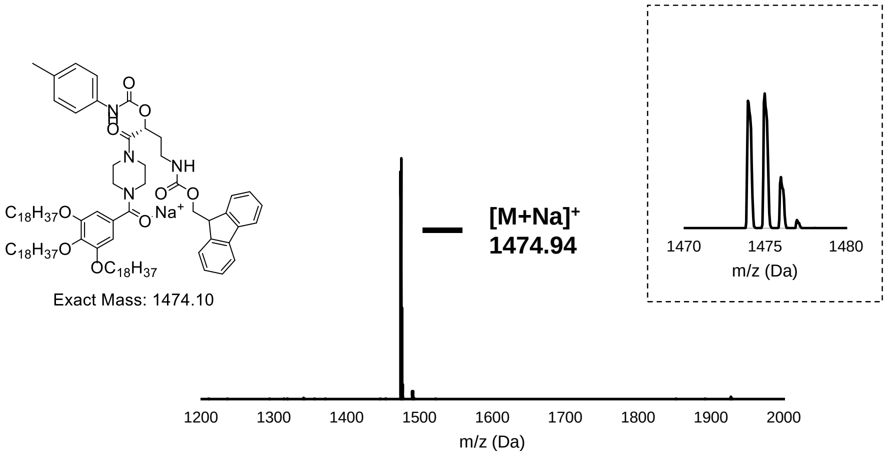

Figure S20: Structure of p-tolylcarbamoyl-modified amino acid and LC-MS chromatogram at 214 nm; ^{1}H NMR 400 MHz, MeOD of p-tolylcarbamoyl-modified amino acid; coupling test of monomer on non-cleavable support and MALDI-ToF of the structure. MS spectra in the dashed line are obtained from integration of the peak shown in LC-MS; main peaks are product and fragmentation.

Synthesis of (9): 4-Fmoc(amino)-2-(4-ethylphenylcarbamoyloxy)butanoic acid

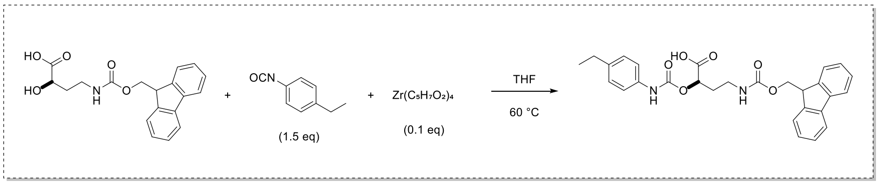

Scheme S10: Synthesis of 4-ethyl phenylcarbamoyl-modified amino acid.

4-Fmoc(amino)-2-hydroxybutanoic acid (500 mg, 1.46 mmol, 1 eq.) and ($Zr(acac)_4$) (10.56 mg, 0.146 mmol, 0.1 eq.) were dissolved in 10 mL of anhydrous THF, then 4-ethyl phenyl isocyanate (0.323 mL, 2.20 mmol, 1.5 eq) was added and the mixture was stirred for three hours at 60 °C under inert atmosphere (Argon). The reaction solution was evaporated, dissolved in dichloromethane (50 mL), and extracted with 1N HCl (20 mL x2) and brine (20 mL x2). The organic layers were collected and dried over $MgSO_4$ and evaporated. The product is obtained in the form of a powder, yield: 90 %.

HRMS: Peak Mass: 489.20180; Theoretical Mass: 489.20201

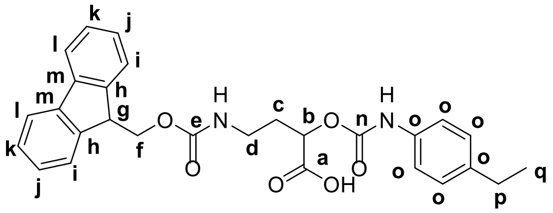

^{13}C NMR (MeOD, 125 MHz): δC 173.2 (a) 157.4 (e), 156.9 (n), 143.9 (h), 141.2 (m), 127.2 (i), 126.7 (j), 124.6 (k), 128.4-122.2 (o), 119.6 (l), 69.8 (b), 66.4 (f), 40.3 (g), 32.3 (c), 31.1 (d), 22.1 (p), 15.1 (q).

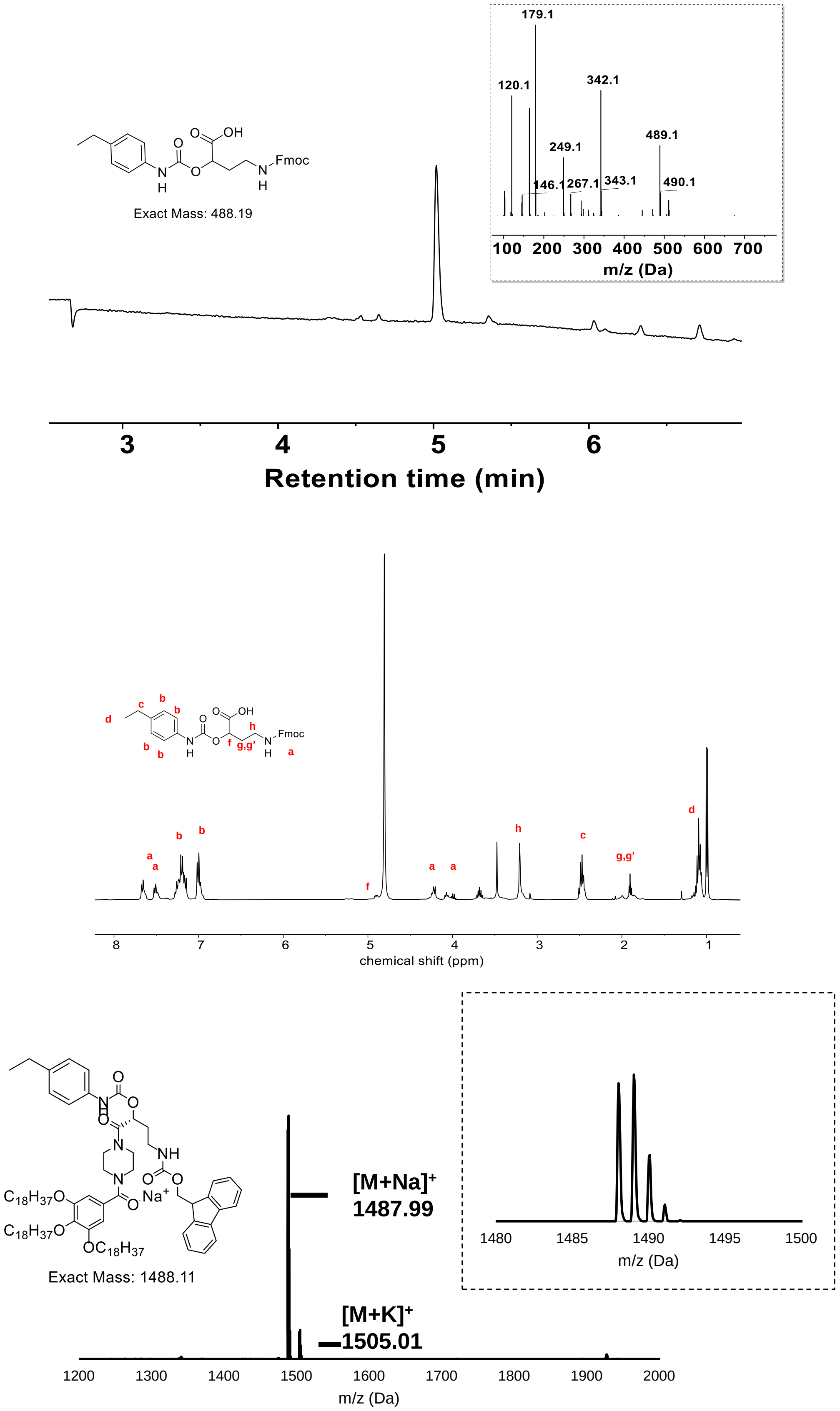

Figure S21: Structure of 4-ethylphenylcarbamoyl amino acid and LC-MS chromatogram at 214 nm; ^{1}H NMR 400 MHz, MeOD of 4-ethylphenylcarbamoyl amino acid; coupling test of monomer on non-cleavable support and MALDI-ToF of the structure. MS spectra in the dashed line are obtained from integration of the peak shown in LC-MS; main peaks are product and fragmentation.

Synthesis of (10): 4-Fmoc(amino)-2-(4-fluorophenylcarbamoyloxy)butanoic acid

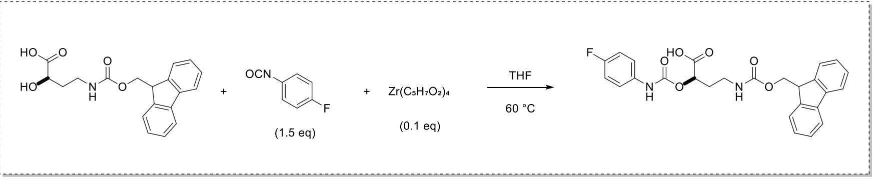

Scheme S11: Synthesis of 4-fluoro phenylcarbamoyl-modified amino acid.

4-Fmoc(amino)-2-hydroxybutanoic acid (500 mg, 1.46 mmol, 1 eq.) and (Zr(acac)$_4$) (10.56 mg, 0.146 mmol, 0.1 eq.) were dissolved in 10 mL of anhydrous THF, then 4-fluoro phenyl isocyanate (0.300 mL, 2.20 mmol, 1.5 eq) was added and the mixture was stirred for three hours at 60 °C under inert atmosphere (Argon). The reaction solution was evaporated, dissolved in dichloromethane (50 mL), and extracted with 1N HCl (20 mL x2) and brine (20 mL x2). The organic layers were collected and dried over $MgSO_4$ and evaporated. The product is obtained in the form of a powder, yield: 92 %.

HRMS: Peak Mass: 479.16068; Theoretical Mass: 479.16129

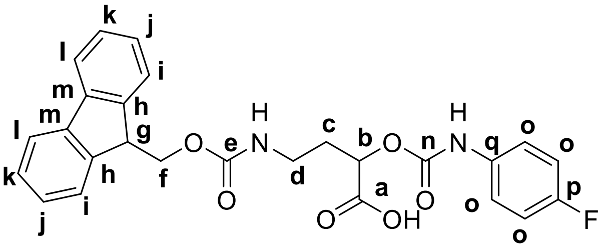

^{13}C NMR (MeOD, 125 MHz): δC 173.2 (a), 159.6 (q), 157.4 (e), 156.9 (n), 143.9 (h), 141.2 (m), 133.6 (q), 127.2 (i), 126.7 (j), 124.6 (k), 119.6 (l), 115.2 (p) 69.8 (b), 66.4 (f), 40.3 (g), 32.3 (c), 31.1 (d).

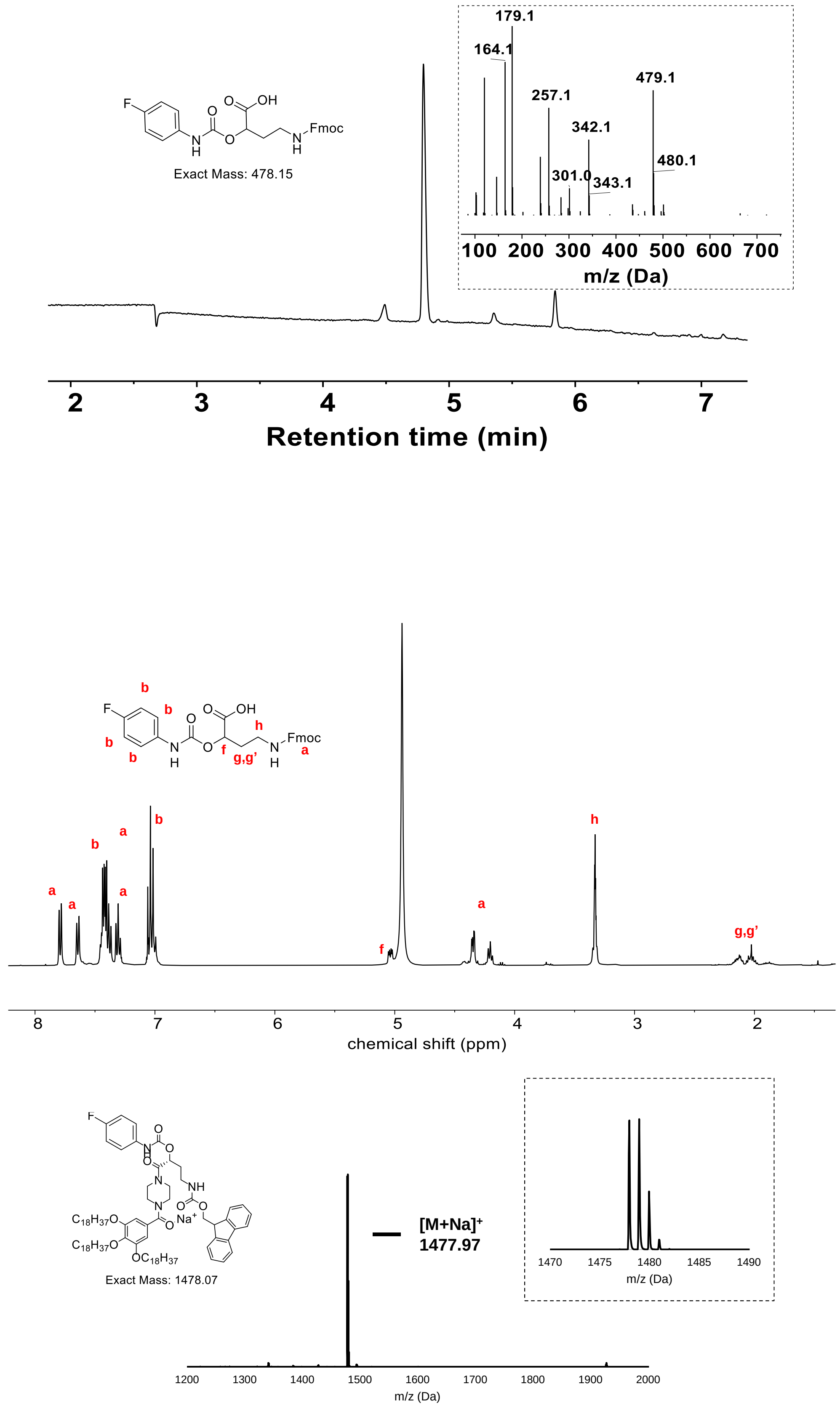

Figure S22: Structure of 4-fluorophenyllcarbamoyl amino acid and LC-MS chromatogram at 214 nm; ^{1}H NMR 400 MHz, MeOD of 4-fluorophenylcarbamoyl amino acid; coupling test of monomer on non-cleavable support and MALDI-ToF of the structure. MS spectra in the dashed line are obtained from integration of the peak shown in LC-MS; main peaks are product and fragmentation.

Synthesis of (11): 4-Fmoc(amino)-2-(4-nitrobenzoyloxy)butanoic acid

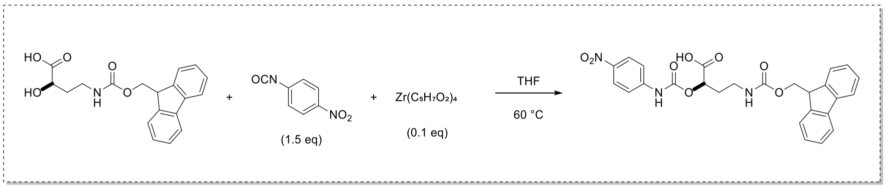

Scheme S12: Synthesis of 4-nitrophenylcarbamoyl-functionalized amino acid.

4-Fmoc(amino)-2-hydroxybutanoic acid (2 g, 5.76 mmol, 1 eq.) and ($Zr(acac)_4$) (42 mg, 0.58 mmol, 0.1 eq.) were dissolved in 20 mL of anhydrous THF, then 4-nitro phenyl isocyanate (1.44 g, 8.79 mmol, 1.5 eq.) was added and the mixture was stirred for three hours at 60 °C under inert atmosphere (Argon). The reaction solution was evaporated, dissolved in dichloromethane (50 mL), and extracted with 1N HCl (20 mL x2) and brine (20 mL x2). The organic layers were collected and dried over $MgSO_4$ and evaporated. The product is obtained in the form of a powder, yield: 95 %.

Exact Mass: 504.141151, Theoretical Mass: 504.14124

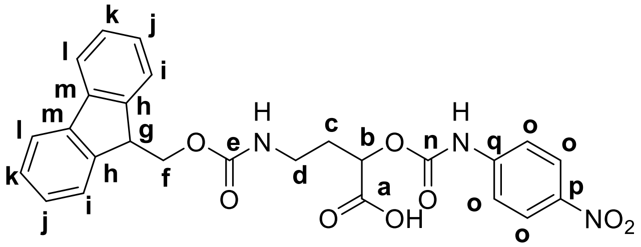

^{13}C NMR (MeOD, 125 MHz): δC 173.2 (a), 157.4 (e), 156.9 (n), 143.9 (h, q, p), 141.2 (m), 127.2 (i), 126.7 (j), 124.6 (k,o), 119.6 (l,o), 69.8 (b), 66.4 (f), 40.3 (g), 32.3 (c), 31.1 (d).

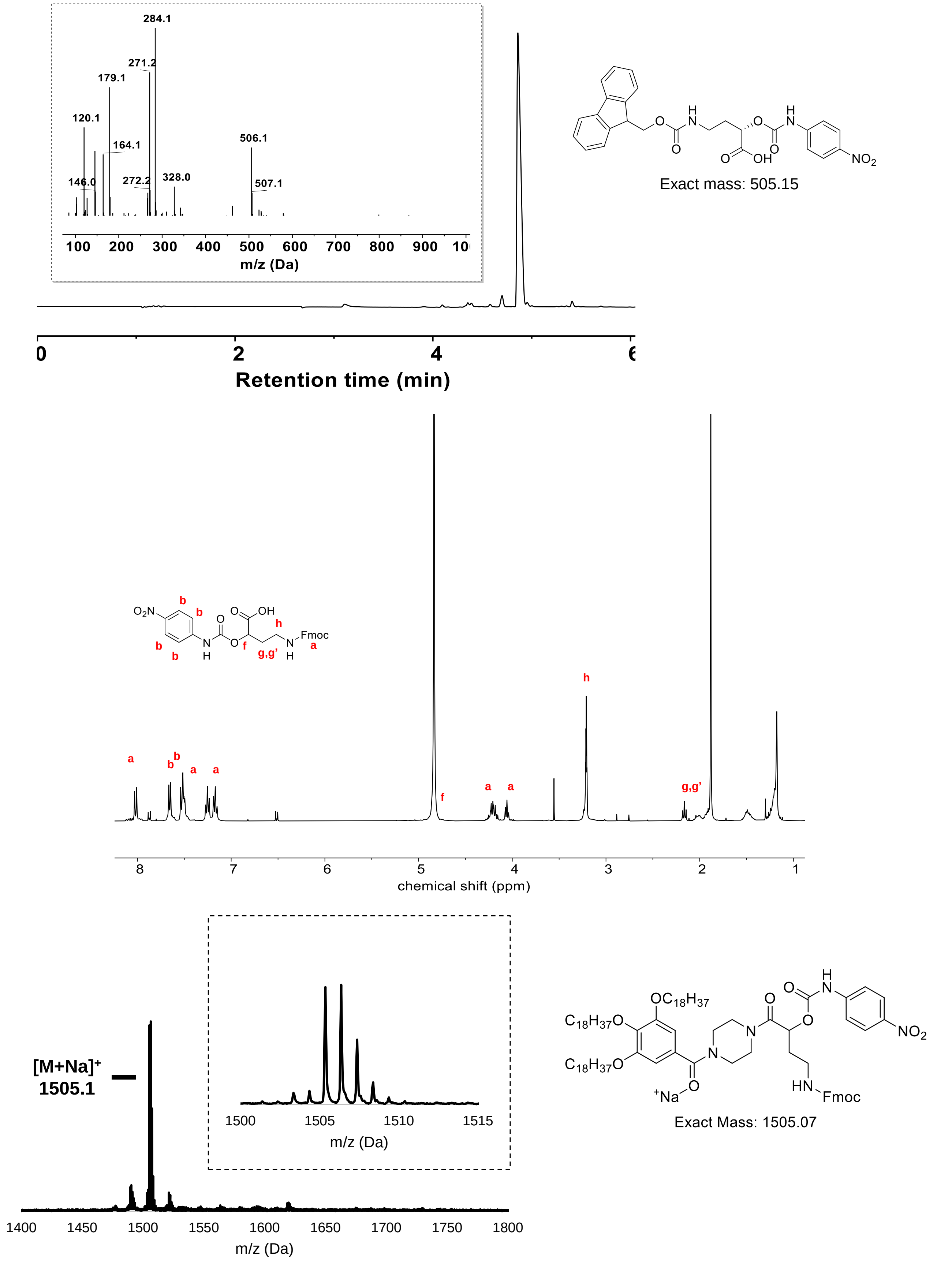

Figure S23: Structure of 4-nitrophenylcarbamoyl amino acid and LC-MS chromatogram at 214 nm; ^{1}H NMR 400 MHz, MeOD of 4-nitrophenylcarbamoyl amino acid; coupling test of monomer on non-cleavable support and MALDI-ToF of the structure. MS spectra in the dashed line are obtained from integration of the peak shown in LC-MS; main peaks are product and fragmentation.

Synthesis of (12): 4-Fmoc(amino)-2-((napthoyl)oxy)butanoic acid

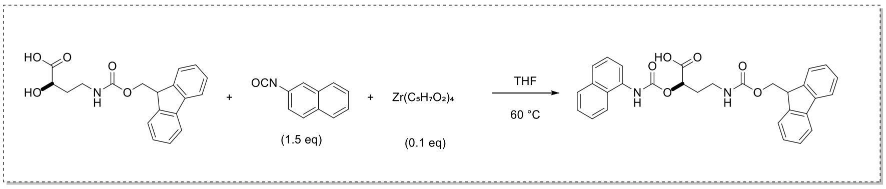

Scheme S13: Synthesis of naphtoylcarbamoyl-functionalized amino acid.

4-Fmoc(amino)-2-hydroxybutanoic acid (2 g, 5.76 mmol, 1 eq.) and ($Zr(acac)_4$) (42 mg, 0.58 mmol, 0.1 eq.) were dissolved in 20 mL of anhydrous THF, then 1-naphtoyl isocyanate (1.26 mL, 8.79 mmol, 1.5 eq.) was added and the mixture was stirred for three hours at 60 °C under inert atmosphere (Argon). The reaction solution was evaporated, dissolved in dichloromethane (50 mL), and extracted with 1N HCl (20 mL x2) and brine (20 mL x2). The organic layers were collected and dried over $MgSO_4$ and evaporated. The product is obtained in the form of a powder, yield: 80 %.

HRMS: Peak Mass: 511.18604; Theoretical Mass: 511.18636

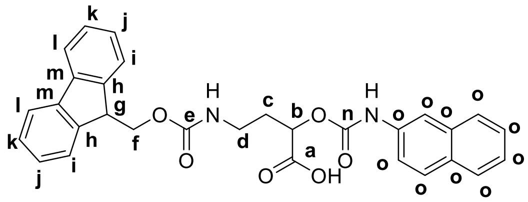

^{13}C NMR (MeOD, 125 MHz): δC 173.2 (a), 157.4 (e), 156.9 (n), 143.9 (h), 141.2 (m), 127.2 (i), 126.7 (j), 124.6 (k), 119.6 (l), 134.2-119.7 (o) 69.8 (b), 66.4 (f), 40.3 (g), 32.3 (c), 31.1 (d).

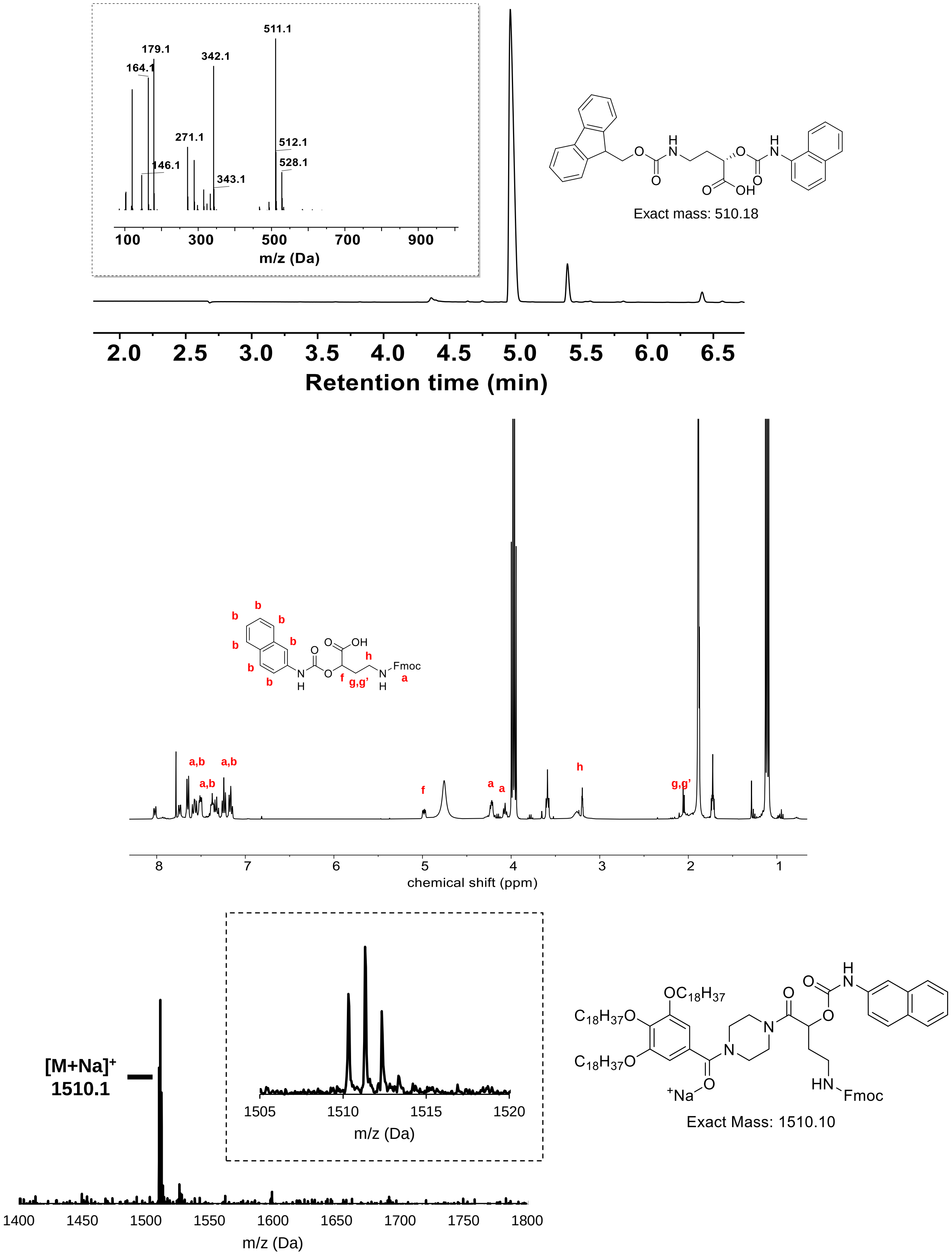

Figure S24: Structure of naphtoylcarbamoyl-functionalized amino acid and LC-MS chromatogram at 214 nm; ^{1}H NMR 400 MHz, MeOD of naphtoylcarbamoyl-functionalized amino acid; coupling test of monomer on non-cleavable support and MALDI-ToF of the structure. MS spectra in the dashed line are obtained from integration of the peak shown in LC-MS; main peaks are product and fragmentation.

Synthesis of (13): 4-Fmoc(amino)-2-((allylcarbamoyl)oxy)butanoic acid

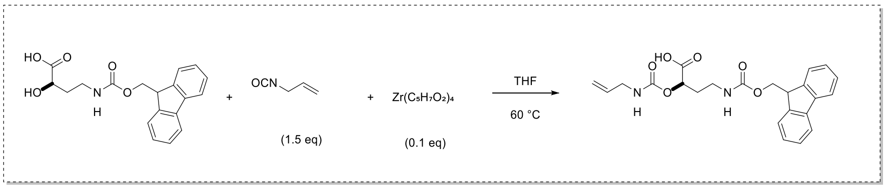

Scheme S14: Synthesis of allylcarbamoyl-functionalized amino acid.

4-Fmoc(amino)-2-hydroxybutanoic acid (2 g, 5.76 mmol, 1 eq.) and ($Zr(acac)_4$) (42 g, 0.58 mmol, 0.1 eq.) were dissolved in 20 mL of anhydrous THF, then allyl isocyanate (0.72 mL, 8.79 mmol, 1.5 eq.) was added and the mixture was stirred for three hours at 60 °C under inert atmosphere (Argon). The reaction solution was evaporated, dissolved in dichloromethane (50 mL), and extracted with 1N HCl (20 mL x2) and brine (20 mL x2). The organic layers were collected and dried over $MgSO_4$ and evaporated. The product is obtained in the form of a powder, yield: 93 %.

HRMS: Peak mass: 424.17682; Theoretical mass: 424.17656

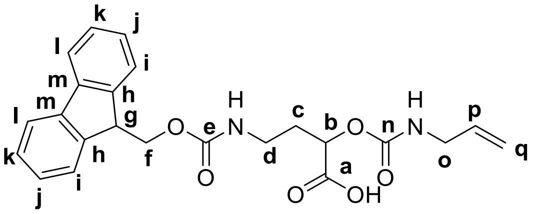

^{13}C NMR (MeOD, 125 MHz): δC 173.2 (a) 157.4 (e), 156.9 (n), 143.9 (h), 141.2 (m), 134.3 (r), 127.2 (i), 126.7 (j), 124.6 (k), 119.6 (l), 114.2 (q), 69.8 (b), 66.4 (f), 40.3 (g), 36.7 (o), 32.3 (c), 31.1 (d).

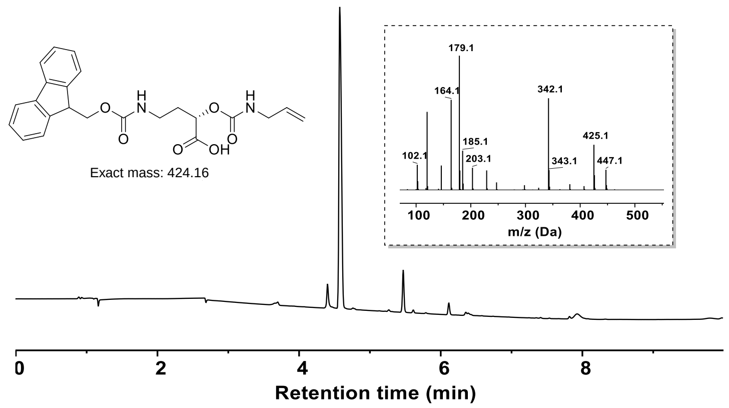

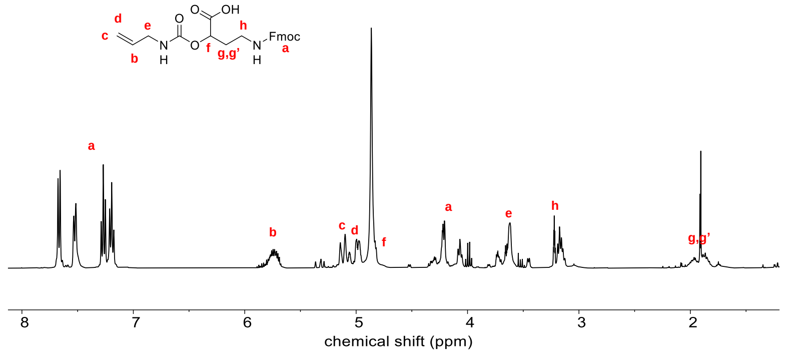

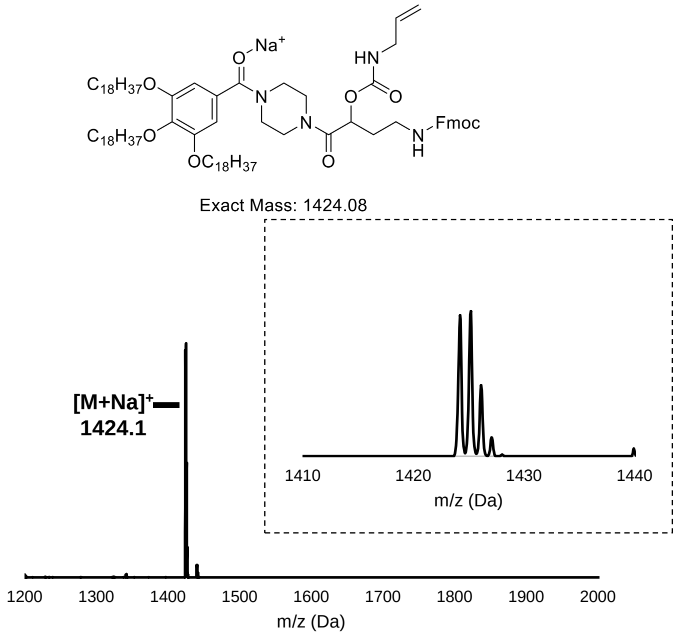

Figure S25: Structure of allylcarbamoyl-functionalized amino acid and LC-MS chromatogram at 214 nm; ^{1}H NMR 400 MHz, MeOD of allylcarbamoyl-functionalized amino acid; coupling test of monomer on non-cleavable support and MALDI-ToF of the structure. MS spectra in the dashed line are obtained from integration of the peak shown in LC-MS; main peaks are product and fragmentation.

Synthesis of (14): (R)-4-Fmoc(amino)-2-((furfurylcarbamoyl)oxy)butanoic acid

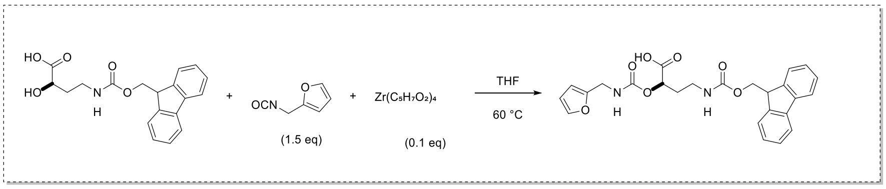

Scheme S15: Synthesis of furfurylcarbamoyl-functionalized amino acid.

4-Fmoc(amino)-2-hydroxybutanoic acid (2 g, 5.76 mmol, 1 eq.) and (Zr(acac)$_4$) (42 g, 0.58 mmol, 0.1 eq.) were dissolved in 20 mL of anhydrous THF, then furfuryl isocyanate (1.08 mL, 8.79 mmol, 1.5 eq.) was added and the mixture was stirred for three hours at 60 °C under inert atmosphere (Argon). The reaction solution was evaporated, dissolved in dichloromethane (50 mL), and extracted with 1N HCl (20 mL x2) and brine (20 mL x2). The organic layers were collected and dried over $MgSO_4$ and evaporated. The product is obtained in the form of a powder, yield: 65 %.

HRMS: Exact Mass: 463.15125; Theoretical Mass: 463.15107.

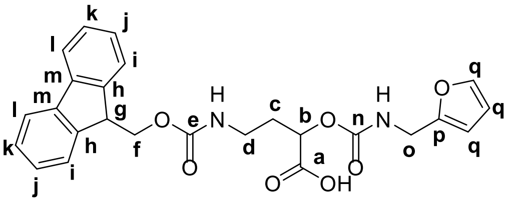

^{13}C NMR (MeOD, 125 MHz): δC 173.2 (a) 157.4 (e), 156.9 (n), 144.2 (p), 143.9 (h), 141.2 (m,q), 127.2 (i), 126.7 (j), 124.6 (k), 119.6 (l),110.2 (q) 69.8 (b), 66.4 (f), 40.3 (g), 38.9 (o), 32.3 (c), 31.1 (d).

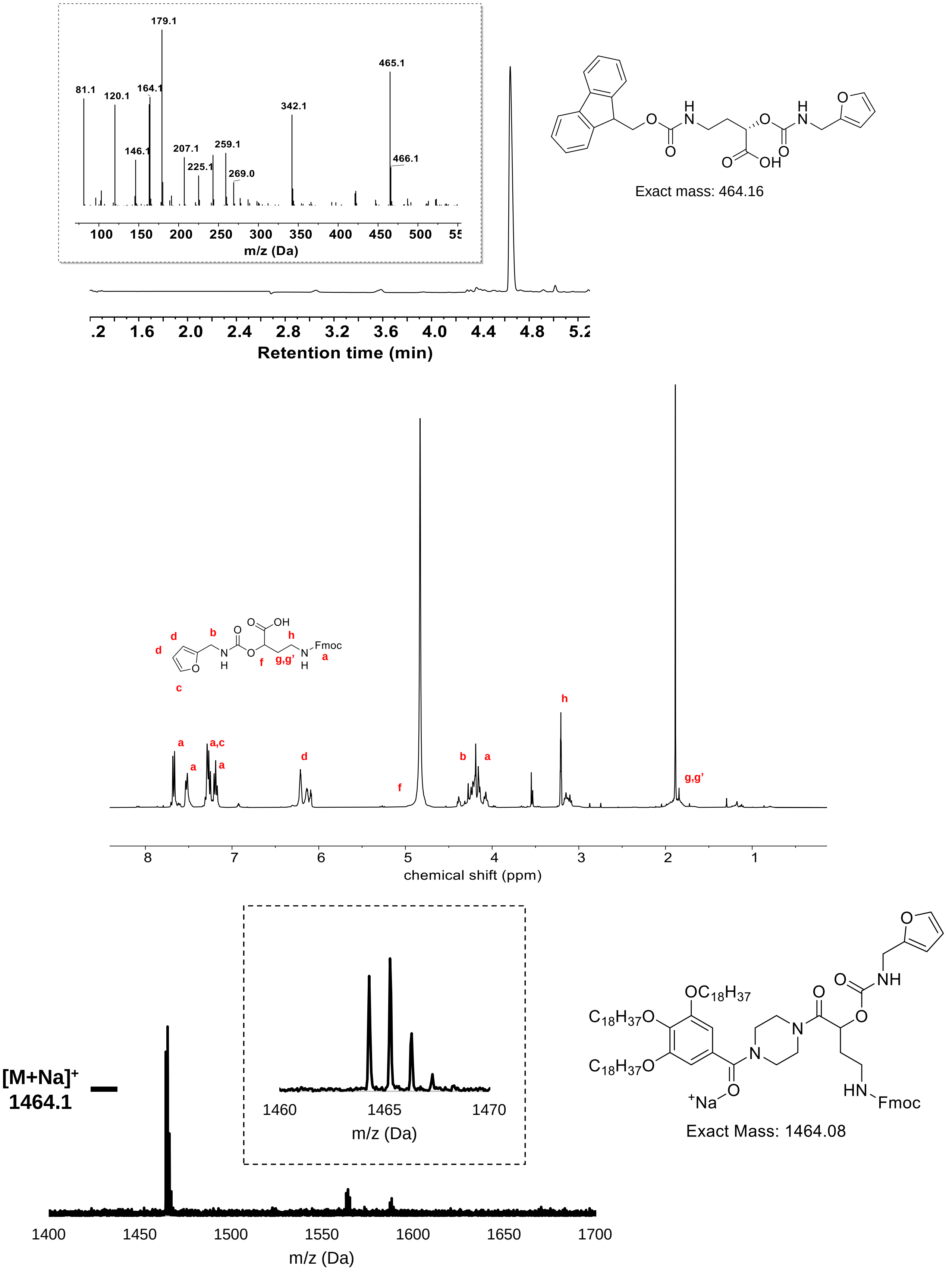

Figure S26: Structure of furfurylcarbamoyl-functionalized amino acid and LC-MS chromatogram at 214 nm; ^{1}H NMR 400 MHz, MeOD of furfurylcarbamoyl-functionalized amino acid; coupling test of monomer on non-cleavable support and MALDI-ToF of the structure. MS spectra in the dashed line are obtained from integration of the peak shown in LC-MS; main peaks are product and fragmentation.

Synthesis of (15): 4-Fmoc(amino)-2-((thiolactonecarbamoyl)oxy)butanoic acid

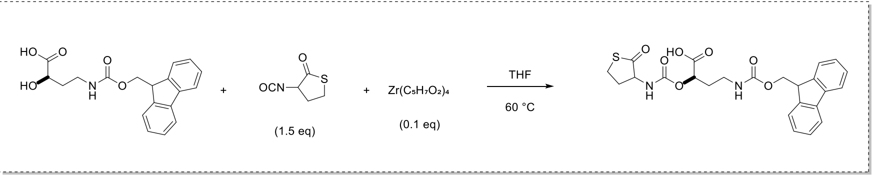

Scheme S16: Synthesis of thiolactone carbamoyl-functionalized *amino acid.*

4-Fmoc(amino)-2-hydroxybutanoic acid (2 g, 5.76 mmol, 1 eq.) and ($Zr(acac)_4$) (42 mg, 0.58 mmol, 0.1 eq.) were dissolved in 20 mL of anhydrous THF, then 4-fluoro phenyl isocyanate (0.960 mL, 8.79 mmol, 1.5 eq) was added and the mixture was stirred for three hours at 60 °C under inert atmosphere (Argon). The reaction solution was evaporated, dissolved in dichloromethane (50 mL), and extracted with 1N HCl (20 mL x2) and brine (20 mL x2). The organic layers were collected and dried over $MgSO_4$ and evaporated. The product is obtained in the form of a powder, yield: 80 %.

HRMS: Peak Mass: 485.13693; Theoretical Mass: 485.13770.

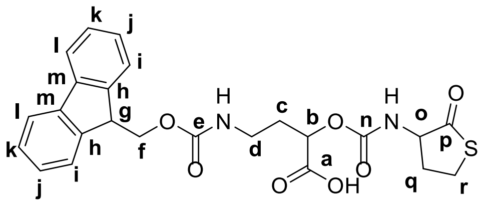

^{13}C NMR (MeOD, 125 MHz): δC 206.2 (p),173.2 (a) 157.4 (e), 156.9 (n), 143.9 (h), 141.2 (m), 127.2 (i), 126.7 (j), 124.6 (k), 119.6 (l), 69.8 (b), 66.4 (f), 59.7 (o) 40.3 (g), 36.7 (o), 32.3 (c), 31.1 (d), 31.4 (r), 26.4 (q).

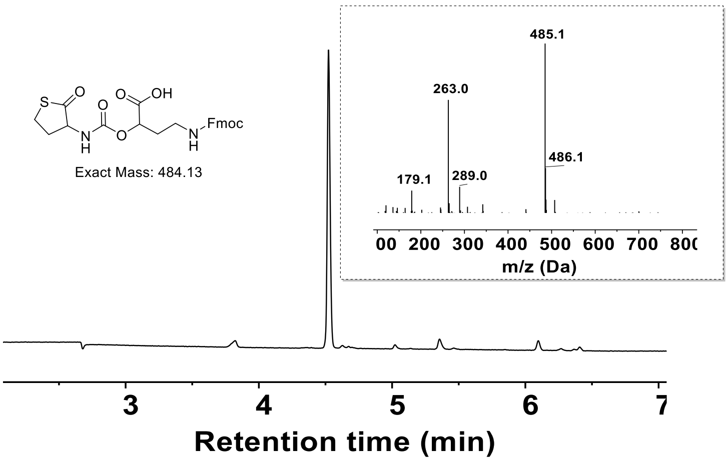

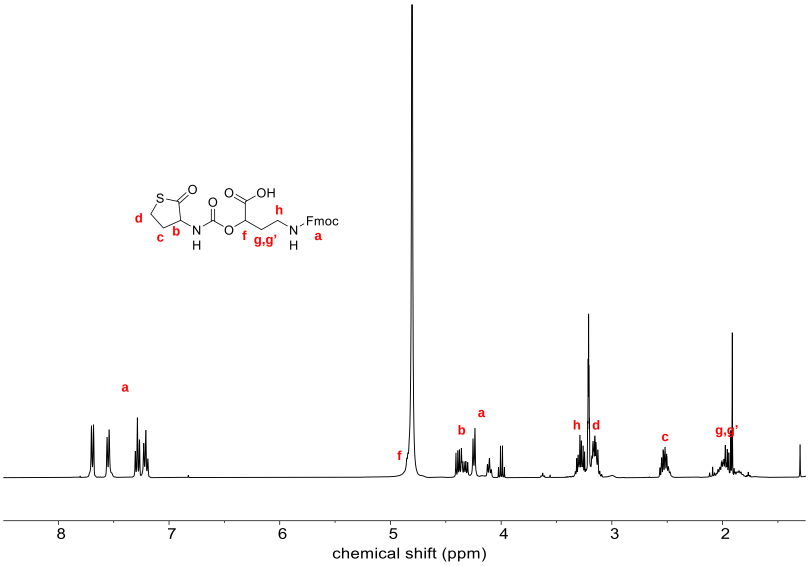

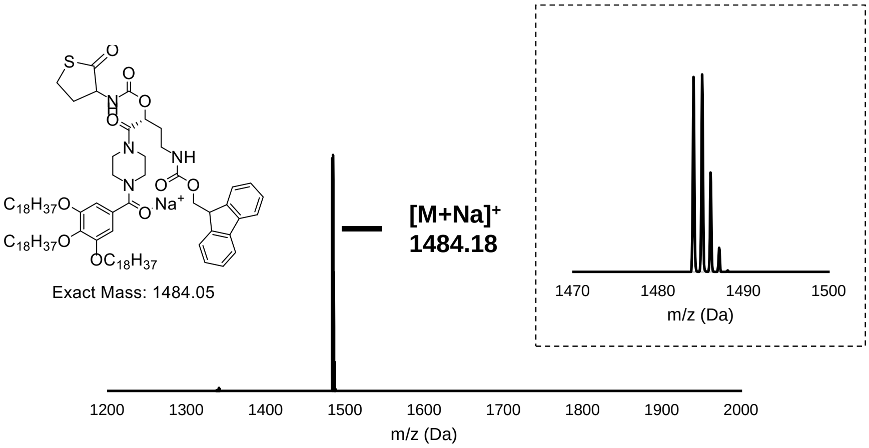

Figure S27: Structure of thiolactone carbamoyl-functionalized amino acid and LC-MS chromatogram at 214 nm; ^{1}H NMR 400 MHz, MeOD of thiolactone carbamoyl-functionalized amino acid; coupling test of monomer on non-cleavable support and MALDI-ToF of the structure. MS spectra in the dashed line are obtained from integration of the peak shown in LC-MS; main peaks are product and fragmentation.

Synthesis of (16): 4-Fmoc(amino)-2-TBDMS(hydroxy)butanoic acid

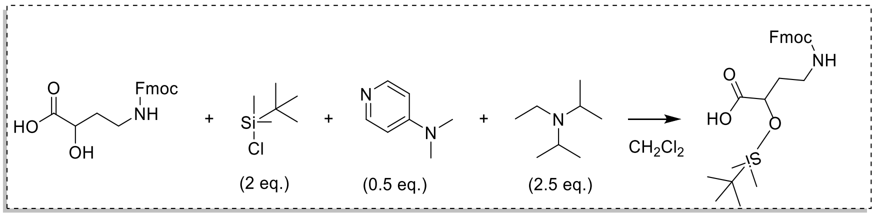

Scheme S17: TBDMS-Cl protection of 4-Fmoc(amino)-2-hydroxybutyric acid.

To a solution of 4-Fmoc(amino)-2-hydroxybutanoic acid (500 mg, 1.46 mmol, 1 eq.) in dichloromethane (10 mL) was added t-butyldimethlsilyl chloride (441 mg, 2.93 mmol, 2 eq.), diisopropylethylamine (0.64 mL, 3.66 mmol, 2.5 eq.) and dimethylaminopyridine (88 mg, 0.73 mmol, 0.5 eq.). The resulting reaction mixture was allowed to stir at room temperature for 1.5 h, after which time it was diluted with an additional 300 mL of dichloromethane and washed with 0.1 M citric acid (2 x 300 mL) and brine (3x 300 mL). The resulting yellow organic layer was dried over $MgSO_4$ and concentrated, a white solid was obtained, yield: 60 %.

HRMS: Peak Mass: 456.22000; Theoretical mass: 456.2208

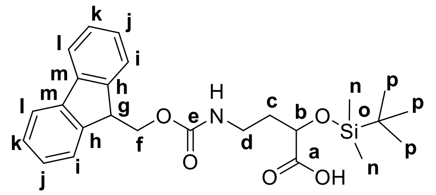

^{13}C NMR (MeOD, 125 MHz): δC 173.2 (a) 157.4 (e), 143.9 (h), 141.2 (m), 127.2 (i), 126.7 (j), 124.6 (k), 119.6 (l), 69.8 (b), 66.4 (f), 40.3 (g), 32.3 (c), 31.1 (d), 30.6 (o), 25.1 (p), -4.2 (n).

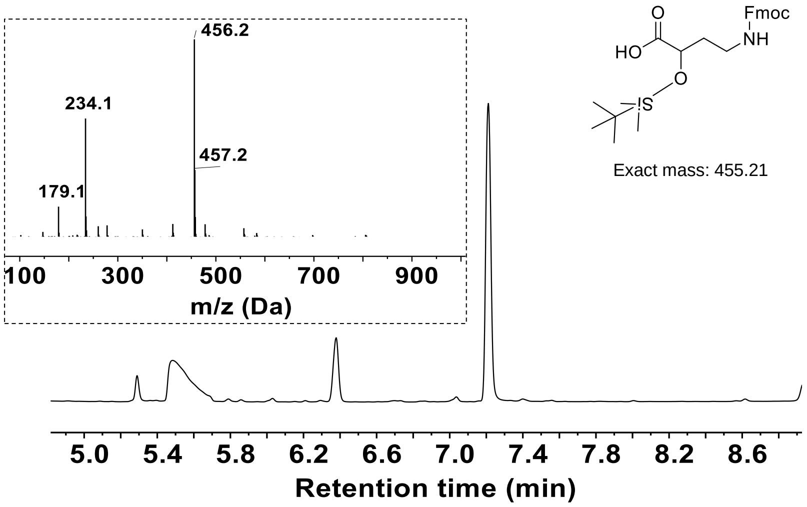

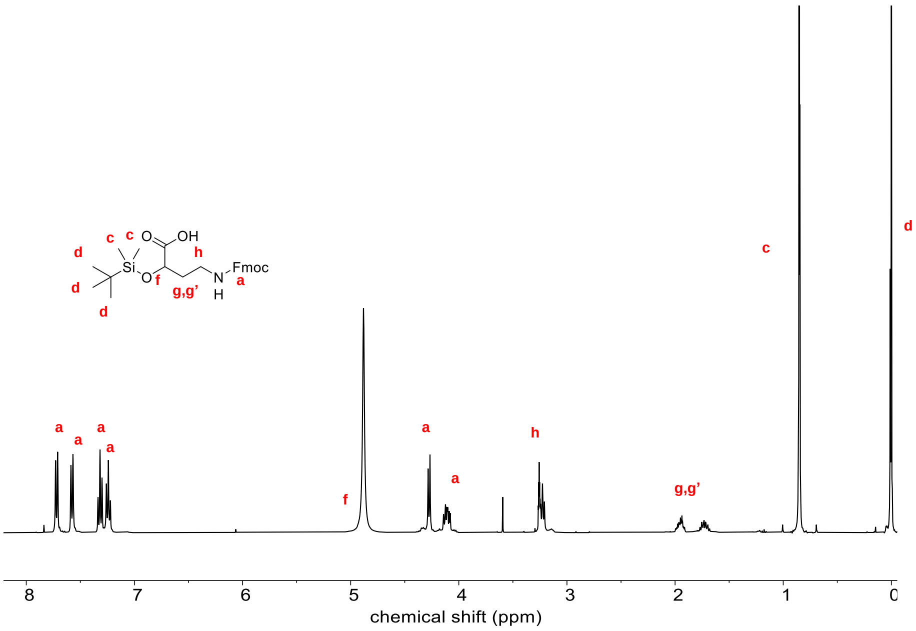

Figure S28: Structure of 4-Fmoc(amino)-2-TBDMS(hydroxy)butanoic acid and LC-MS chromatogram at 214 nm; ^{1}H NMR 400 MHz, MeOD of 4-Fmoc(amino)-2-TBDMS(hydroxy)butanoic acid. MS spectra in the dashed line are obtained from integration of the peak shown in LC-MS; main peaks are product and fragmentation.

Synthesis of (17): 4-Fmoc(amino)-2-THP(hydroxy)butanoic acid

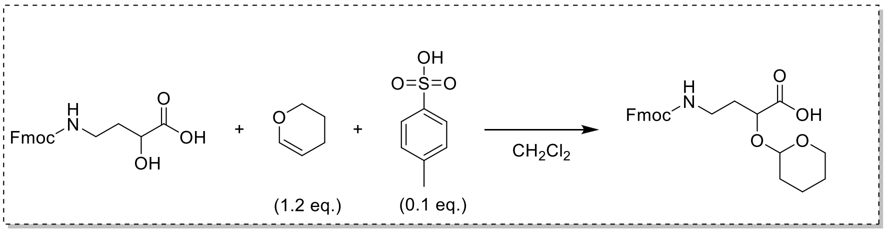

Scheme S18: Synthesis of 4-Fmoc(amino)-2-THP(hydroxy)butanoic acid.

A solution of 4-Fmoc(amino)-2-hydroxybutanoic acid (500 mg, 1.46 mmol, 1 eq.), 3,4-dihydro-2H-pyran (0.18 mL, 1.76 mmol, 1.2 eq.) and p-toluenesulfonic acid (25mg, 0.15 mmol, 0.1 eq.) is stirred in CH_2Cl_2 at room temperature for 2 h. DCM is then evaporated and an aqueous solution of $NaHCO_3$ is added to the mixture and extraction is performed using EtOAc (x3). Next, the combined organic layers are washed with brine, dried with $MgSO_4$ filtered and dried under vacuum after evaporation of the solvent. Yield 80 %, white powder.

HRMS: Peak mass: 425.17084; Theoretical mass: 425.17071

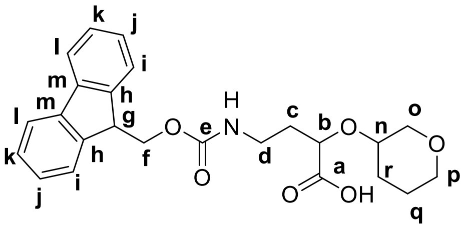

^{13}C NMR (MeOD, 125 MHz): δC 173.2 (a), 157.4 (e), 143.9 (h), 141.2 (m), 127.2 (i), 126.7 (j), 124.6 (k), 119.6 (l), 94.2 (n), 69.8 (b), 66.4 (f), 40.3 (g), 32.3 (c), 31.1 (d), 29.8 (r), 24.6 (p), 18.1 (q)

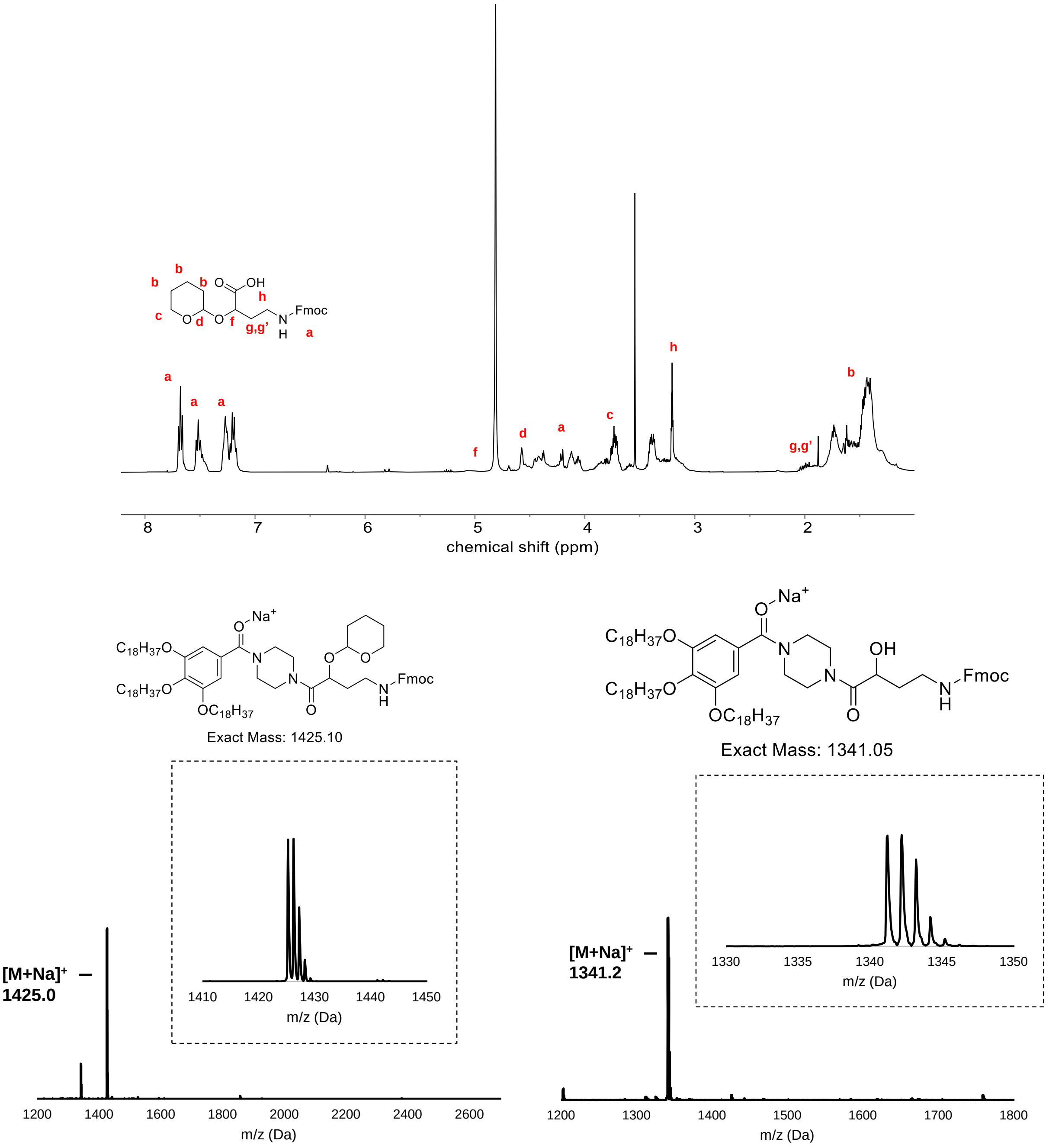

Figure S29: ¹H NMR 400 MHz, MeOD of 4-Fmoc(amino)-2-THP(hydroxy)butanoic acid. Coupling of α-THP protected amino acid and deprotection verified via MALDI-ToF. Coupling test of monomer on non-cleavable support and THP deprotection MALDI-ToF of the structures.

E. Synthesis of amide sequence-defined octamer on RASS

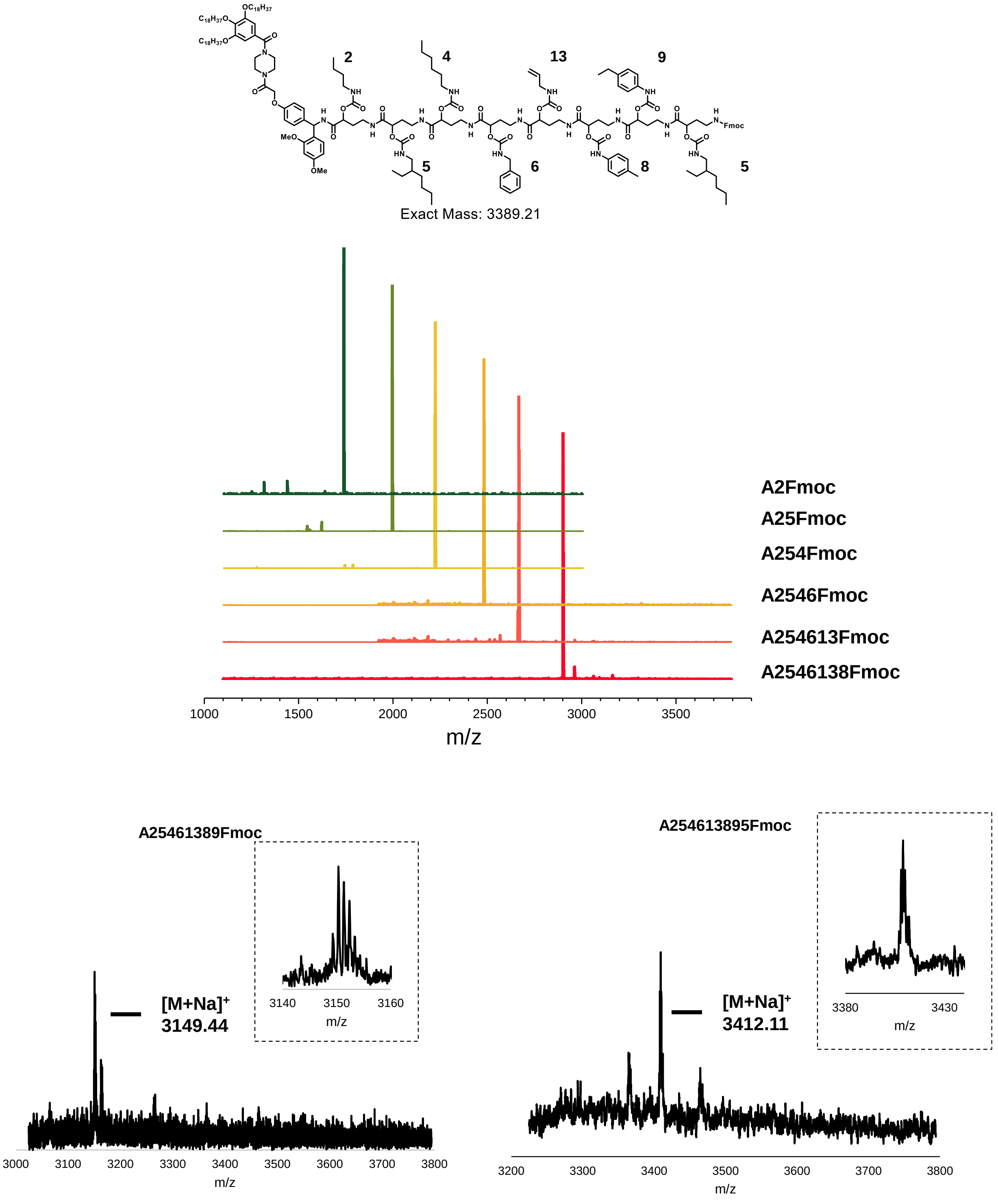

Figure S30: Structure of sequence-defined octamer synthesized in 17 steps (top); MALDI-ToF evolution until the hexamer (middle). Because of too high noise, MALDI-ToF spectra of the heptamer and octamer are given separately (bottom).

F. Synthesis of thiolactone-based sequence-defined tetramer on RASS

Tla-Rink amide soluble support

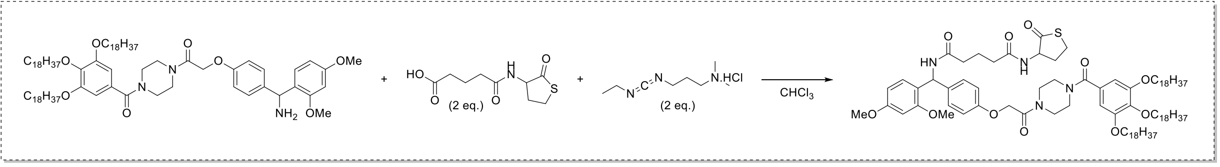

As already reported,[1] Rink amide soluble support (9 g, 6.95 mmol, 1 eq.) and COOH-Tla (3.21 g, 13.9 mmol, 2 eq.) were dissolved in 100 mL of chloroform under inert atmosphere (argon). After complete dissolution, EDC-Cl (2.66 g, 13.9 mmol, 2 eq.) was dissolved in 5 mL of $CHCl_3$. The reaction mixture is cooled down to 0°C and EDC-Cl solution is added dropwise to the reaction under constant stirring. The reaction proceeded for 2 hours at room temperature and conversion was checked via MALDI-ToF. Approximately 50 mL of the solvent is removed in vacuo, before the mixture is precipitated in cold methanol. After filtration, the residue was washed with additional cold methanol and subsequently dried in a vacuum oven, yielding the product as a white powder. Yield: 10.1 g white solid (96.6 %). MW: 1508.28 g/mol

Aminolysis-thiol-ene coupling

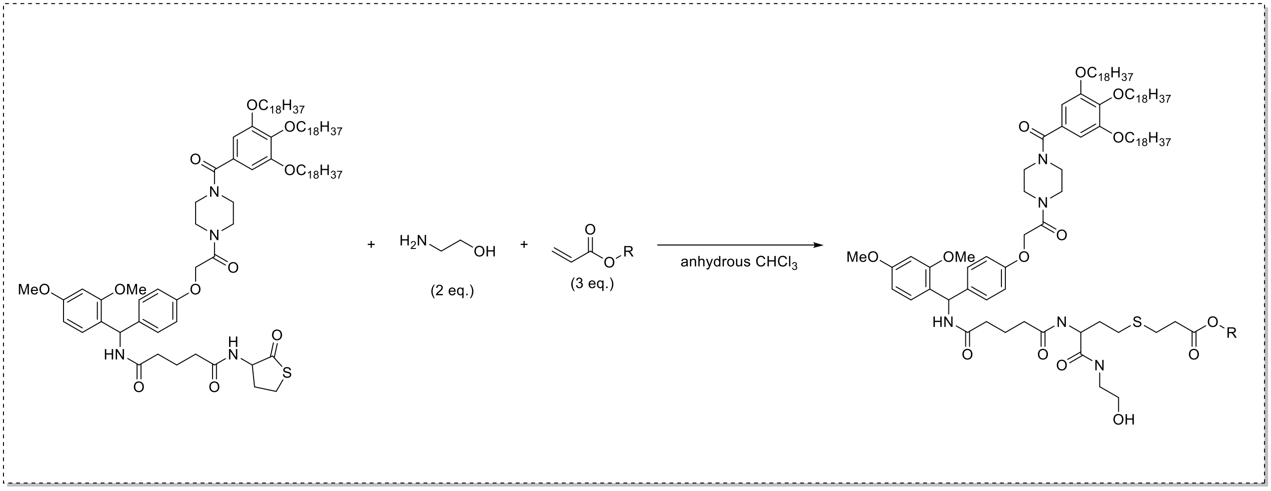

The Rink Amide Soluble Support is placed in a 250 mL round bottom flask with two necks, dissolved in anhydrous $CHCl_3$ with an average concentration of 0.175 M and left stirring under Argon atmosphere. Then acrylate (2 eq.) is added in the stirring solution followed by ethanolamine (3 eq.). The reaction is left stirring for 2 hours for Rink Amide support, and the conversion is checked via MALDI-ToF. When the reaction is finished, almost half of the solvent is evaporated in the rotavapor and the reaction mixture is precipitated in cold MeOH, allowing the recovery of the product attached to the soluble support in the form of a white powder. The product is washed through the filter with additional MeOH and is dried in a vacuum oven at 40 °C.

Chain extension with Tla-NCO

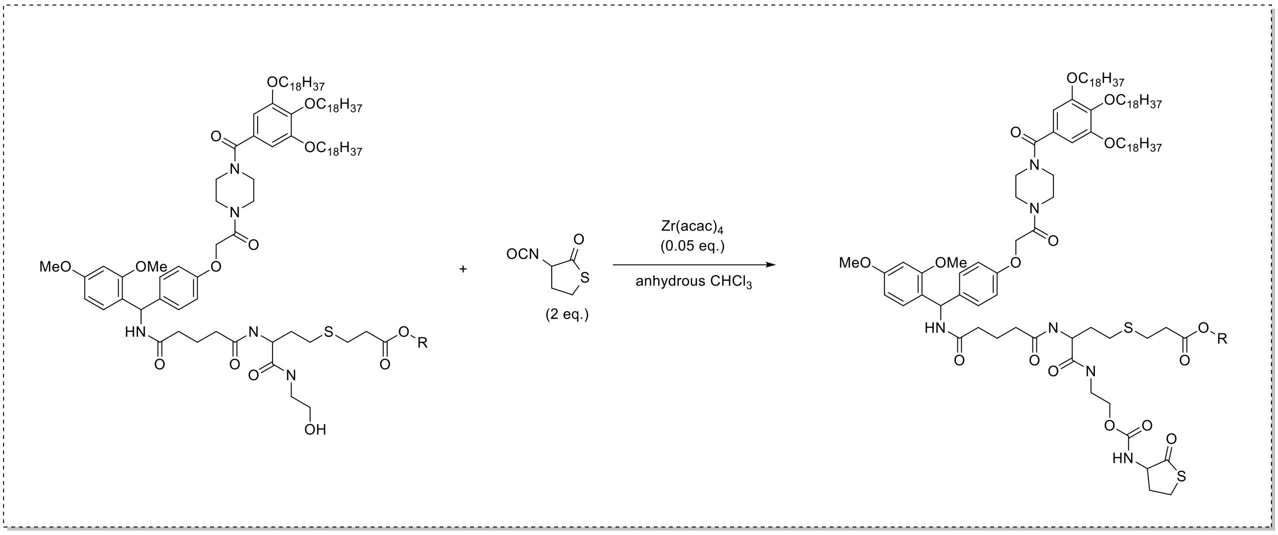

The Rink Amide Soluble Support and Zr(acac)$_4$ (0.05 eq.) were placed in a 250 mL round bottom flask with two necks, dissolved in anhydrous $CHCl_3$ with an average concentration of 0.175 M and left stirring under Argon atmosphere. Then Tla-NCO (1.2 eq.) was added in the stirring solution. The reaction is left stirring for 2 hours and the conversion is checked via MALDI-ToF. When the reaction is finished, almost half of the solvent is evaporated in the rotavapor and the reaction mixture is precipitated in cold MeOH, allowing the recovery of the product attached to the soluble support in the form of a white powder. The product is washed through the filter with additional MeOH and is dried in a vacuum oven at 40 °C.

Synthesis of sequence-defined thiolactone tetramer

The sequence-defined thiolactone-based tetramer is synthesized according to the protocol described in **Paragraph F.II and F.III** of the Supporting information. For the aminolysis thiol-ene step, the acrylates used are: 1) butyl acrylate, 2) benzyl acrylate, 3) hexyl acrylate), 4) 2-ethylhexyl acrylate.

Cleavage of tetramer from RASS

A solution containing sequence-defined thiolactone tetramer in 10% TFA in CH_2Cl_2 with 2 % TIPS and 2 % H_2O is left stirring for one hour. Afterwards the solution is diluted with water to get rid of TFA, the organic phase is collected, concentrated via evaporation, and precipitated in methanol. The white precipitate is the Cleaved support, whereas the organic solution is recollected and dried to obtain the cleaved sequence-defined thiolactone-based tetramer.

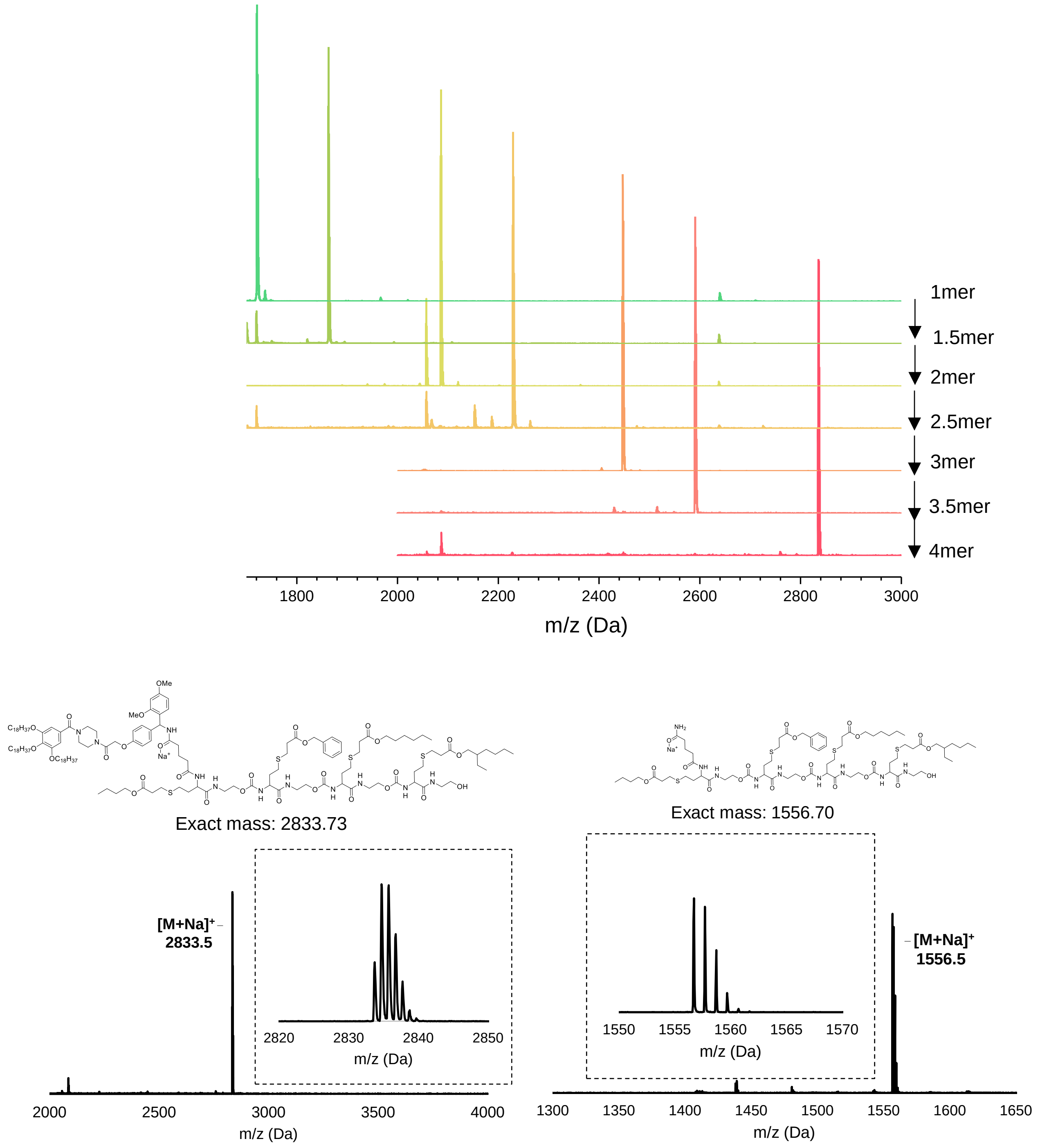

*Figure S31: MALDI-ToF evolution of sequence-defined tetramer based on thiolactone protocol (**T-2645**) and MALDI-ToF of the cleaved tetramer **T-2645**.*

G. Synthesis of sequence-defined tetramers A2456 and A2546

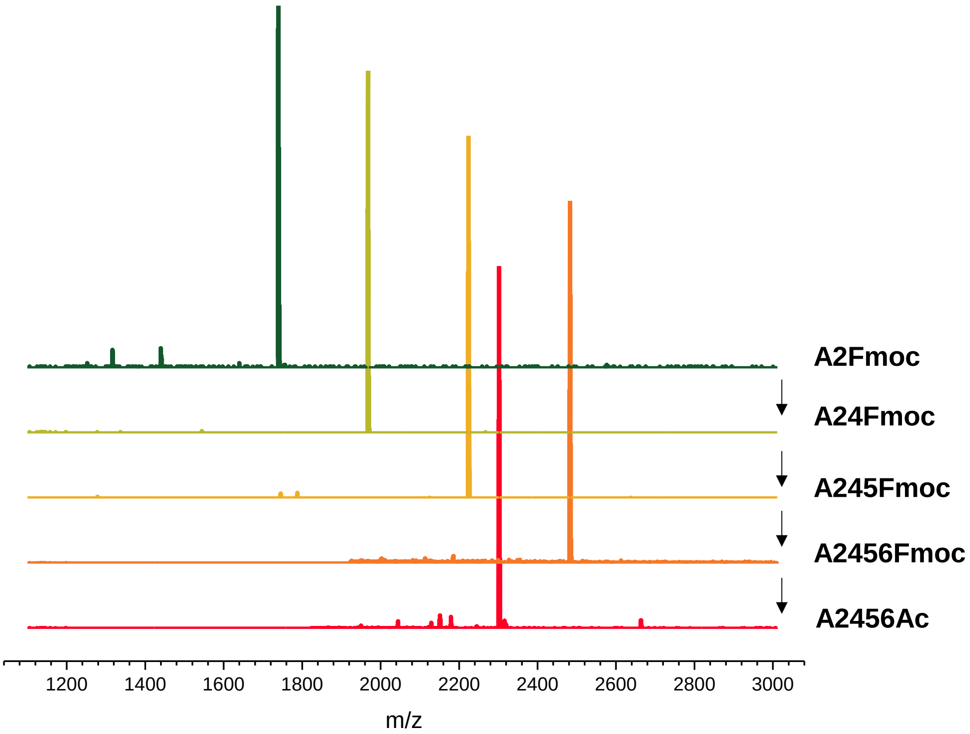

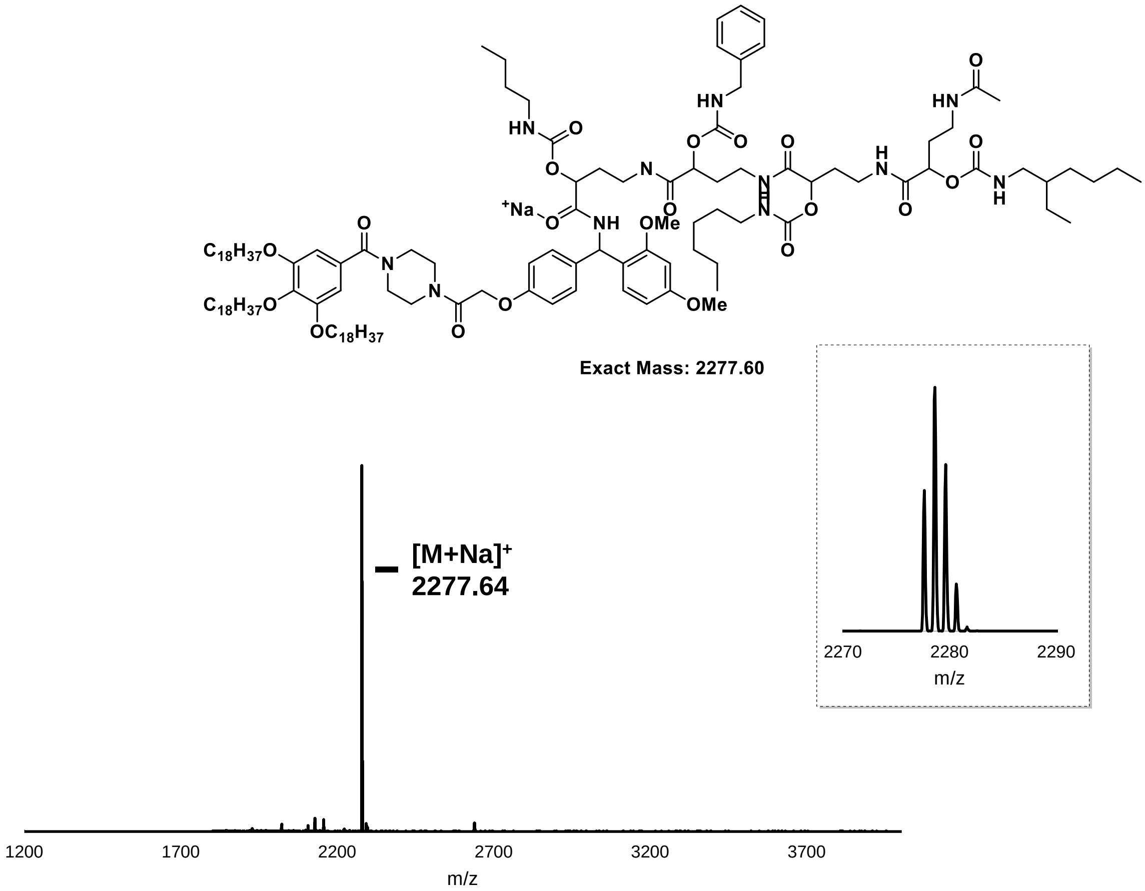

Figure S32: MALDI-ToF evolution of sequence-defined tetramer based on oligoamide protocol (A2456); Structure and MALDI-ToF of RASS-A2456Ac.

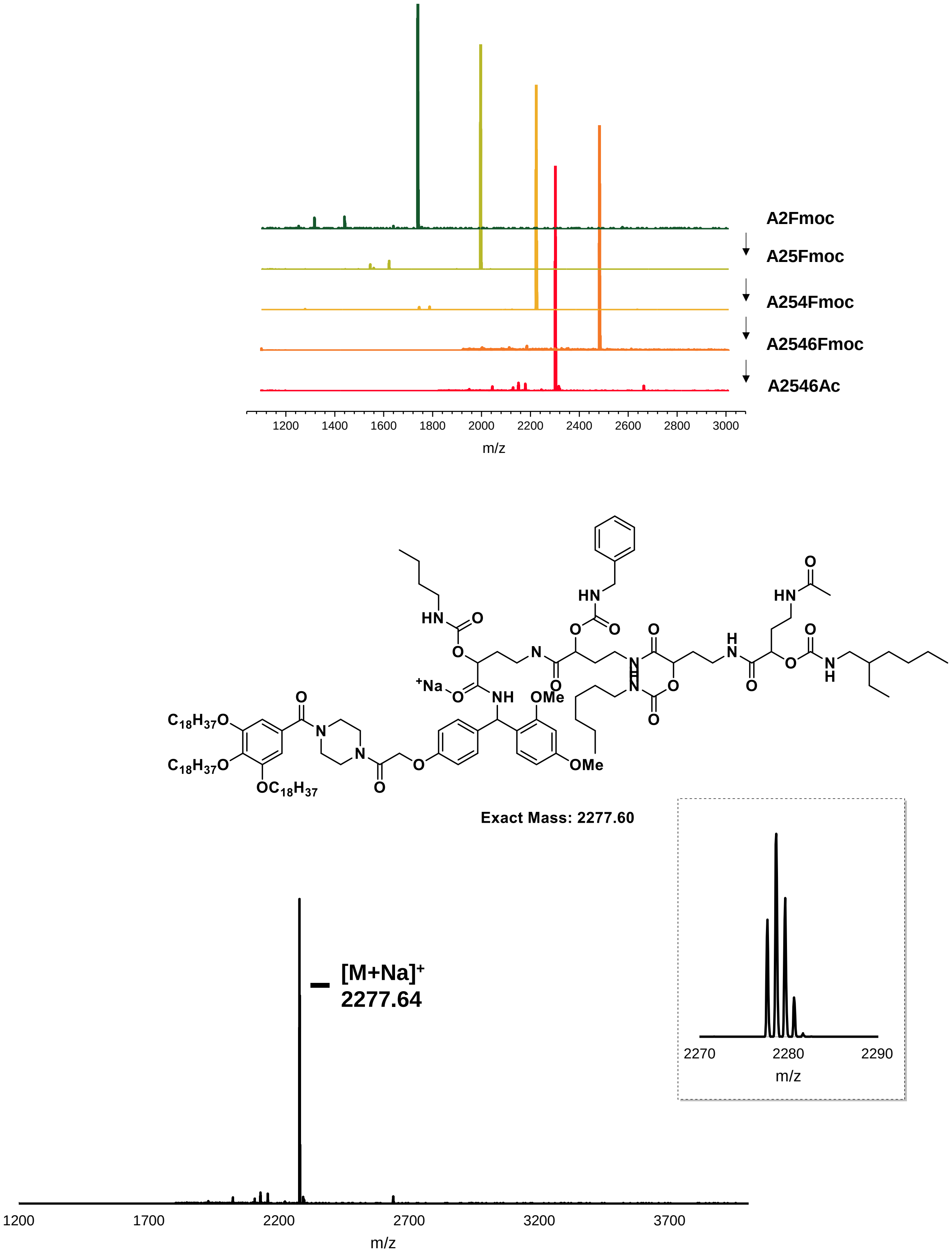

Figure S33: MALDI-ToF evolution of sequence-defined tetramer based on oligoamide protocol (A2546Ac); Structure and MALDI-ToF of RASS-A2564Ac.

H. Synthesis of bidirectional RASS

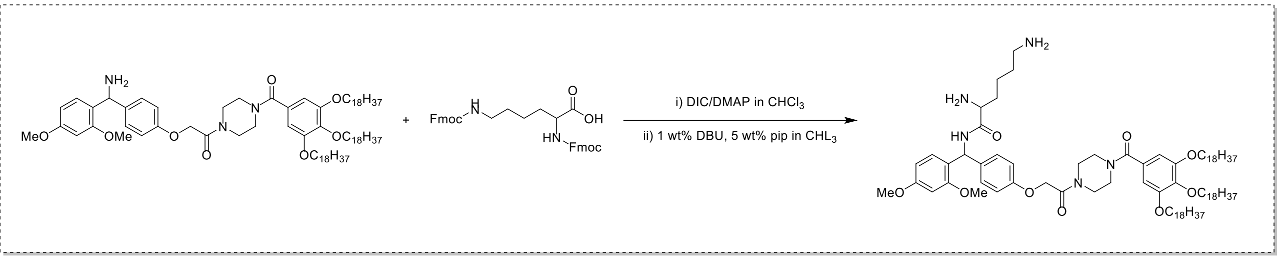

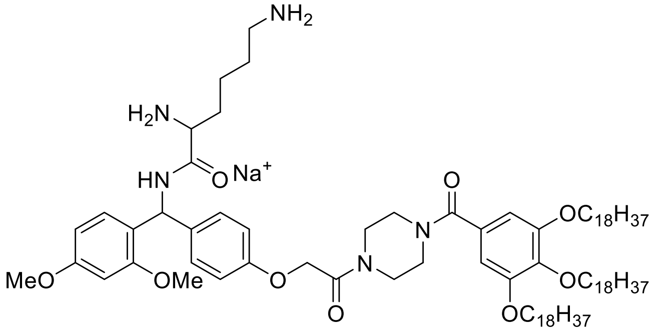

Exact mass: 1445.14

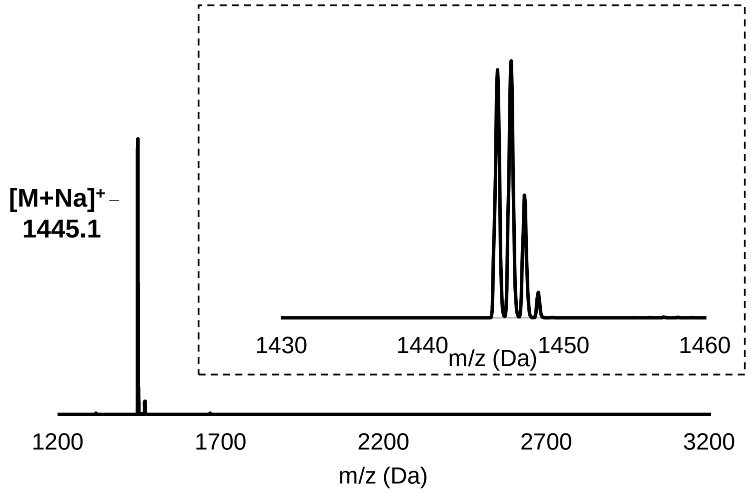

Figure S34: Structure of Bidirectional RASS and MALDI-ToF.

I. Synthesis of telechelic sequence-defined oligoamide-urethane heptamers

Oligomer AcGG8L8GGAc

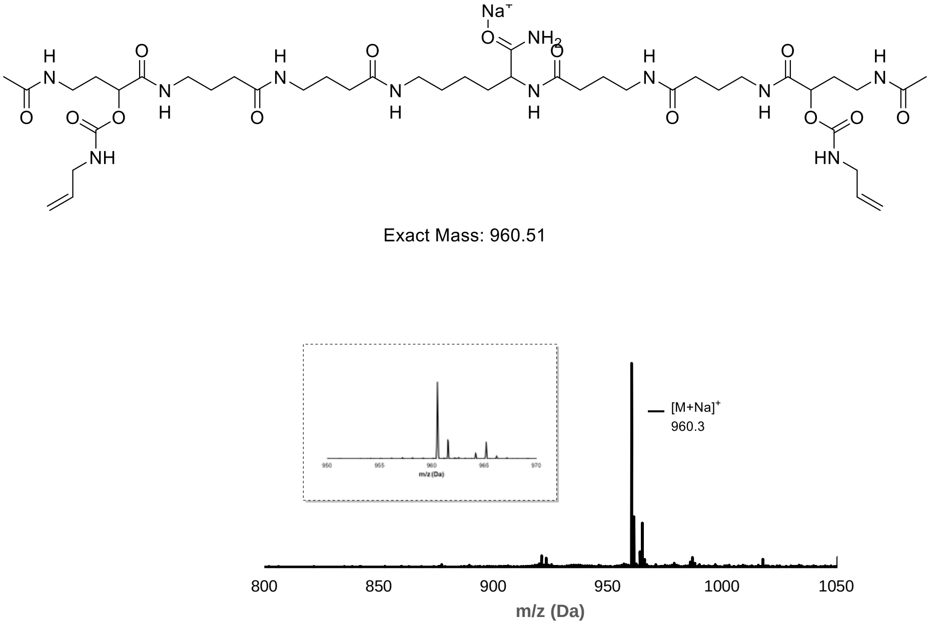

Figure S35: Structure and MALDI-ToF of cleaved heptamer Ac8GGLGG8Ac.

Oligomer Ac8GGLGG8Ac

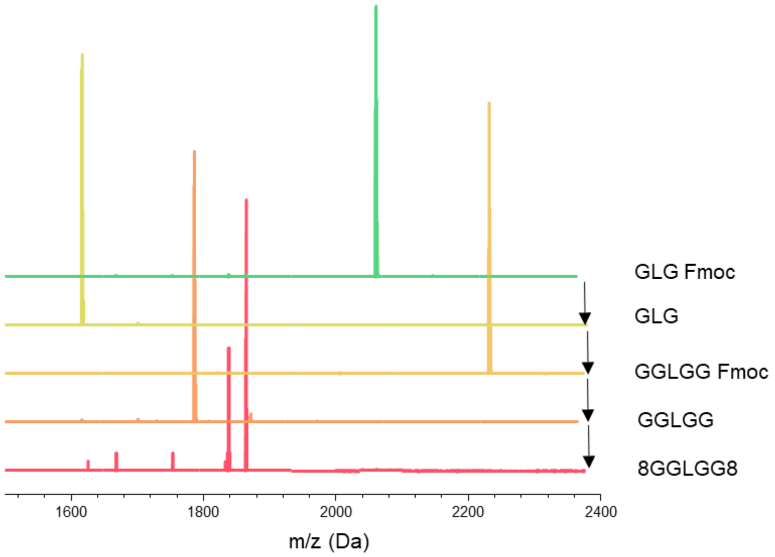

Figure S36: MALDI-ToF evolution of 8GGLGG8 heptamer.

Oligomer AcG8GLG8Gac

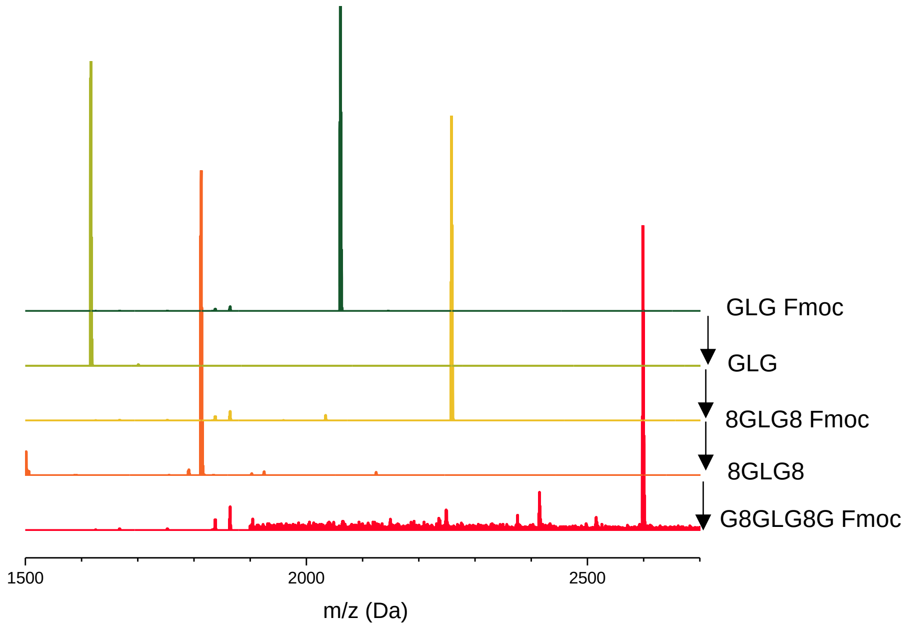

Figure S37: MALDI-ToF evolution of G8GLG8G heptamer.

J. Synthesis of telechelic sequence-defined oligoamides for network formation

I. PeLPe

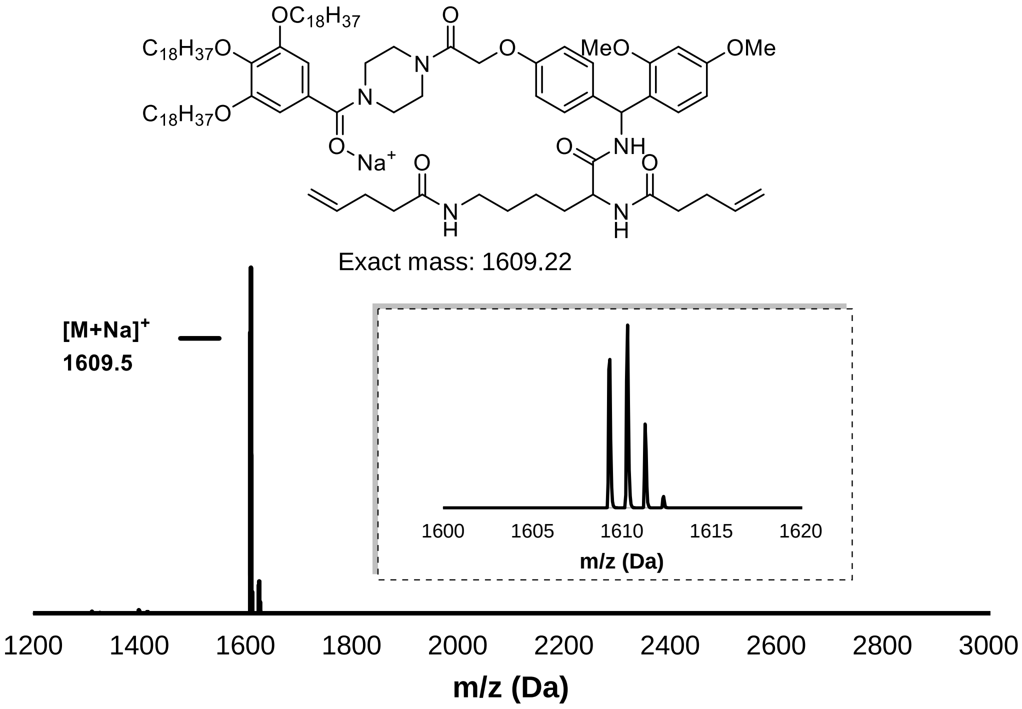

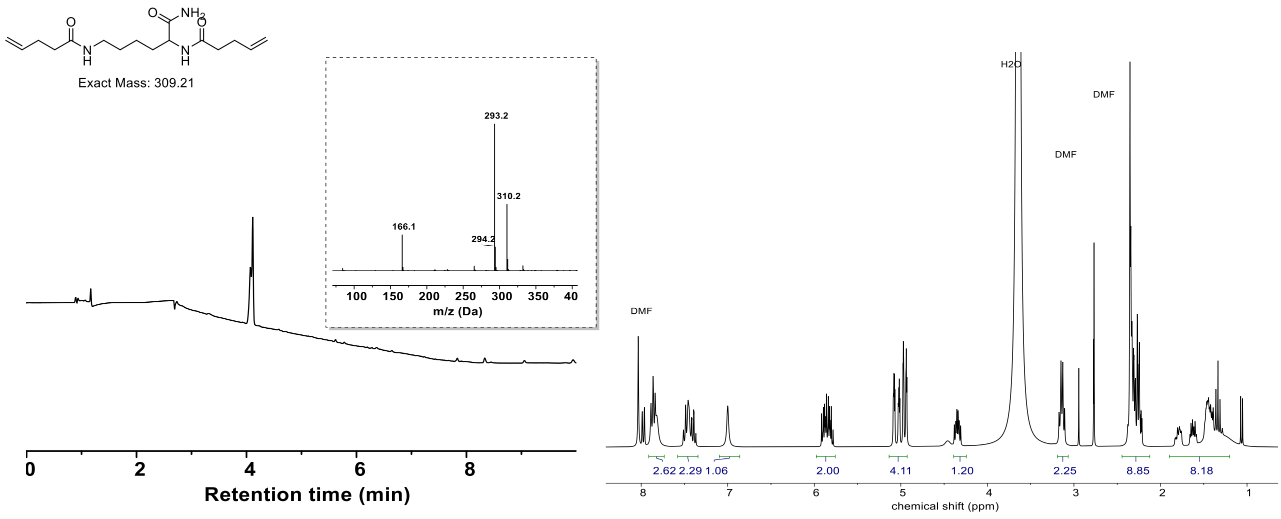

Figure S38: Structure of PeLPe trimer on RASS and MALDI-ToF. LC-MS and ^{1}H-NMR 400 MHz in $CDCl_3$ of cleaved trimer.

II. PeGLGPe

Exact mass: 1705.30

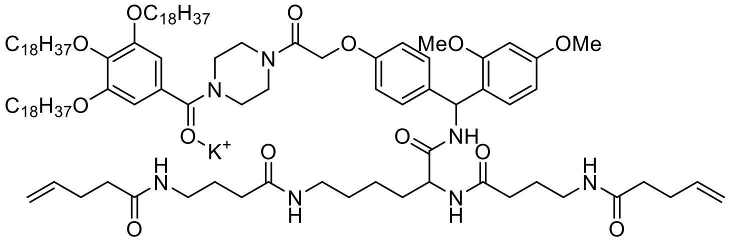

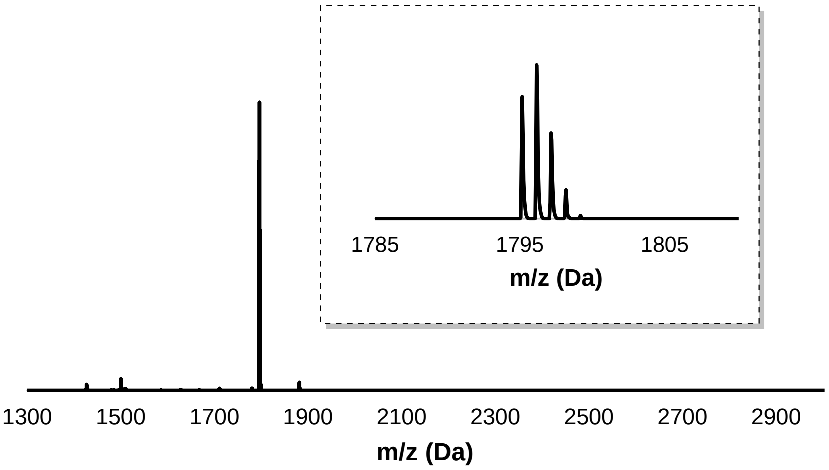

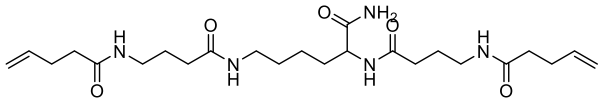

Exact mass: 479.31

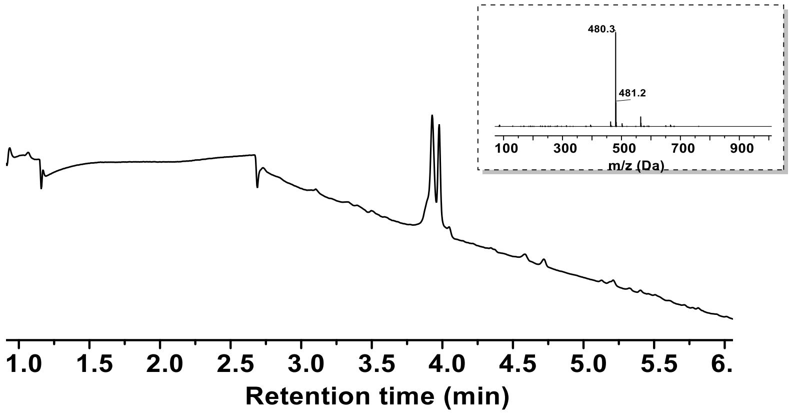

Figure S39: Structure of PeGLGPe pentamer on RASS and MALDI-ToF. LC-MS and ^{1}H-NMR 400 MHz in $CDCl_3$ of cleaved pentamer.

K. General procedure for network formation

Crosslinkers (PeGLGPe and PeLPe) powders are dissolved in methanol (ca. 200 mg/mL). Then pentaerythritol tetrakis(2-mercaptoacetate) (1 eq. to allyl moiety) and dimethoxyethylacetophenone (DMPA, 0.01 eq. to allyl moiety) are added and the solution is left to stir in a vial. The network formation occurs under 365 nm UV irradiation for 1 h. Then the resulting networks are further dried at 40 °C in a vacuum oven to evaporate the methanol.

L. Characterization of the networks

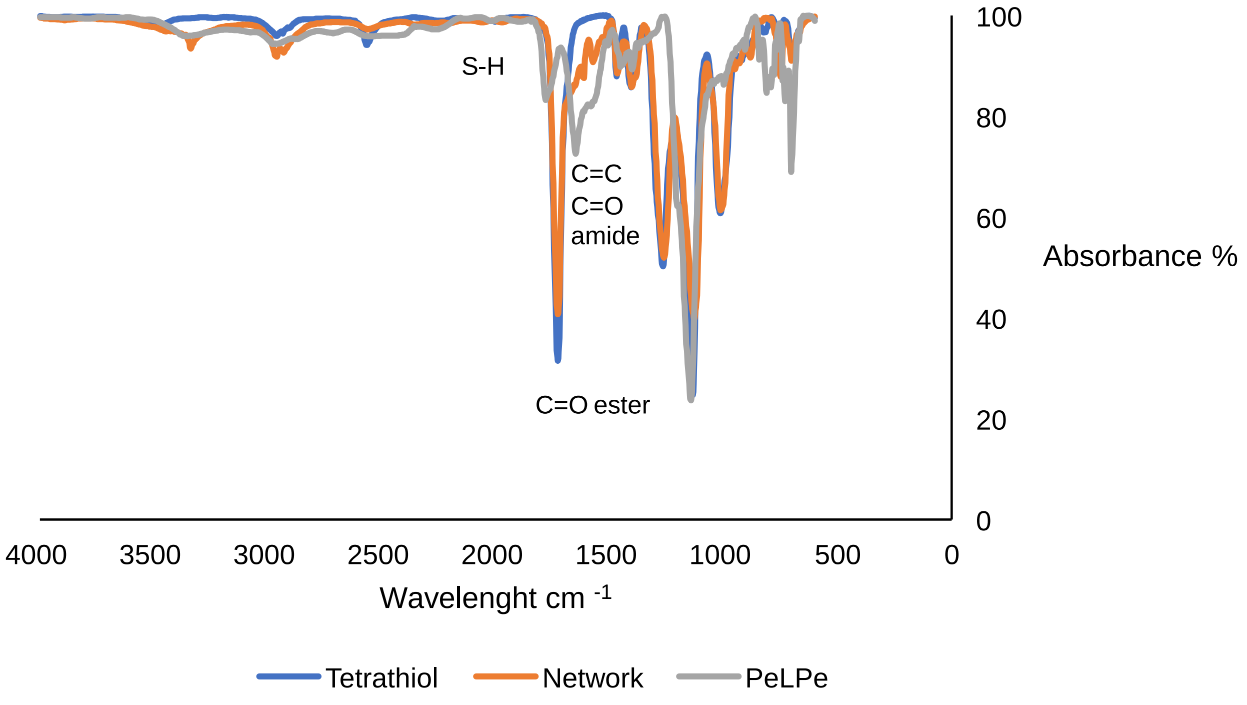

Figure S40: FT-IR of tetrathiol (blue), trimer diallyl crosslinker PeLPe (grey) and resulting network (orange).

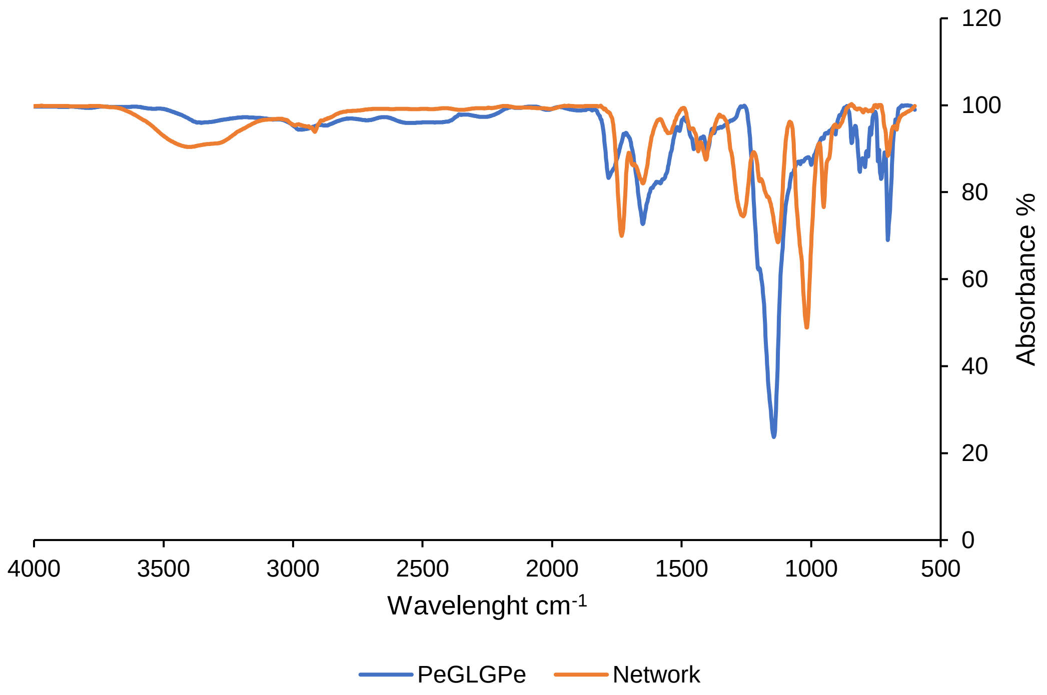

Figure S41: FT-IR of pentamer diallyl crosslinker PeGLGPe (blue) and resulting network (orange).

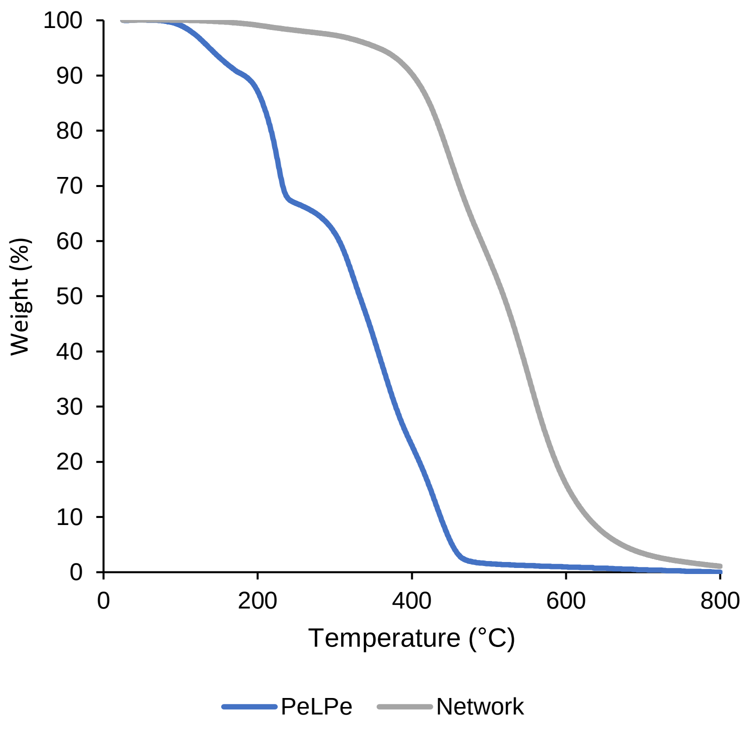

Figure S42: TGA of trimer PeLPe before curing (blue) and after curing with tetrathiol (grey).

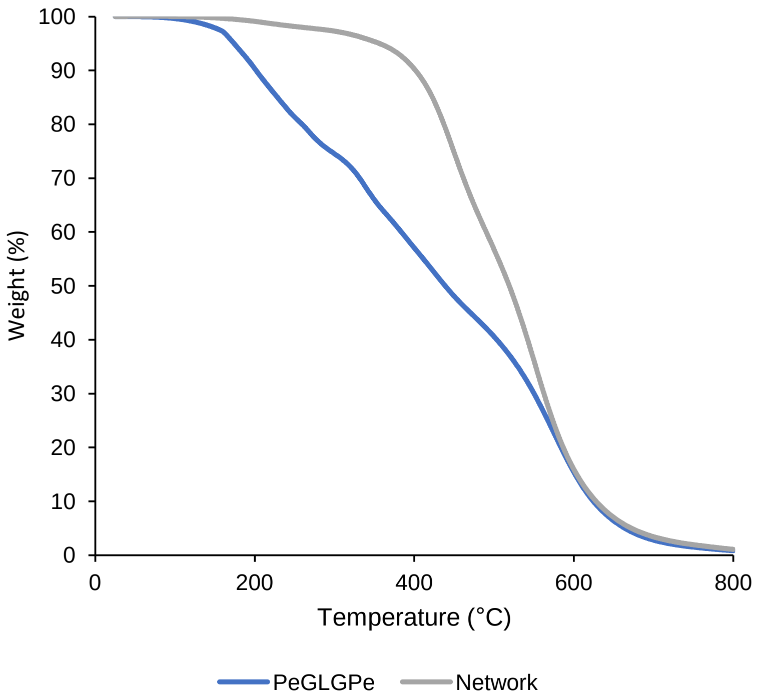

Figure S43: TGA of trimer PeGLGPe before curing (blue) and after curing with tetrathiol (grey).

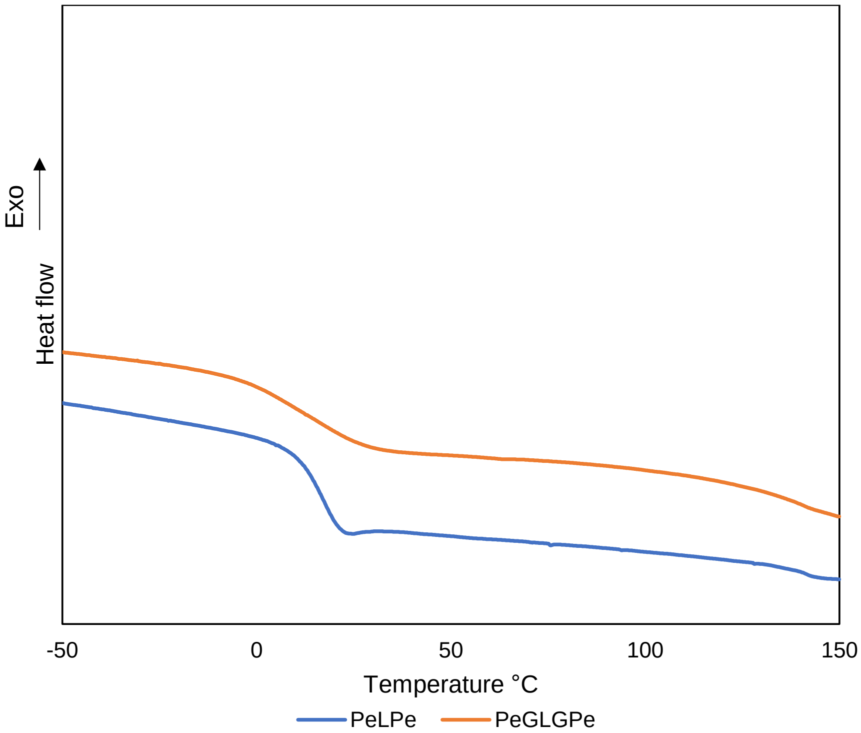

Figure S44: DSC comparison between two networks: network formed with trimer PeLPe and tetrathiol (blue) and pentamer PeGLGPe and tetrathiol (orange).

References

1. De Franceschi, I.; Mertens, C.; Badi, N.; Du Prez, F., Uniform soluble support for the large-scale synthesis of sequence-defined macromolecules. *Polymer Chemistry* **2022,** *13* (39), 5616-5624.
